# Design and Synthesis of Novel Symmetric Fluorene-2,7-Diamine Derivatives as Potent Hepatitis C Virus Inhibitors

**DOI:** 10.3390/ph14040292

**Published:** 2021-03-25

**Authors:** Mai H. A. Mousa, Nermin S. Ahmed, Kai Schwedtmann, Efseveia Frakolaki, Niki Vassilaki, Grigoris Zoidis, Jan J. Weigand, Ashraf H. Abadi

**Affiliations:** 1 Department of Pharmaceutical Chemistry, Faculty of Pharmacy and Biotechnology, German University in Cairo, Cairo 11835, Egypt; mai.abdelsadek@guc.edu.eg; 2 Faculty of Chemistry and Food Chemistry, Technische Universität Dresden, 01062 Dresden, Germany; Kai.Schwedtmann@chemie.tu-dresden.de (K.S.); jan.weigand@tu-dresden.de (J.J.W.); 3 Molecular Virology Laboratory, Hellenic Pasteur Institute, 11521 Athens, Greece; sevif@pasteur.gr (E.F.); nikiv@pasteur.gr (N.V.); 4 Department of Pharmacy, Division of Pharmaceutical Chemistry, School of Health Sciences, National and Kapodistrian University of Athens, 15771 Athens, Greece; zoidis@pharm.uoa.gr

**Keywords:** DAAs, 2,7-Diaminofluorene, HCV, NS5A inhibitors, prolinamide, replicon assay

## Abstract

Hepatitis C virus (HCV) is an international challenge. Since the discovery of NS5A direct-acting antivirals, researchers turned their attention to pursue novel NS5A inhibitors with optimized design and structure. Herein we explore highly potent hepatitis C virus (HCV) NS5A inhibitors; the novel analogs share a common symmetrical prolinamide 2,7-diaminofluorene scaffold. Modification of the 2,7-diaminofluorene backbone included the use of (S)-prolinamide or its isostere (*S,R*)-piperidine-3-caboxamide, both bearing different amino acid residues with terminal carbamate groups. Compound **26** exhibited potent inhibitory activity against HCV genotype (GT) 1b (effective concentration (EC_50_) = 36 pM and a selectivity index of >2.78 × 10^6^). Compound **26** showed high selectivity on GT 1b versus GT 4a. Interestingly, it showed a significant antiviral effect against GT 3a (EC_50_ = 1.2 nM). The structure-activity relationship (SAR) analysis revealed that picomolar inhibitory activity was attained with the use of *S*-prolinamide capped with *R*- isoleucine or *R*-phenylglycine residues bearing a terminal alkyl carbamate group.

## 1. Introduction

Hepatitis C Virus (HCV) infection represents a disease of major global impact that afflicts around 71 million people worldwide. A minor portion of infected HCV cases spontaneously resolve, whereas predominantly patients develop chronic hepatitis C. This further leads to a varying number of complications, ranging from mild inflammation to fibrosis and cirrhosis, conferring significant morbidity and mortality to diseased individuals. The World Health Organization (WHO) evaluates around 200 million individuals to be chronically infected, with 3–4 million new cases prevailing annually. The standard treatment for HCV infection over the past decade was a combinatorial treatment with pegylated interferon alpha (PEG-IFNα) and the antiviral nucleoside analog, ribavirin (RBV). This regimen suffered from many side effects, contraindications, and a low sustained virologic response of 40–60% [1,2,3].

Since 2011, directly acting antivirals (DAAs), including the viral non-structural protein (NS) 3/4A protease inhibitors, the NS5B RNA-dependent RNA polymerase (RdRp) nucleotide-like inhibitors, and NS5A replication complex inhibitors, have received clinical approval for use as part of a combinatorial therapy for hepatitis C and chronic hepatitis C infections [4]. 

Non-structural protein 5A (NS5A), which plays a crucial role in the virus replication, is composed of 447 amino acids, segregated into multiple discrete domains. The first 33 amino acid residues are highly conserved in all genotypes. They form an amphipathic alpha-helix crucial for anchoring NS5A to the recruitment of endoplasmic reticulum membrane and lipid droplets [5]. The rest of the monomer consists of three structural domains (domains I, II, and III), which are separated by low complexity sequences (LCSs) [5,6]. Each NS5A domain binds separately to the RNA 3′-untranslated region (UTR), suggesting that NS5A plays diverse roles in many stages of the virus replication cycle. Reports indicated a role of NS5A during RNA replication and virion assembly in vitro and in vivo. Domain I coordinates a Zn^2+^ binding motif per protein molecule. The zinc atom is coordinated by four NS5A cysteine residues (Cys^39^, Cys^57^, Cys^59^, and Cys^80^), which highlights the importance of this ion in regards to NS5A protein folding, stability, and multiple NS5A related protein functions [6,7]. Domain II (aa 250–342) and III (aa 355–447) are less conserved among HCV genotypes relative to domain I and are natively unfolded [8,9]. Domain II was found to stimulate RNA binding and HCV replication through interaction with the cellular protein cyclophilin A (CyPA) [10]. This confirms the observation that CyPA inhibitors, such as cyclosporine A (CsA), contribute to the inhibition of HCV replication [11]. Domain III is mainly crucial for HCV RNA assembly. This further emphasizes the importance of this protein as a potential drug target [12,13].

The discovery of novel NS5A inhibitors requires the development of several in vitro assays (such as binding, enzymatic, and cell-based assays), in addition to the rational design of targeted inhibitors through enzyme-inhibitor co-crystallization.

The dimeric orientation of NS5A has elicited scientific debates; the crystal structure of the *N*-terminal region showed some remarkable differences. On the other hand, the lack of an inhibitor-protein complex renders a structural-based drug design approach unfeasible.

Moreover, the NS5A protein shows no clear enzymatic function. These challenges intricate the efforts for optimizing the structures of novel NS5A inhibitors. Thus, most of the efforts for the development of NS5A inhibitors are driven by cell-based replicon activities and ligand-based approaches [14]. 

Against all odds, NS5A inhibitors carry unique characteristics that make them attractive candidates for combinatorial therapy. NS5A inhibitors are reported as pan-genotypic with remarkable rapid initial viral RNA decline, while they do not elicit cross-resistance with other direct-acting antivirals (DAAs).

In 2010, daclatasvir (**a**) was discovered and investigated for its ability to inhibit HCV Genotype-1 infection. Daclatasvir was approved by the US FDA in 2014 [15]. Since then, developing new inhibitors that can target NS5A became a hot research topic and an industrial goal [16]. The Chemical structure of the clinical candidate Daclatasvir (**a**) and a highly active reported benzidine prolinamide derivative (**b**) [17], are shown below.


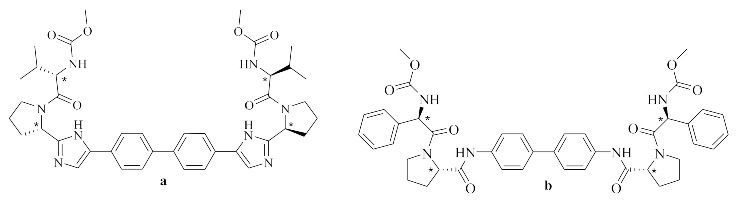


Daclatasvir (**a**) bears a central biphenyl backbone linked to bis-imidazole rings and symmetrical bis-proline moieties; the proline is terminally capped with methylcarbamate *L*-valine. We recently developed novel NS5A inhibitors with a benzidine core and connected *L*-proline as an amide functionality and a variety of capping groups; those compounds lacked the bis-imidazole rings of daclatasvir yet still showed high potency. Compound (**b**) was the most active of this series (EC_50_ = 6.7 pM) [17]. 

**Figure 1:**
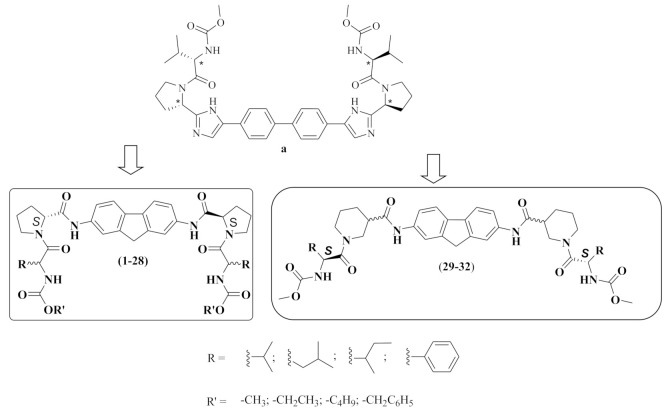
Structures of the novel analogs (**1**–**32**).

We aim to further investigate modifications to symmetrical NS5A inhibitors. Daclatasvir, which is the prototype for them, has been found to be the main NS5A inhibitor in many combination therapies currently found on the market. Thus, here, novel analogs of daclatasvir (**a**) are reported, with significant anti-HCV potency through further rigidification of the core part by changing the benzidine moiety to the tricyclic fluorene ring. Additionally, *L*-proline amide was replaced by proline isostere (*S,R*)-piperidine-3-carboxamide. Several amino acid residues with both *S* and *R* configurations were used, namely: valine, leucine, isoleucine, and phenylglycine. Lastly, the terminal carbamate group was modified to a methyl, ethyl, butyl, or benzyl group (Figure 1).

## 2. Results and Discussion

### 2.1. Chemistry

**Scheme 1:**
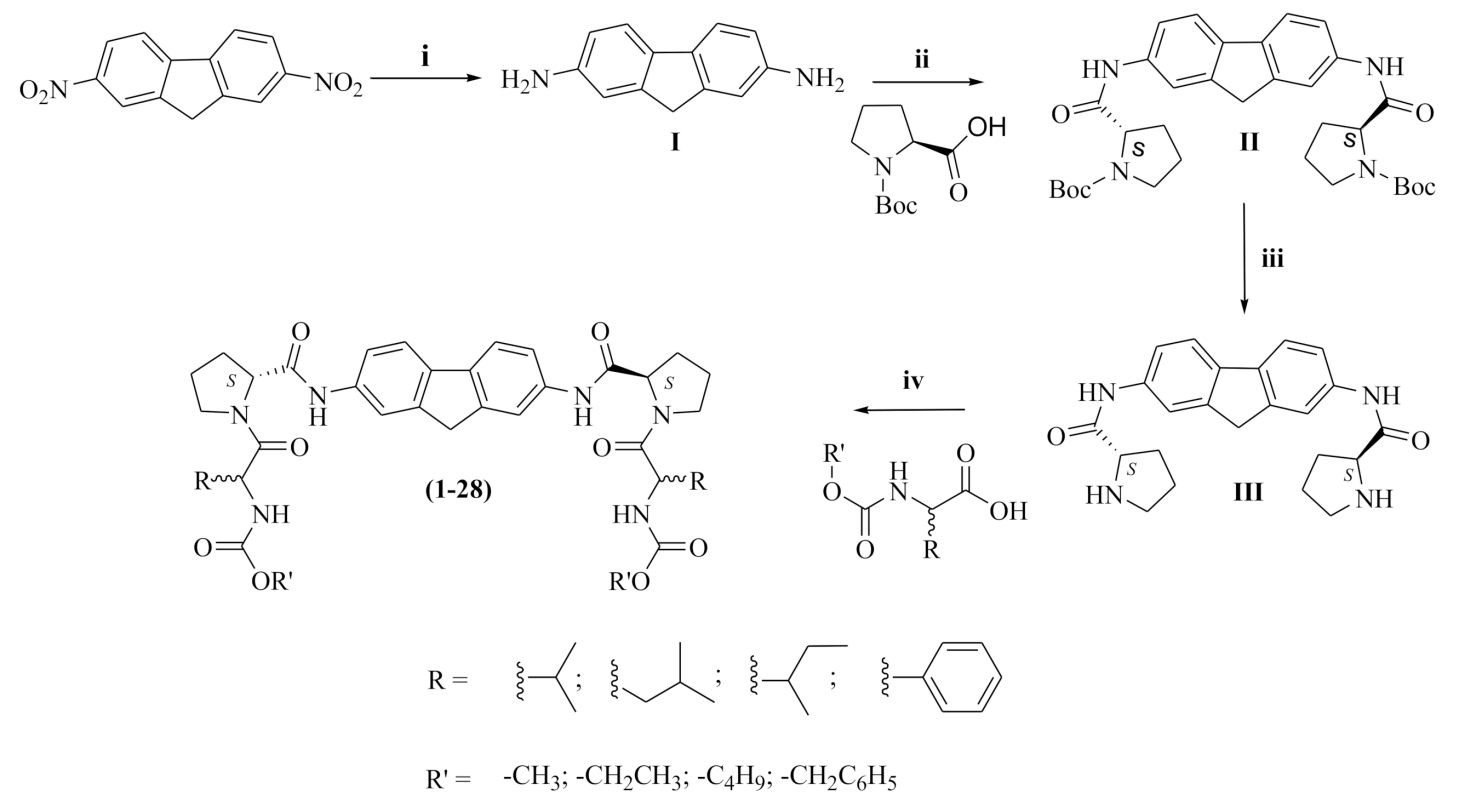
Synthesis of compounds (**1**–**28**). Reagents and conditions: (**i**) SnCl_2_.2H_2_O, HCl/CH_3_COOH, 65 °C, 5 h, yield: 65%; (**ii**) HBTU (1-hydroxy-7-azabenzotriazole), TEA (triethylamine), DMF, room temperature, 30 min, yield: 80%; (**iii**) TFA/CH_2_Cl_2_, 24 h, room temperature, yield: 75%; (**iv**) HBTU, TEA, DMF, 3 h, room temperature, yield: 16–29%.

**Scheme 2:**
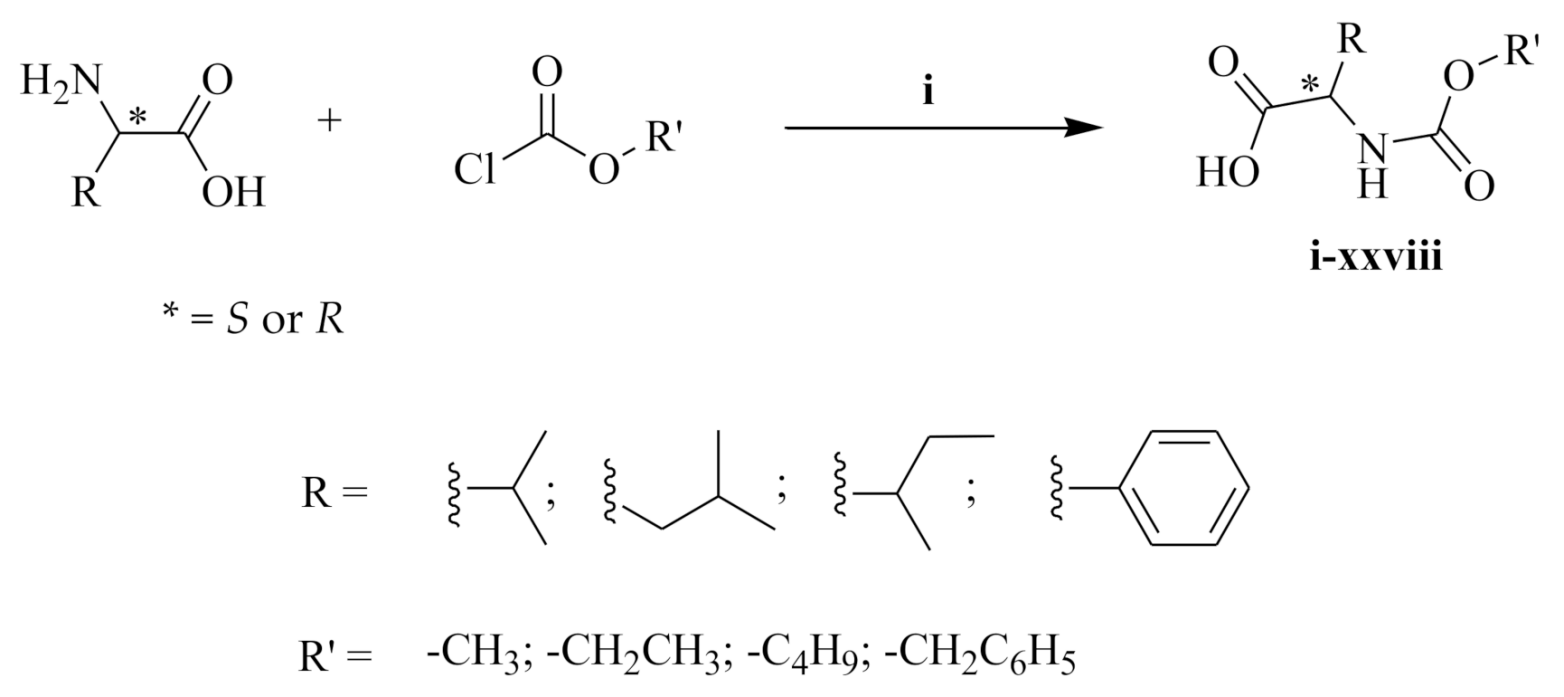
Synthesis of carbamate derivatives (**i**–**xxviii**) [17,20,21] Reagents and conditions**:** (**i**) NaOH in 1,4-dioxane, overnight, room temperature, yield: 48–50%.

Compounds **1**–**28** were prepared using 2,7-dinitrofluorene as a starting material. The nitro groups of 2,7-dinitrofluorene were efficiently reduced by using hydrated stannous chloride in HCl to afford 2,7-diaminofluorene (**I**) at a yield of 65%. Compound **(I)** was coupled with *N*-Boc-*L*-proline (*N*-tert-butyloxycarbonyl-*L*-proline) carboxylic acid in the presence of HBTU/TEA (1-hydroxy-7-azabenzotriazole/triethylamine), which resulted in compound **II** (80% yield) [18]. Deprotection of the latter using TFA (trifluoroacetic acid) provided compound **III** (75% yield). Amino acid carbamate derivatives (**i–xxviii**) were coupled with compound **III** using HBTU in the presence of TEA in dimethylformamide (DMF) as a solvent for 3 h at room temperature to yield compounds **1–28** (Scheme 1). Amino acid carbamate derivatives (**i-xxviii**) were synthesized by reacting commercially available amino acids with various chloroformates using reported procedures (Scheme 2) [19]. The exact stereochemical assignment and substituents are shown in Table 1.

**Scheme 3:**
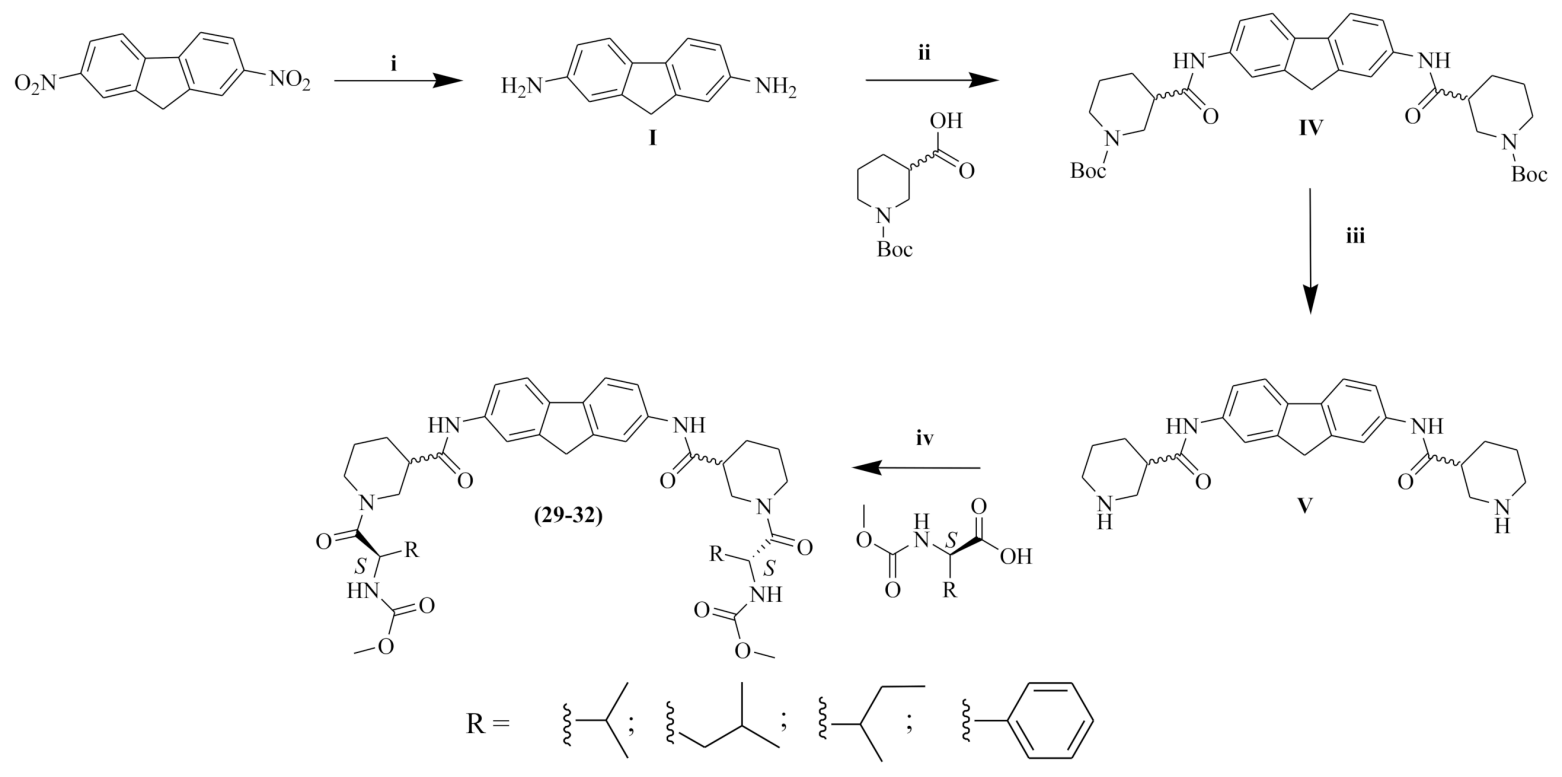
Synthesis of compounds (**29–32**). Reagents and conditions: (**i**) SnCl_2_.2H_2_O, HCl/CH_3_COOH, 65 °C, 5 h, yield: 65%; (**ii**) HBTU, TEA, DMF, room temperature, 30 min, yield: 41%; (**iii**) TFA/CH_2_Cl_2_, 24 h, room temperature, yield: 51%; (**iv**) HBTU, TEA, DMF, 3 h, room temperature, yield: 15–18%. For details of compounds characterization, see the experimental section.

The second series was synthesized by reacting compound **I** with *N*-Boc-(*S*,*R*)-piperidine-3-carboxylic acid to yield compound **IV** (41% yield), which was then deprotected to give compound **V** (51% yield). The latter was coupled with methylcarbamate of different amino acids utilizing the same coupling procedures to yield compounds **29**–**32**. (Scheme 3) [19]. The exact stereochemical assignment and substituents are shown in Table 2.

### 2.2. Biological Studies

All the synthesized compounds were evaluated for their anti-HCV 1b and their cytotoxicity, from which the selectivity index (SI_50_) was calculated. The most active compound was tested for its antiviral activity on other genotypes and possible interactions with daclatasvir, and the coefficient of drug interaction was calculated. In addition, the effect of the most active compound was tested for its effects upon the expression of the viral RNA and NS5A proteins by qPCR and Western blot analysis, respectively. For the exact methodologies, see the experimental section.

#### 2.2.1. Cytotoxicity and Anti-HCV 1b Activity of the Synthesized Compounds 

The HCV RNA replication inhibitory effect of the novel compounds and their impact on cell viability was tested in the stable cell line Huh5-2 carrying an HCV subgenomic reporter replicon of genotype 1b (strain Con1) [22]. The viral replication level was measured in terms of the activity of the co-expressed firefly luciferase (F-Luc). Several concentrations of the tested compounds were prepared in a cell culture medium and were used to treat the cells for 72 h. The solvent DMSO was added as a control with a final concentration of 0.2% so as not to impact viral replication (data not shown). First, different compound concentrations of up to 100 μM were evaluated for their effect on cell viability; results are reported as the median cytotoxic concentration (CC_50_). Next, all compounds were screened for their anti-HCV activity at a concentration of 1 µM, and compounds showing ≥50% activity were further tested in several concentrations to calculate the median effective concentration (EC_50_) values (Table 1 and Table 2). The anti-HCV activity of the analogs **1**–**32** was estimated by quantifying luciferase activity resulting from viral replication. Cytotoxicity was determined by measuring the intracellular levels of ATP. The selectivity index (SI_50_) was calculated as the ratio of CC_50_ to EC_50_ and was calculated as an indication of the compounds’ safety_._ We used daclatasvir as a positive control.

The effect of the three major structural modifications on activity was investigated. First, we turned our attention to the effect of the nature of the lipophilic amino acid in the capping group, which includes size, chirality, branching and aromaticity. Second, we evaluated the impact of changing the nature of the *O-* substituents of the terminal amino acid capping group, which includes size and aromaticity, Table 1. 

Finally, the effect of bioisoster replacement of *L-* proline with (*S, R*) 1,3-disubstituted piperidine was tested, Table 2. We compared the fluorene to the benzidine core using a congener previously reported by our research group (b**)** [17]**.**

Both *D-* and *L-*epimers of valine, leucine, phenylglycine, whereas only *L-*isoleucine, were employed as an amino acid residue of the capping group. The use of both natural and unnatural amino acids can expand the pool of building blocks of NS5A inhibitors; additionally, the use of unnatural amino acids can have an impact on compound stability and resistance development. 

For the valine residues, the *R* congeners were more active than their *S* epimers except for compound **1**. For the leucine residues, compound **9** was around 340 times less active than its *R* congener compound **13**. On the contrary, compound **10** bearing an *L-*leucine was 18-fold more potent than its congener that has *D-*leucine moiety. For phenylglycine residues, compounds **25**, **26** were 60-fold and 200-fold more active than their *S* counterparts **21**, **22**, respectively. Analog **25** was 60-fold more potent than **21**. The opposite was observed comparing compound **28** and its *R* counterpart **24**. It is worth mentioning that three out of the four compounds showing picomolar activity have an *R* amino acid residue, indicating that the interaction with the receptor is not stereo-specific.

It seems that the *R* configuration is generally preferred for compounds bearing a fluorene prolinamide core, even though most of the NSSA inhibitors discovered incorporated an *S*-valine residue. This expands the pool of amino acids that can be adopted in the design of new NS5A inhibitors.

Next, we extended our investigation to what effect the size and aromaticity of the amino acid residue of the capping group have on anti-HCV activity. It was found that the largest set of active analogs (**21**–**28**) bore a phenylglycine residue indicating that activity improves with aromatic amino acid residues. Τhis can be attributed to both the lipophilicity of these residues and their ability to be involved in van der Waals or π-π stacking interactions. Both *D*- and *L*-leucine compounds (**9**–**16**) exhibited better activity when compared to their valine congeners (**1**–**8**), indicating that more lipophilic residues are favored. This could encourage the preparation of commercial NS5A inhibitors from aromatic amino acid residues rather than the commonly adopted *S-*valine. 

The effect of the substituent on the terminal oxygen of the capping group was also investigated. Results showed that the terminal capping group was not a detrimental factor of activity compared to the amino acid residue’s chirality and lipophilicity. There was no clear correlation between these groups and activity, yet we have generally noticed that a methyl or a benzyl substituent positively affects the activity. Out of the nine most active analogs, four compounds bore a methyl or benzyl terminal substituent (**13**, **21**, **24,** and **25**). 

Comparing compound **26**, the most active analog bearing an *R*-phenylglycine amino acid residue with a terminal ethyl carbamate to its counterparts **25**, **27,** and **28**, we noticed that compound **25** bearing a terminal methyl substituent was four times less potent, while compound **27** bearing a butyl terminal group, showed 100 times less potency. A terminal benzyl group reduced the activity by 2363 times. The introduction of two extra benzyl groups increased the clog P from 5.7 in the case of methyl substituent to 7.4, which may have affected both the ability of the compound to appropriately penetrate hepatoma cells and its solubility during the assay and may have led to false lower potency. 

The lack of a clear correlation pattern between terminal *O*-substituent and antiviral activity is observed; we noticed in some cases that an aromatic lipophilic substituent is favored compared to a small methyl substituent for compounds **8** and **24** compared to compounds **5** and **21.** These findings indicate that the nature and conformation of the entirety of the compound are crucial for activity rather than a single substitution.

We further investigated the optimal stereoelectronic features by replacing the commonly adopted *L-*proline with a six-membered common proline-memetic, namely (*S,R*) piperidine. The latter was coupled at position three to the fluorene core with an amide linkage. The terminal *N*-substituent was kept as methyl, and four *S* amino acid residues were studied, namely valine, leucine, phenyl glycine, and isoleucine. 

All compounds were less active than their congeners bearing *L-*proline, indicating that a five-membered heterocycle enhances activity compared to a six-membered congener. Compounds **1**, **9**, **17,** and **21** were found to be five, 11, 28, and 36-fold more potent than their six-membered congeners **29**, **30**, **31,** and **32,** respectively.

The racemic (*S,R*) center might have contributed negatively to the activity, compared to a pure *S* or *R* chiral center. This can further be supported by the previously published work of our group, where compounds bearing the *D-*proline were found to be inactive relative to their *L-*proline congeners [20]. More research should be directed towards the preparation of novel analogs with pure enantiomers of piperidine carboxylic acid and its derivatives.

Compound **26**, the most active analog of this series, bore an *R-*phenylglycine moiety and ethyl carbamate terminal moiety.

**Figure 2:**
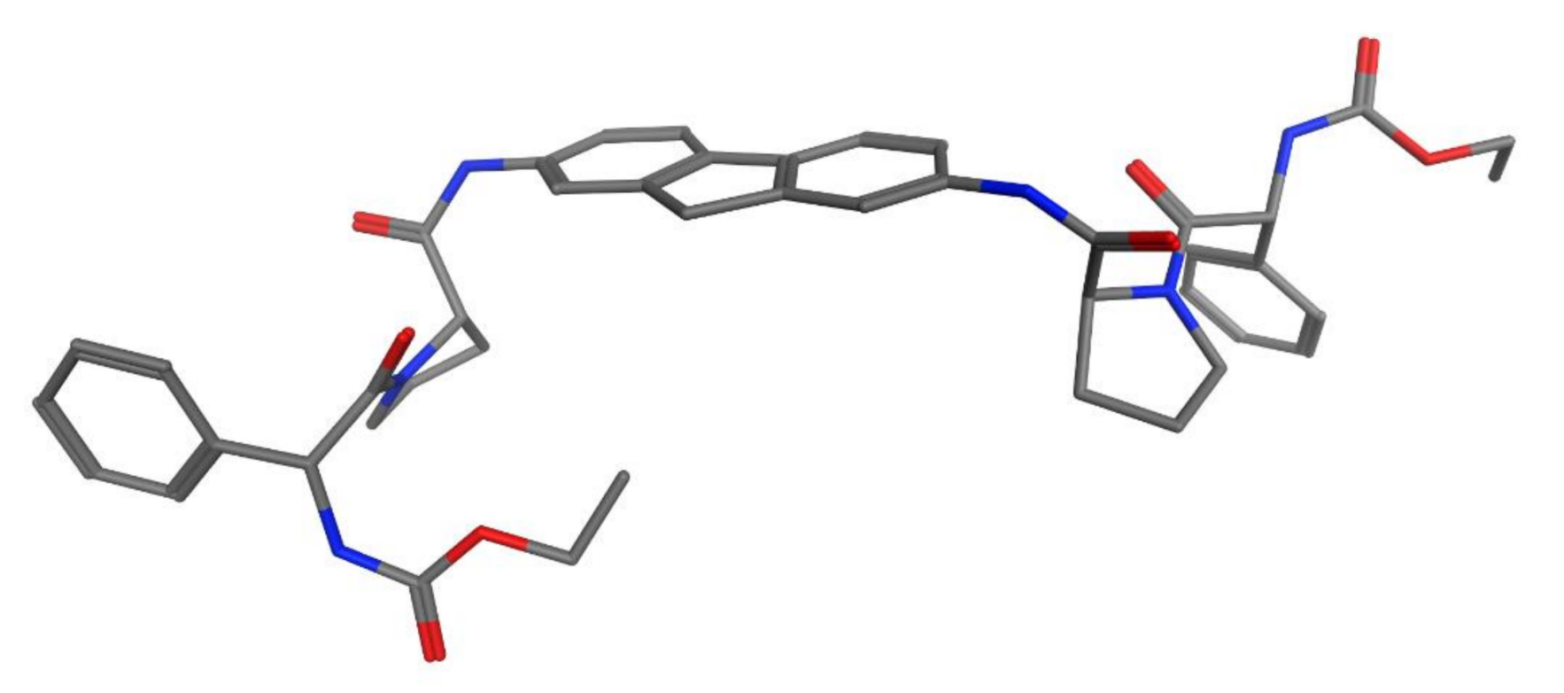
A planar fluorene core in compound **26** from which the two symmetric capping groups are projected.

Energy minimization of compound **26** (EC_50_ = 40 pM) using Chem3D ultra 7.0.0. revealed a planar conformation of the tricyclic fused fluorene core, while the terminal two ethyl moieties projected in the opposite direction, which may indicate that each of them enters in one of the two chains of the NS5A protein (Figure 2).

**Figure 3:**
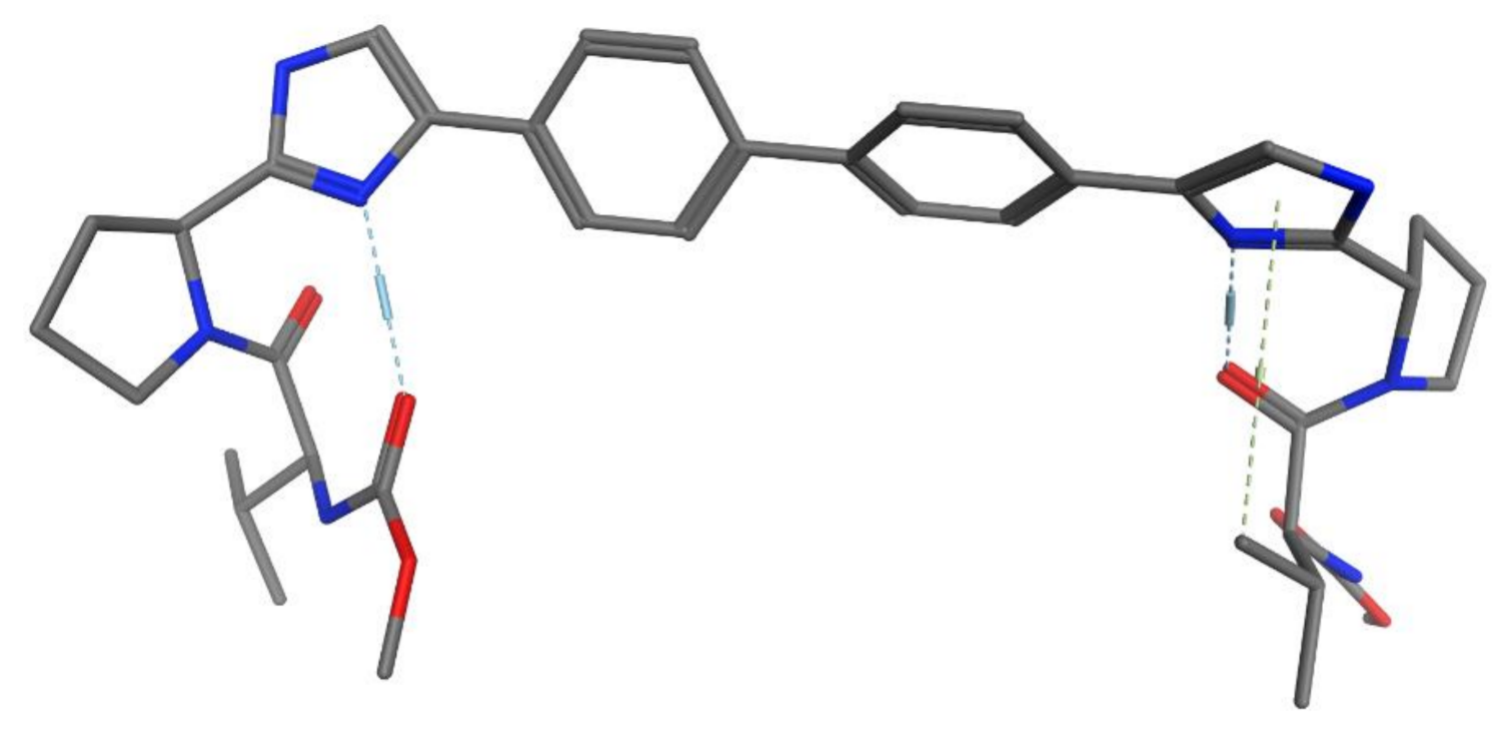
A non-coplanar benzidine core with a dihyderal angle of 138.04°.

To assess the effect of compound core planarity on activity, compound **b** (EC_50_ = 6.7 pM) was energy minimized; the two phenyl cores showed non-coplanarity and a dihyderal angle of 138.04°. It seems that planarity is not favored for NS5A binding (Figure 3) [17]. 

Based on the CC_50_ and SI_50_ values presented in Table 1 and Table 2, all analogs (**1**–**32**) showed low cytotoxicity, even lower than daclatasvir (CC_50_ = 17.7 μM) and high selectivity against virus replication. The most potent of them, compound **26**, showed the highest selectivity index (SI_50_ > 2779322), four-fold better than that of daclatasvir (SI_50_ = 655,556), which indicates the very high safety profile. These promising results encourage adopting this scaffold in the future for the development of NS5A inhibitors. 

Finally, intermediate compound **IV** bearing a tertiary *N*-butoxy carbamate on the piperidine amide moiety was tested, as a negative control, for its antiviral activity. Results indicate the pivotal role of the terminal amino acid residue capped with a substituted carbamate.

#### 2.2.2. Activity of the Most Active Compound 26 Against HCV Genotypes 3a and 4a

Compound **26**, showing the highest activity on genotype 1b was further tested against HCV 3a and 4a replicons. The fact that genotype 4 is highly heterogeneous, of less response to DAAs, and the most prevalent genotype in Egypt, made this genotype of specific interest. Moreover, genotype 3 is the most difficult to treat genotype using DAAs, especially in patients with liver cirrhosis [23]. Specifically, Huh7.5-3a and Huh7.5-4a stable cell lines having HCV subgenomic replicons of genotypes 3a (strain S52) and 4a (strain ED43), respectively, were treated with different concentrations of compound **26**. Interestingly, a significant effect was observed on GT 3a (EC_50_ = 1.2 nM), which was about seven-fold higher than the one exerted by daclatasvir (EC_50_ = 8.3 nM). There was no significant activity observed for GT 4a (EC_50_ = 117 nM). Further structural optimization is required, and testing compound **26** in combination with other DAAs could improve the clinical outcome.

#### 2.2.3. Drug-Drug Interaction Studies of Compound 26 with the Approved HCV Inhibitor Daclatasvir

**Figure 4:**
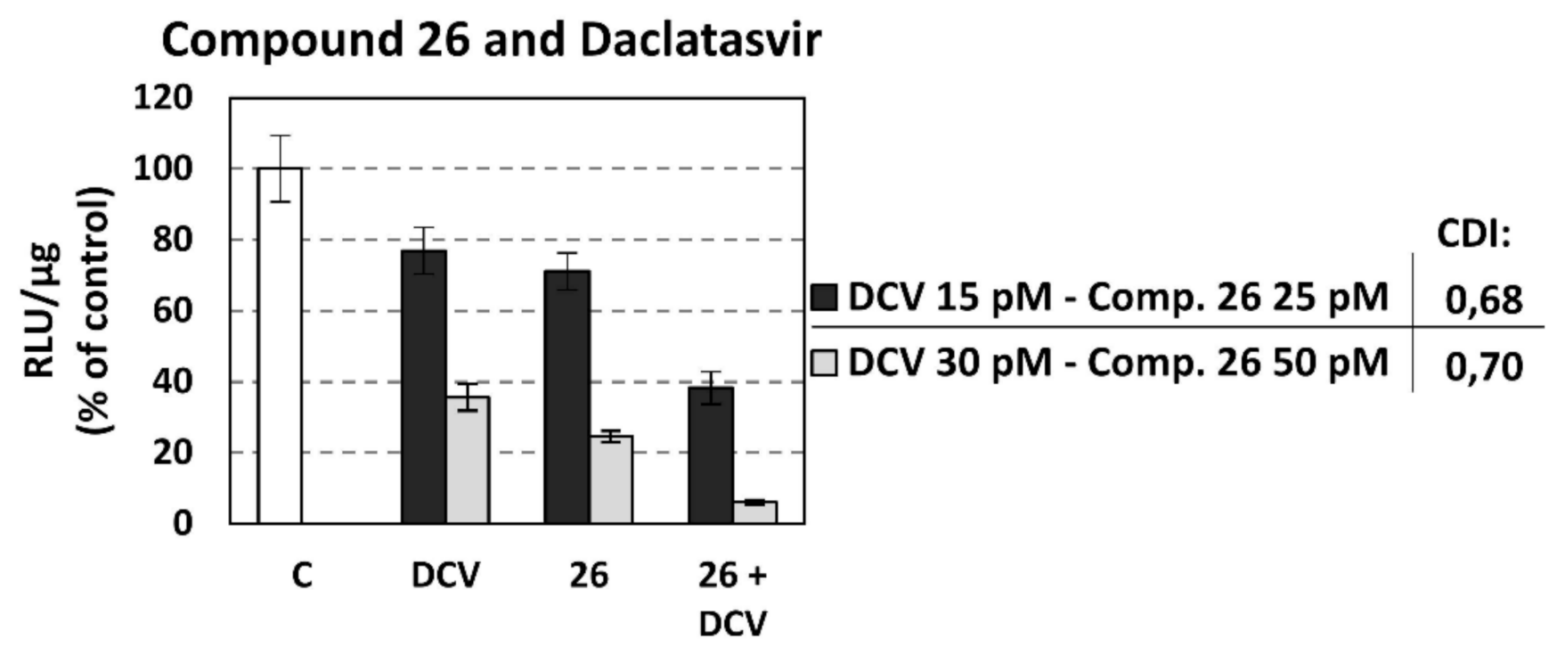
Synergistic anti-HCV activity of compound **26** with daclatasvir (DCV). Huh5-2 cells were treated with 30 pM or 10 pM of DCV was used to in the presence or absence of the analog. F-Luc activity was quantified as relative light units (RLU) per μg of total protein. Values derived from cells treated with the solvent DMSO (control—C) were set to 100%. CDI: coefficient of drug interaction. Bars show mean values derived from three independent experiments in triplicate. Error bars denote standard deviation (SD).

Next, we investigated the effect of compound **26** in the presence of daclatasvir (DCV) in Huh5-2 replicon cells. As shown in Figure 4, compound **26** and DCV had a significant synergistic effect. More specifically, compound **26** showed a coefficient of drug interaction (CDI ≈ 0.7). It is worth noting that NS5A is a very flexible protein and can attain different conformations (open conformer and closed conformer PDB ID: 1ZH1, 3FQM, respectively); thus, there is the potential for different binding modes of daclatasvir and our molecules, at the active center of the NS5A protein, leading to synergism. [24]

This is supported by a previous manuscript that shows the synergistic effect between the NS5A inhibitor daclatasvir and other NS5A inhibitors [25]. 

The coefficient of Drug interactions (CDI) is calculated as follows: CDI = AB / (A × B), where AB is the ratio of the combination groups to the control group; A or B is the ratio of the single-agent group to the control group. Thus, CDI value <1, = 1, or >1 indicates that the drugs are synergistic, additive, or antagonistic, respectively.

#### 2.2.4. Validation of Compound Activity with Additional Assays 

**Figure 5:**
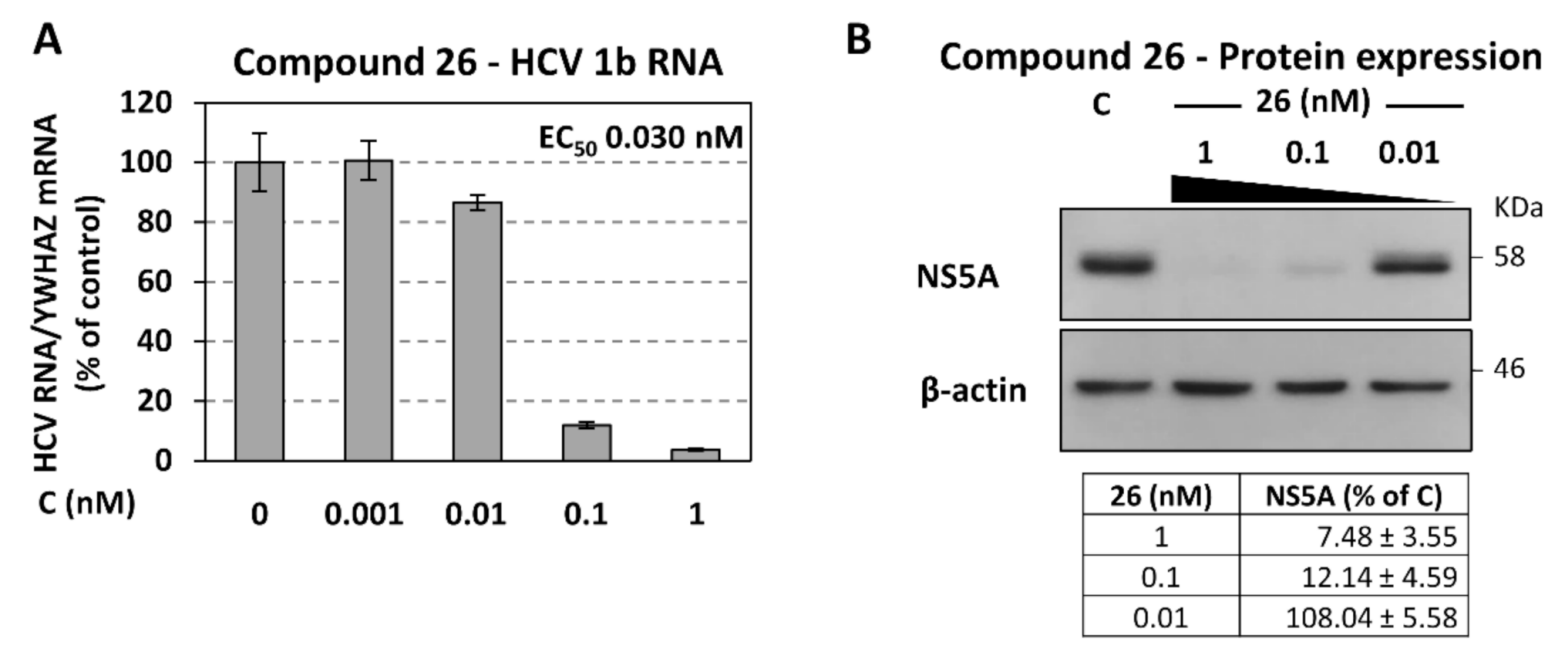
Anti-HCV activity of compound **26** at the level of viral RNA and protein. Different concentrations of compound **26,** or the solvent DMSO (control—C), were used to treat Huh5-2 cells. (**A**) Quantification of (+) strand HCV RNA by RT-qPCR. Values are expressed as a percentage of those obtained from control cells. mRNA levels of the housekeeping gene 14-3-3-zeta polypeptide (YWHAZ) were used for normalization. (**B**) (Top) Western blot analysis for NS5A. β-actin was used as a loading control. Numbers on the right refer to the positions of molecular mass marker proteins. A representative experiment of three independent repetitions is shown. (Bottom) Densitometry analysis data for NS5A (mean values from three independent repetitions) were normalized to β-actin and to the values obtained with control cells (Figure S1).

The HCV RNA and NS5A protein levels were determined in cells treated with compound **26** using reverse transcription-quantitative polymerase chain reaction (RT-qPCR) or Western blot analysis, respectively (Figure 5A).

Compound **26** inhibited HCV RNA replication, with EC_50_ similar to that calculated by luciferase assay (Table 1). Consistently, NS5A levels were reduced after treatment with the inhibitor (Figure 5B).

## 3. Material and Methods 

### 3.1. Chemistry

Solvents and reagents were obtained from commercial suppliers and were used without further purification. All solvents used were of pure analytical grade. Reactions that needed anhydrous conditions were carried out in flame-dried glassware under a positive pressure of dry N_2_ using the Schlenk line technique. Column chromatography was carried out using silica gel 40–60 μM. Reaction progress was monitored by TLC using fluorescent pre-coated silica gel plates, and detection of the components was made by short UV light (λ = 254 nm); carbamates were detected using freshly prepared furfural/sulfuric acid detection reagent. Proton nuclear magnetic resonance (^1^H-NMR spectra and ^13^C-NMR spectra) were recorded using a Bruker Fourier 300 using Varian Mercury 400 Plus. All spectra were determined in the solvents indicated, and chemical shifts were reported in δ units. Solvent signals were δ 2.50 for Dimethyl sulfoxide (DMSO-d6) and δ7.2 for Chloroform (CDCl_3_). ^13^C shifts were referenced to the deuterated solvent signal δ 39.5 for DMSO-d6 and δ 77.0 for CDCl_3_. Coupling constants (J) are given in hertz (Hz). Multiplicities patterns were as follows: s: singlet; d: doublet; t: triplet; q: quartet; m: multiplet; dd:doublet of doublet; ddd: doublet of doublet of doublet. All NMR spectra were analyzed using MestReNova software version 6.0.2–5475. Mass spectrometric analysis (UPLC-ESI-MS) was performed using Waters ACQUITY Xevo TQD system, which consisted of an ACQUITY UPLC H-Class system and XevoTM TQD triple-quadrupole tandem mass spectrometer with an electrospray ionization (ESI) interface (Waters Corp., Milford, MA, USA). Acquity BEH C18 100 mm × 2.1 mm column (particle size, 1.7 µm) was used to separate analytes (Waters, Ireland). The solvent system consisted of water containing 0.1% TFA (A) and 0.1% TFA in acetonitrile (B). HPLC-method: flow rate 200 μL/min. The percentage of B started at an initial of 5% and maintained for 1 minute, then increased up to 100% during 10 min, kept at 100% for 2 min, and flushed back to 5% in 3 min then kept at 5% for 1 min. The MS scan was carried out at the following conditions: capillary voltage 3.5 kV, cone voltage 20 V, radio frequency (RF) lens voltage 2.5 V, source temperature 150 °C, and desolvation gas temperature 500 °C. Nitrogen was used as the desolvation and cone gas at a flow rate of 1000 and 20 L/h, respectively. System operation and data acquisition were controlled using Mass Lynx 4.1 software (Waters). The purities of the tested compounds were determined by HPLC coupled with mass spectrometry and were higher than 95% purity unless otherwise stated (purity of compound **18** was >90%). Measurements for a melting point were not changed and were done using capillary Büchi B-540 melting point apparatus.

#### 3.1.1. General Procedure for Carbamates Synthesis (**i**–**xxviii**)

In a 250 mL round bottom flask equipped with a magnetic stirring bar, 25 mL of 1M NaOH was cooled in an ice bath to 0 °C. The respective amino acid was added (8 mmol), and the solution was stirred until it became completely homogenous. The corresponding chloroformate (11 mmol), dissolved in 1,4-dioxane (10 mL), was added dropwise. The reaction mixture was left to stir overnight at room temperature. The reaction mixture was washed with diethyl ether (3 × 50 mL), the organic layer was discarded. The aqueous layer is placed in an ice bath and acidified dropwise to pH = 2 using concentrated HCl. The respective carbamate is extracted from the aqueous layer by adding diethyl ether (3 × 50 mL). Organic layers were combined, dried over anhydrous Na_2_SO_4,_ and concentrated under vacuum to give the crude product either as a clear, viscous oil or as a white solid. No further purification was required (Scheme 2) [17,20,21] .

#### 3.1.2. General Procedure for the Preparation of 2,7-diaminofluorene (**I**)

The starting material of 2,7-dinitrofluorene (4 mmol, 1 g) was placed into a 250 mL round-bottom flask together with SnCl_2_.2H_2_O (80 mmol, 16 g) in concentrated HCl (12 mL) using acetic acid (24 mL) as a solvent. The mixture was refluxed for 5 h at 65 °C. The resulting suspension was cooled and poured into an aqueous solution of (313 mmol, 12.5 g) of NaOH in 500 mL of water. The precipitate was filtered, excess NaOH was added to pH > 7 and extracted using diethyl ether (3 × 25 mL). Organic layers were combined, dried over anhydrous Na_2_SO_4,_ and concentrated under vacuum. The crude product was purified by silica gel column chromatography using a mixture of methylene chloride: methanol (10:0.3) and dried under vacuum to provide a light brown solid compound **I** (0.65 g, 65%). Compound **I** appeared as a distinct blue spot using thin liquid chromatography [26].

#### 3.1.3. General Procedure for Preparation of Compounds **II** and **IV**

Compound **I** (1 mmol, 0.5 g) was coupled with either *N-*Boc-*L-*proline or *N*-Boc-(*S,R*)-piperidine-3-carboxylic acid (2 mmol) using HBTU (1 mmol, 0.967 g). Compound **I** was added in one portion to the *N*-Boc derivative and TEA (2 mmol, 0.710 mL). The mixture was stirred for 15 min in DMF in an ice bath. The reaction mixture was left to stir at room temperature for 30 min. DMF was removed under vacuum, and the residue was extracted using DCM and saturated sodium chloride solution. Organic layers were combined, dried over anhydrous Na_2_SO_4,_ and concentrated under vacuum to yield a dark red/brown oily product compound **II** (80%) and dark red oily product compound **IV** (41%) [26].

#### 3.1.4. General Procedure for Preparation of Compounds **III** and **V**

Compound **II** was dissolved in DCM (40 mL) and treated with TFA (6 mL) at room temperature under an N_2_ atmosphere. The reaction was left to stir at room temperature for 24 h then both TFA and DCM were evaporated under vacuum to yield compounds **III** and **V.**

The reaction residue was dissolved in EtOAc, then 1M NaOH was added gradually till pH = 12. The mixture was extracted using EtOAc (3 × 25 mL). Organic layers were combined, dried over Na_2_SO_4_, and concentrated under vacuum. No further purification was required [26].

#### 3.1.5. General Procedure for Preparation of Compounds (**1**–**32**)

HBTU (1 mmol, 1.554 gm) was added in one portion to the respective amino acid carbamate (3 mmol) and TEA (2 mmol, 0.571 mL), the mixture was stirred for 15 min in DMF under ice-cooling. Compounds **III** and **V** (1 mmol) were added portion-wise to the above mixture. The reaction mixture was left to stir at room temperature for 3 h. The crude product was purified by silica gel column chromatography using a mixture of DCM: MeOH.

***Di-tert-butyl3,3’-(((9H-fluorene-2,7diyl)bis(azanediyl))bis(carbonyl))bis(piperidine-1-carboxylate) (IV)***

Dark red Oil; Yield 41%; ^1^H NMR (400 MHz, DMSO-*d6*) δ 9.77 (s, 2H), 7.61 (s, 2H), 7.44 (d, J = 8.2 Hz, 2H), 7.26 (d, J = 8.2 Hz, 2H), 3.69 (d, J = 48.0 Hz, 4H), 3.59 (s, 2H), 2.22 (d, J = 13.7 Hz, 4H), 2.19 (d, J = 10.9 Hz, 2H), 1.72–1.44 (m, 4H), 1.34 (dd, J = 23.9, 12.2 Hz, 4H), 1.14 (s, 18H); ^13^C NMR (101 MHz, DMSO-*d6*) δ 172.19, 154.34, 143.95, 138.05, 136.74, 119.94, 118.39, 116.45, 85.18, 60.69, 59.11, 37.04, 28.04, 24.68, 24.54, and 22.89; MS (ESI) m/z: 619 [M + H] ^+^.

***N, N’-(9H-fluorene-2,7-diyl)bis(piperidine-3-carboxamide) (V)***

Dark red oil; Yield 51%; ^1^H NMR (400 MHz, DMSO-*d6*) δ 8.24 (s,2H), 7.91 (s, 2H), 7.69 (d, J = 8.4 Hz, 2H), 7.54–7.52 (m, 2H), 3.48 (s, 2H), 2.79 (d, J = 2.6 Hz, 4H), 2.71–2.69 (m, 4H), 2.58–2.55 (m, 2H), 1.90–1.85 (m, 4H), 1.71–1.67 (m, 2H), 1.48–1.41 (m, 4H); ^13^C NMR (101 MHz, DMSO-*d6*) δ 159.46, 125.55, 122.08, 118.91, 115.91, 115.32, 112.95, 63.05, 60.43, 31.17, 24.24, 22.44, and 21.60; MS (ESI) m/z: 419 [M+ H] ^+^.

***Dimethyl((2S,2’S)-((2S,2’S)-(((9H-fluorene-2,7-diyl)bis(azanediyl))bis(carbonyl)) bis(pyrrolidine-2,1-diyl))bis(3-methyl-1-oxobutane-1,2-diyl))dicarbamate (1)***

Yellow oil; Yield 20.6%; ^1^H NMR (500 MHz, DMSO-*d6*) δ 10.07 (s, 2H), 7.89 (s, 2H), 7.72–7.69 (m, 2H), 7.47 (d, J = 8.2 Hz, 2H), 7.33 (d, J = 8.4 Hz, 2H), 4.05 (t, J = 8.5 Hz, 2H), 3.86 (s, 2H), 3.66–3.61 (m, 2H), 3.53 (s, 6H), 2.19–2.13 (m, 6H), 1.97–1.87 (m, 8H), 0.90 (d, J = 6.6 Hz, 12H); ^13^C NMR (126 MHz, DMSO-*d6*) δ 170.85, 170.73, 157.26, 144.18, 138.11, 136.64, 119.96, 118.21, 116.28, 60.69, 58.43, 51.89, 47.71, 31.10, 29.98, 25.12, 19.39, and 19.12; MS (ESI) m/z: 705.35 [M+ H]^+^.

***Diethyl((2S,2’S)-((2S,2’S)-(((9H-fluorene-2,7-diyl)bis(azanediyl))bis(carbonyl)) bis(pyrrolidine-2,1-diyl))bis(3-methyl-1-oxobutane-1,2-diyl))dicarbamate (2)***

Yellow oil ; Yield 20%; ^1^H NMR (500 MHz, DMSO-d6) δ 10.06 (s, 2H), 7.89 (s, 2H), 7.70 (d, *J* = 8.3 Hz, 2H), 7.49–7.46 (m, 2H), 7.23 (d, *J* = 8.3 Hz, 2H), 4.48 (dd, *J* = 8.1, 4.8 Hz, 2H), 4.04 (t, *J* = 8.4 Hz, 2H), 4.01–3.97 (m, 4H), 3.87–3.80 (m, 4H), 3.68–3.60 (m, 2H), 2.18–2.12 (m, 2H), 1.97–1.88 (m, 8H), 1.16 (t, *J* = 7.1 Hz, 6H), 0.93 (dd, *J* = 31.3, 6.7 Hz, 12H); ^13^C NMR (126 MHz, DMSO-*d6*) δ 170.88, 170.75, 156.98, 144.02, 138.11, 136.64, 119.96, 118.21, 116.28, 60.70, 60.28, 58.33, 47.69, 38.71, 31.16, 25.14, 19.41, 19.11, and 15.11; MS (ESI) m/z: 733.38 [M+ H]^+^.

***Dibutyl((2S,2’S)-((2S,2’S)-(((9H-fluorene-2,7-diyl)bis(azanediyl))bis(carbonyl))bis(pyrrolidine-2,1-diyl))bis(3-methyl-1-oxobutane-1,2-diyl))dicarbamate (3)***

Yellow oil; Yield 23.5%; ^1^H NMR (500 MHz, DMSO-*d6*) δ 10.05 (s, 2H), 7.89 (s, 2H), 7.70 (d, J = 8.3 Hz, 2H), 7.48 (d, J = 8.2 Hz, 2H), 7.21 (d, J = 8.3 Hz, 2H), 4.48 (dd, J = 8.0, 4.5 Hz, 2H), 4.05 (t, J = 8.4 Hz, 2H), 3.93 (dt, J = 10.7, 5.4 Hz, 4H), 3.86 (s, 2H), 3.82 (d, J = 9.0 Hz, 2H), 3.64 (dd, J = 15.7, 6.3 Hz, 2H), 2.17 (dd, J = 12.8, 8.1 Hz, 2H), 2.00–1.85 (m, 8H), 1.56–1.47 (m, 4H), 1.35–1.28 (m, 4H), 0.96 (d, J = 6.6 Hz, 6H), 0.89 (t, J = 7.4 Hz, 12H); ^13^C NMR (101 MHz, DMSO-*d6*) δ 170.87, 170.73, 156.94, 144.01, 138.10, 136.65, 119.94, 118.23, 116.29, 64.09, 58.34, 55.36, 47.70, 38.70, 31.21, 31.13, 29.94, 25.14, 19.40, 19.07, and 14.07; MS (ESI) m/z: 789.45 [M+ H]^+^.

***Dibenzyl ((2S,2’S)-((2S,2’S)-(((9H-fluorene-2,7- diyl)bis(azanediyl))bis(carbonyl)) bis(pyrrolidine-2,1-diyl))bis(3-methyl-1-oxobutane-1,2-diyl))dicarbamate (4)***

Yellow oil; Yield 26%; ^1^H NMR (500 MHz, DMSO-*d6*) δ 10(s, 2H), 7.89 (s, 2H), 7.70 (d, J = 8.3 Hz, 2H), 7.46 (t, J = 8.4 Hz, 2H), 7.35 (s, 10H), 7.32 (dd, J = 5.6, 3.4 Hz, 2H), 5.03 (d, J = 8.0 Hz, 4H), 4.51–4.44 (m, 2H), 4.08 (t, J = 8.4 Hz, 2H), 3.87–3.82 (m, 4H), 3.63 (dd, J = 15.3, 6 Hz, 2H), 2.21–2.17 (m, 2H), 2.02–1.89 (m, 8H), 0.97 (d, J = 6.7 Hz, 12H); ^13^C NMR (101 MHz, DMSO-*d6*) δ 170.74, 170.73, 165.06, 156.70, 144.01, 138.09, 137.56, 136.65, 128.80, 128.11, 119.94, 118.23, 116.30, 65.86, 60.71, 58.46, 47.69, 38.70, 31.14, 25.11, 19.41, and 19.05; MS (ESI) m/z: 857.42 [M+ H]^+^. 

***Dimethyl ((2R,2’R)-((2S,2’S)-(((9H-fluorene-2,7-diyl)bis(azanediyl))bis(carbonyl)) bis(pyrrolidine-2,1-diyl))bis(3-methyl-1-oxobutane-1,2-diyl))dicarbamate (5)***

Yellow oil; Yield 19%; ^1^H NMR (500 MHz, DMSO-*d6*) δ 9.72 (s, 2H), 7.88 (s, 2H), 7.71 (d, J = 8.2 Hz, 2H), 7.54 (d, J = 7.5 Hz, 2H), 7.39 (d, J = 8.1 Hz, 2H), 4.49–4.42 (m, 2H), 4.10 (t, J = 8.1 Hz, 2H), 3.85 (t, J = 10.4 Hz, 4H), 3.57 (s, 6H), 3.52–3.42 (m, 2H), 2.16–2.10 (m, 4H), 1.98–1.92 (m, 4H), 1.45 (s, 2H), 0.93–0.86 (m, 12H); ^13^C NMR (126 MHz, DMSO-*d6*) δ 172.26, 170.79, 157.51, 143.95, 136.77, 134.06, 119.97, 118.34, 116.48, 60.82, 58.54, 52.06, 47.54, 30.16, 27.32, 24.82, 19.50, and 18.84; MS (ESI) m/z: 705.35 [M+ H]^+^.

***Diethyl((2R,2’R)-((2S,2’S)-(((9H-fluorene-2,7-diyl)bis(azanediyl))bis(carbonyl)) bis(pyrrolidine-2,1-diyl))bis(3-methyl-1-oxobutane-1,2-diyl))dicarbamate (6)***

Yellow oil ; yield 24.3%; ^1^H NMR (500 MHz, DMSO-*d6*) δ 9.67 (s, 2H), 7.88 (s, 2H), 7.71 (d, J = 6.2 Hz, 2H), 7.55 (d, J = 8.2 Hz, 2H), 7.31 (d, J = 7.9 Hz, 2H), 4.45 (d, J = 6.5 Hz, 2H), 4.02–3.98 (m, 4H), 3.85 (s, 2H), 3.62 (dd, J = 16.6, 7.5 Hz, 2H), 3.00–2.95 (m, 2H), 2.19–2.08 (m, 4H), 1.99–1.89 (m, 8H), 1.19–1.11 (m, 12H), 0.91–0.85 (m, 6H); ^13^C NMR (126 MHz, DMSO-*d6*) δ 170.92, 170.67, 157.08, 143.92, 137.85, 136.80, 119.94, 118.40, 116.49, 60.51, 58.47, 47.56, 46.06, 38.71, 30.11, 24.73, 19.50, 15.08, and 9.48; MS (ESI) m/z: 733.38 [M+ H]^+^.

***Dibutyl((2R,2’R)-((2S,2’S)-(((9H-fluorene-2,7-diyl)bis(azanediyl))bis(carbonyl)) bis(pyrrolidine-2,1-diyl))bis(3-methyl-1-oxobutane-1,2-diyl))dicarbamate (7)***

Yellow oily ; Yield 18.5%; ^1^H NMR (500 MHz, DMSO-*d6*) δ 9.66 (s, 2H), 7.88 (d, J = 5.5 Hz, 2H), 7.71 (dd, J = 8.3, 2.3 Hz, 2H), 7.54 (d, J = 8.0 Hz, 2H), 7.32 (d, J = 8.0 Hz, 2H), 4.48–4.42 (m, 2H), 4.08 (t, J = 8.1 Hz, 2H), 3.98–3.90 (m, 4H), 3.87–3.80 (m, 4H), 3.62 (dd, J = 16.7, 7.5 Hz, 2H), 2.18–2.10 (m, 2H), 2.00–1.91 (m, 8H), 1.54–1.48 (m, 4H), 1.35–1.27 (m, 4H), 0.91–0.83 (m, 18H); ^13^C NMR (126 MHz, DMSO*-d6*) δ 170.95, 170.65, 165.06, 157.20, 143.97, 136.81, 119.91, 118.44, 116.51, 64.31, 60.86, 58.51, 47.56, 38.71, 31.19, 30.09, 24.73, 19.50, 19.02, 18.88, and 14.05; MS (ESI) m/z: 789.45 [M+ H]^+^.

***Dibenzyl((2R,2’R)-((2S,2’S)-(((9H-fluorene-2,7-diyl)bis(azanediyl))bis(carbonyl)) bis(pyrrolidine-2,1-diyl))bis(3-methyl-1-oxobutane-1,2-diyl))dicarbamate (8)***

Yellow oil ; Yield 22%; ^1^H NMR (500 MHz, DMSO-*d6*) δ 9.73 (s, 2H), 7.88 (d, J = 9.5 Hz, 2H), 7.73–7.68 (m, 2H), 7.53 (dd, J = 8.4, 3.6 Hz, 2H), 7.37–7.27 (m, 12H), 5.11–5.00 (m, 4H), 4.51–4.41 (m, 2H), 4.13 (t, J = 8.2 Hz, 2H), 3.83 (d, J = 11.2 Hz, 4H), 3.52–3.46 (m, 2H), 3.09 (qd, J = 7.3, 4.9 Hz, 2H), 1.99–1.92 (m, 4H), 1.17 (t, J = 7.3 Hz, 4H), 0.94–0.82 (m, 12H); ^13^C NMR (101 MHz, DMSO-*d6*) δ 170.75, 170.66, 156.90, 144.03, 143.86, 138.04, 137.43, 137.04, 128.90, 128.08, 119.93, 118.44, 116.50, 70.37, 65.98, 60.85, 58.61, 38.70, 30.13, 24.76, 19.51, and 18.83; MS (ESI) m/z: 857.42 [M+ H]^+^.

***Dimethyl((2S,2’S)-((2S,2’S)-(((9H-fluorene-2,7-diyl)bis(azanediyl))bis(carbonyl))bis(pyrrolidine-2,1-diyl))bis(3-methyl-1-oxopentane-1,2-diyl))dicarbamate (9)***

Yellow oil; Yield 19.4%; ^1^H NMR (400 MHz, CDCl_3_) δ 9.61 (s, 2H), 7.26 (s, 2H), 7.16 (d, J = 7.9 Hz, 4H), 7.12 (d, J = 8.0 Hz, 2H), 5.22 (d, *J* = 8.8 Hz, 2H), 4.68 (dd, *J* = 7.6, 4.4 Hz, 2H), 4.56–4.50 (m, 2H), 3.80–3.68 (m, 6H), 3.59 (s, 4H), 3.17 (s, 2H), 2.53 (dd, *J* = 11.6, 2.2 Hz, 4H), 2.27–1.99 (m, 4H), 1.76–1.43 (m, 4H), 0.94 (dd, *J* = 11.9, 6.6 Hz, 6H), 0.89–0.82 (m, 6H);^13^C NMR (126 MHz, CDCl_3_) δ 172.10, 170.03, 165.52, 156.91, 143.12, 136.76, 118.78, 117.80, 115.65, 61.02, 52.26, 47.70, 41.53, 38.65, 36.72, 29.05, 25.15, 24.60, 23.39, and 21.77; MS (ESI) m/z: 733.38 [M + H]^+^.

***Diethyl((2S,2’S)-((2S,2’S)-(((9H-fluorene-2,7-diyl)bis(azanediyl))bis(carbonyl))bis(pyrrolidine-2,1-diyl))bis(3-methyl-1-oxopentane-1,2-diyl))dicarbamate (10)***

Yellow oil; Yield 29%; ^1^H NMR (500 MHz, DMSO-*d6*) δ 9.67 (s, 2H), 7.36 (d, J = 7.7 Hz, 2H), 7.25 (s, 2H), 7.06 (d, J = 8.5 Hz, 2H), 6.90 (s, 2H), 4.40–4.29 (m, 2H), 4.15–4.03 (m, 2H), 3.88–3.80 (m, 2H), 3.57–3.48 (m, 2H), 2.32–2.13 (m, 4H), 2.00–1.96 (m, 4H), 1.89–1.79 (m, 4H), 1.65–1.56 (m, 8H), 1.43–1.35 (m, 6H), 0.88 (dd, J = 12.7, 4.9 Hz, 12H); ^13^C NMR (126 MHz, DMSO-*d6*) δ 172.06, 171.54, 156.82, 143.92, 137.87, 136.80, 119.94, 118.41, 116.56, 60.45, 60.19, 53.32, 51.33, 31.13, 24.74, 24.64, 23.67, 23.49, 21.86, 15.07, and 9.09; MS (ESI) m/z: 761.42 [M + H]^+^.

***Dibutyl ((2S,2’S)-((2S,2’S)-(((9H-fluorene-2,7-diyl)bis(azanediyl))bis(carbonyl)) bis(pyrrolidine-2,1-diyl))bis(3-methyl-1-oxopentane-1,2-diyl))dicarbamate (11)***

Yellow oil; Yield 28.4%; ^1^H NMR (500 MHz, DMSO-*d6*) δ 10.03 (s, 2H), 7.88 (s, 2H), 7.70 (d, J = 8.3 Hz, 2H), 7.47 (d, J = 1.2 Hz, 2H), 7.27 (d, J = 8.0 Hz, 2H), 4.48 (dd, J = 8.0, 4.6 Hz, 2H), 4.32–4.26 (m, 2H), 3.91 (dd, J = 24.2, 17.6 Hz, 8H), 3.77–3.68 (m, 2H), 3.57 (dt, J = 9.2, 5.7 Hz, 2H), 2.69 (s, 8H), 2.18–2.00 (m, 4H), 1.96–1.89 (m, 4H), 1.58–1.44 (m, 4H), 1.37–1.26 (m, 6H), 0.93–0.86 (m, 12H); ^13^C NMR (126 MHz, DMSO-*d6*) δ 171.33, 170.75, 156.83, 143.99, 138.06, 136.68, 119.95, 118.27, 116.35, 64.03, 60.65, 51.19, 47.19, 38.71, 37.05, 31.21, 29.80, 25.16, 24.61, 23.68, 21.79, 19.04, and 14.08; MS (ESI) m/z: 817.48 [M + H]^+^.

***Dibenzyl((2S,2’S)-((2S,2’S)-(((9H-fluorene-2,7-diyl)bis(azanediyl))bis(carbonyl))bis(pyrrolidine-2,1-diyl))bis(3-methyl-1-oxopentane-1,2-diyl))dicarbamate (12)***

Yellow oil; Yield 23.8%; ^1^H NMR (500 MHz, DMSO-*d6*) δ 10.04 (s, 2H), 7.88 (s, 2H), 7.71 (d, J = 8.3 Hz, 2H), 7.48 (d, J = 5.8 Hz, 2H), 7.39–7.29 (m, 12H), 5.02 (s, 4H), 4.48 (dd, J = 8.0, 4.6 Hz, 2H), 4.32 (t, J = 6.6 Hz, 2H), 3.90–3.74 (m, 2H), 3.70–3.49 (m, 4H), 2.21–2.12 (m, 4H), 1.98–1.84 (m, 4H), 1.55–1.34 (m, 6H), 0.91 (d, J = 6.6 Hz, 12H); ^13^C NMR (126 MHz, DMSO-*d6*) δ 171.19, 170.74,156.58, 143.99, 138.06, 137.55, 136.68, 128.79, 128.26, 128.15, 119.95, 118.28, 116.35, 65.84, 60.68, 51.32, 47.22, 38.80, 31.15, 29.80, 25.16, 24.62, 23.68, and 21.79; MS (ESI) m/z: 885.45 [M+ H]^+^.

***Dimethyl((2R,2’R)-((2S,2’S)-(((9H-fluorene-2,7-diyl)bis(azanediyl))bis(carbonyl))bis(pyrrolidine-2,1-diyl))bis(4-methyl-1-oxopentane-1,2-diyl))dicarbamate (13)***

Yellow oil; Yield 19%**;** ^1^H NMR (400 MHz, CDCl_3_) δ 9.12 (s, 2H), 7.76–7.65 (m, 2H), 7.42 (d, J = 23.6 Hz, 6H), 5.65 (d, J = 5.8 Hz, 2H), 4.67 (d, J = 6.4 Hz, 2H), 4.47 (s, 2H), 3.69–3.57 (m, 6H), 2.00 (dd, J = 11.2, 4.2 Hz, 6H), 1.77–1.64 (m, 4H), 1.60–1.52 (m, 4H), 1.49–1.39 (m, 4H), 1.28 (t, J = 7.3 Hz, 6H), 0.93 (dd, J = 12.1, 6.6 Hz, 6H); ^13^C NMR (126 MHz, CDCl_3_) δ 172.79, 172.22, 169.16, 165.71, 157.36, 143.80, 136.84, 119.22, 118.68, 116.87, 52.39, 51.45, 47.33, 46.75, 36.93, 28.66, 24.64, 23.39, 21.78, and 8.68; MS (ESI) m/z: 733.38 [M + H]^+^.

***Diethyl ((2R,2’R)-((2S,2’S)-(((9H-fluorene-2,7-diyl)bis(azanediyl))bis(carbonyl)) bis(pyrrolidine-2,1-diyl))bis(4-methyl-1-oxopentane-1,2-diyl))dicarbamate (14)***

Yellow oil; Yield 18.7%; ^1^H NMR (500 MHz, DMSO-*d6*) δ 8.85 (s, 2H), 7.39 (dd, J = 26.3, 7.7 Hz, 2H), 7.25 (s, 2H), 7.06 (d, J = 8.4 Hz, 2H), 6.90 (s, 2H), 4.00–3.95 (m, 6H), 3.92–3.78 (m, 4H), 3.48–3.41 (m, 4H), 3.35 (d, J = 2.9 Hz, 6H), 2.04–1.93 (m, 4H), 1.59 (ddd, J = 31.4, 15.6, 9.0 Hz, 4H), 1.49–1.35 (m, 6H), 0.86 (dd, J = 11.9, 6.6 Hz, 12H); ^13^C NMR (126 MHz, DMSO-*d6*) δ 175.06, 171.56, 170.66, 156.83, 156.54, 143.94, 119.95, 118.42, 116.49, 70.25, 60.19, 53.33, 46.26, 31.11, 24.73, 23.79, 23.48, 21.85, 15.05, and 9.07; MS (ESI) m/z: 761.42 [M + H]^+^.

***Dibutyl((2R,2’R)-((2S,2’S)-(((9H-fluorene-2,7-diyl)bis(azanediyl))bis(carbonyl)) bis(pyrrolidine-2,1-diyl))bis(4-methyl-1-oxopentane-1,2-diyl))dicarbamate (15)***

Yellow oil; Yield 21.4%; ^1^H NMR (500 MHz, DMSO-*d6*) δ 10.03 (s, 2H), 7.88 (s, 2H), 7.70 (d, J = 8.3 Hz, 2H), 7.48 (d, J = 8.2 Hz, 2H), 7.26 (d, J = 8.0 Hz, 2H), 4.47 (dd, J = 8.0, 4.6 Hz, 2H), 4.29 (t, J = 7.2 Hz, 2H), 3.93 (dd, J = 13.9, 7.1 Hz, 4H), 3.86 (s, 2H), 3.74–3.53 (m, 6H), 2.25–2.00 (m, 8H), 1.92–1.83 (m, 4H), 1.56–1.45 (m, 8H), 1.36–1.26 (m, 6H), 0.91–0.84 (m, 12H); ^13^C NMR (126 MHz, DMSO-*d6*) δ 171.33, 170.75, 165.06, 156.83, 143.99, 138.06, 119.95, 118.27, 116.34, 64.03, 60.65, 52.25, 51.19, 31.21, 29.79, 25.16, 24.61, 23.68, 23.23, 21.79, 19.04, and 14.08; MS (ESI) m/z: 817.48 [M + H]^+^.

***Dibenzyl((2R,2’R)-((2S,2’S)-(((9H-fluorene-2,7-diyl)bis(azanediyl))bis(carbonyl))bis(pyrrolidine-2,1-diyl))bis(4-methyl-1-oxopentane-1,2-diyl))dicarbamate (16)***

Yellow oil; Yield 25.5%; ^1^H NMR (400 MHz, DMSO-*d6*) δ 10.02 (s, 2H), 7.88 (s, 2H), 7.70 (d, *J* = 8.3 Hz, 2H), 7.48 (d, *J* = 8.0 Hz, 2H), 7.40–7.26 (m, 12H), 5.02 (s, 4H), 4.48 (dd, *J* = 7.8, 4.5 Hz, 2H), 4.39–4.27 (m, 2H), 3.58 (dd, *J* = 14.3, 7.8 Hz, 2H), 2.18–1.99 (m, 4H), 1.96–1.84 (m, 4H), 1.78–1.62 (m, 4H), 1.55–1.41 (m, 4H), 1.40–1.29 (m, 2H), 0.91 (d, *J* = 6.5 Hz, 12H); ^13^C NMR (101 MHz, DMSO-*d6*) δ 171.19, 170.73, 156.57, 143.98, 138.05, 137.55, 136.68, 128.80, 128.25, 128.14, 119.94, 118.28, 116.36, 65.84, 60.67, 55.37, 51.32, 38.71, 29.79, 25.15, 24.61, 23.67, and 21.79; MS (ESI) m/z: 885.45 [M + H]^+^.

***Dimethyl((2S,2’S)-((2S,2’S)-(((9H-fluorene-2,7-diyl)bis(azanediyl))bis (carbonyl)) bis(pyrrolidine-2,1-diyl))bis(4-methyl-1-oxopentane-1,2-diyl))dicarbamate (17)***

Yellow oil; Yield 18.7%; ^1^H NMR (400 MHz, CDCl_3_) δ 9.77 (s, 2H), 7.42–7.34 (m, 4H), 7.21 (d, J = 6.9 Hz, 2H), 7.12 (d, J = 8.1 Hz, 2H), 5.33–5.30 (m, 2H), 4.78 (dd, J = 7.7, 4.9 Hz, 2H), 4.47–4.42 (m, 2H), 3.92 (t, J = 6.7 Hz, 4H), 3.69 (d, J = 14.7 Hz, 6H), 2.31–2.19 (m, 4H), 2.13–2.00 (m, 4H), 1.86–1.62 (m, 4H), 1.26–1.19 (m, 2H), 1.17–1.07 (m, 12H); ^13^C NMR (126 MHz, CDCl_3_) δ 171.77, 170.03, 157.02, 143.05, 136.77, 128.95, 118.78, 117.78, 115.65, 61.11, 56.93, 52.33, 48.13, 38.64, 26.91, 25.19, 15.53, 11.15, and 10.96; MS (ESI) m/z: 733.38 [M + H]^+^.

***Diethyl((2S,2’S)-((2S,2’S)-(((9H-fluorene-2,7 diyl)bis(azanediyl))bis(carbonyl)) bis(pyrrolidine-2,1-diyl))bis(4-methyl-1-oxopentane-1,2-diyl))dicarbamate (18)***

Yellow oil ; Yield 19.5%; ^1^H NMR (500 MHz, DMSO-*d6*) δ 10.05 (s, 2H), 7.89 (s, 2H), 7.71 (d, J = 8.3 Hz, 2H), 7.48 (d, J = 8.2 Hz, 2H), 7.27 (d, J = 8.4 Hz, 2H), 4.48 (dd, J = 8.1, 4.6 Hz, 2H), 4.09 (t, J = 8.8 Hz, 2H), 3.97 (tt, J = 10.8, 5.3 Hz, 4H), 3.88–3.78 (m, 4H), 3.68–3.56 (m, 2H), 2.26–2.07 (m, 4H), 1.96–1.67 (m, 6H), 1.57–1.45 (m, 2H), 1.20–1.09 (m, 8H), 0.96–0.80 (m, 12H);^13^C NMR (101 MHz, DMSO-*d6*) δ 171.03, 170.72, 156.77, 144.01, 138.10, 136.65, 119.95, 118.23, 116.29, 60.72, 60.28, 56.87, 47.73, 36.31, 29.97, 25.10, 24.86, 15.29, 15.10, and 11.02; MS (ESI) m/z: 761.42 [M + H]^+^.

***Dibutyl((2S,2’S)-((2S,2’S)-(((9H-fluorene-2,7-diyl)bis(azanediyl))bis(carbonyl))bis(pyrrolidine-2,1-diyl))bis(4-methyl-1-oxopentane-1,2-diyl))dicarbamate (19)***

Yellow oil ; Yield 24.7%; ^1^H NMR (500 MHz, DMSO-*d6***)** δ 10.05 (s, 2H), 7.89 (s, 2H), 7.70 (d, J = 4.0 Hz, 2H), 7.47 (d, J = 1.4 Hz, 2H), 7.27 (d, J = 8.4 Hz, 2H), 4.48 (dd, J = 8.0, 4.6 Hz, 2H), 4.09 (t, J = 8.8 Hz, 2H), 2.69 (s, 2H), 2.22–2.02 (m, 4H), 1.99–1.87 (m, 4H), 1.73 (d, J = 8.7 Hz, 4H), 1.55–1.45 (m, 8H), 1.36–1.26 (m, 6H), 1.20–1.07 (m, 4H), 0.93–0.78 (m, 18H); ^13^C NMR (101 MHz, DMSO-*d6*) δ 171.03, 170.72, 156.88, 144.01, 138.10, 136.65, 119.95, 118.23, 116.29, 64.09, 60.71, 56.88, 47.70, 36.30, 31.21, 29.96, 25.10, 24.85, 19.03, 15.29, 14.08, and 11.02; MS (ESI) m/z: 817.48 [M + H]^+^.

***Dibenzyl((2S,2’S)-((2S,2’S)-(((9H-fluorene-2,7-diyl)bis(azanediyl))bis(carbonyl))bis(pyrrolidine-2,1-diyl))bis(4-methyl-1-oxopentane-1,2-diyl))dicarbamate (20)***

Yellow oil ; Yield 27.4%; ^1^H NMR (500 MHz, DMSO-*d6***)** δ 10.05 (s, 2H), 7.89 (s, 2H), 7.71 (d, J = 8.3 Hz, 2H), 7.50 (dd, J = 13.0, 8.6 Hz, 4H), 7.40–7.28 (m, 10H), 5.06–4.96 (m, 4H), 4.49 (dd, J = 7.9, 4.6 Hz, 2H), 4.13 (t, J = 8.8 Hz, 2H), 3.86 (s, 4H), 3.70–3.60 (m, 4H), 2.69 (s, 2H), 2.19–1.99 (m, 4H), 1.94–1.86 (m, 4H), 1.59–1.46 (m, 2H), 0.93 (d, J = 6.7 Hz, 12H); ^13^C NMR (101 MHz, DMSO-*d6*) δ 170.90, 170.70, 165.06, 164.78, 156.64, 144.00, 138.10, 136.65, 128.80, 128.24, 128.09, 119.94, 118.23, 116.30, 65.87, 60.73, 57.02, 47.75, 36.34, 29.97, 25.09, 24.84, 15.29, and 11.03; MS (ESI) m/z: 885.45 [M + H]^+^.

***Dimethyl((1S,1’S)-((2S,2’S)-(((9H-fluorene-2,7diyl)bis(azanediyl))bis(carbonyl)) bis(pyrrolidine-2,1-diyl))bis(2-oxo-1-phenylethane-2,1-diyl))dicarbamate (21)***

Yellow oil; Yield 16%; ^1^H NMR (500 MHz, CDCl_3_) δ 9.34 (s, 2H), 8.23 (s, 2H), 7.79 (s, 2H), 7.59 (d, *J* = 15.7 Hz, 2H), 7.42 (d, *J* = 10.2 Hz, 2H), 7.28–7.05 (m, 10H), 5.53 (d, *J* = 7.6 Hz, 2H), 4.50–4.32 (m, 2H), 3.50 (s, 2H), 2.21–2.07 (m, 6H), 1.95–1.80 (m, 4H), 1.59–1.31 (m, 4H), 1.23–1.03 (m, 4H); ^13^C NMR (126 MHz, CDCl_3_) δ 172.92, 170.38, 169.49, 168.84, 156.38, 143.77, 137.42, 129.34, 128.88, 127.87, 119.37, 118.34, 116.34, 58.50, 56.90, 52.22, 47.76, 29.43, 24.78, and 22.25; MS (ESI) m/z: 773.32 [M + H]^+^.

***Diethyl((1S,1’S)-((2S,2’S)-(((9H-fluorene-2,7-diyl)bis(azanediyl))bis(carbonyl)) bis(pyrrolidine-2,1-diyl))bis(2-oxo-1-phenylethane-2,1-diyl))dicarbamate (22)***

Yellow oil; Yield 24%; ^1^H NMR (500 MHz, CDCl_3_) δ 9.46 (s, 2H), 7.78 (s, 2H), 7.50 (d, J = 14.6 Hz, 6H), 7.41 (d, J = 6.8 Hz, 10 H), 5.91 (s, 2H), 5.66–5.51 (m, 2H), 5.00–4.73 (m, 4H), 3.72 (s, 2H), 3.31–3.12 (m, 4H), 2.14–2.00 (m, 8H), 1.43–1.29 (m, 6H); ^13^C NMR (126 MHz, CDCl_3_) δ 168.24, 167.20, 163.40, 154.04, 141.67, 135.29, 134.64, 134.39, 127.37, 125.99, 117.32, 116.33, 114.29, 59.32, 54.86, 45.32, 36.85, 26.11, 23.09, 12.63, and 6.79; MS (ESI) m/z: 801.35 [M + H] ^+^.

***Dibutyl((1S,1’S)-((2S,2’S)-(((9H-fluorene-2,7-diyl)bis(azanediyl))bis(carbonyl))bis(pyrrolidine-2,1-diyl))bis(2-oxo-1-phenylethane-2,1-diyl))dicarbamate (23)***

Yellow oil; Yield 23.5%; ^1^H NMR (500 MHz, DMSO-*d6***)** δ 10.07 (s, 2H), 7.87 (s, 2H), 7.71 (d, J = 2.6 Hz, 2H), 7.55 (d, J = 8.3 Hz, 2H), 7.44 (d, J = 7.2 Hz, 2H), 7.40–7.29 (m, 10H), 5.47 (d, J = 8.3 Hz, 2H), 4.52 (dd, J = 8.1, 4.6 Hz, 2H), 3.95 (tt, J = 6.5, 3.2 Hz, 4H), 3.88 (s, 2H), 3.72–3.61 (m, 4H), 2.23–2.09 (m, 4H), 1.91–1.85 (m, 4H), 1.55–1.47 (m, 4H), 1.35–1.26 (m, 4H), 0.89–0.85 (m, 6H); ^13^C NMR (126 MHz, DMSO-*d6*) δ 170.55, 168.62, 156.46, 144.02, 138.03, 137.43, 136.70, 128.85, 128.54, 128.23, 120.01, 118.30, 116.37, 64.22, 61.03, 57.02, 55.38, 38.72, 31.19, 29.82, 25.12, 19.02, and 14.08; MS (ESI) m/z: 857.42 [M + H]^+^.

***Dibenzyl((1S,1’S)-((2S,2’S)-(((9H-fluorene-2,7-diyl)bis(azanediyl))bis(carbonyl)) bis(pyrrolidine-2,1-diyl))bis(2-oxo-1-phenylethane-2,1-diyl))dicarbamate (24)***

Yellow oil; Yield 20.3%; ^1^H NMR (500 MHz, CDCl_3_) δ 9.68–9.46 (m, 2H), 7.52 (d, *J* = 6.9 Hz, 2H), 7.47 (dd, *J* = 12.8, 6.7 Hz, 2H), 7.40 (dd, *J* = 14.8, 7.5 Hz, 4H), 7.31 (d, *J* = 6.7 Hz, 10 H), 6.21–5.90 (m, 2H), 5.65–5.51 (m, 2H), 5.15–5.03 (m, 4H), 4.92–4.63 (m, 2H), 3.81–3.60 (m, 2H), 3.49–3.29 (m, 4H), 3.03–2.87 (m, 8H), 2.23–1.93 (m, 12H); ^13^C NMR (126 MHz, CDCl_3_) δ 169.68, 169.24, 165.62, 155.70, 143.42, 137.06, 136.58, 136.18, 129.27, 128.52, 128.19, 128.04, 119.09, 118.08, 116.00, 66.99, 61.22, 56.89, 47.46, 29.70, 28.49, and 25.01; MS (ESI) m/z: 925.38 [M + H]^+^.

***Dimethyl((1R,1’R)-((2S,2’S)-(((9H-fluorene-2,7-diyl)bis(azanediyl))bis(carbonyl)) bis(pyrrolidine-2,1-diyl))bis(2-oxo-1-phenylethane-2,1-diyl))dicarbamate (25)***

Yellow oil; Yield 17.75%; ^1^H NMR (500 MHz, DMSO-*d6*) δ 8.13–8.00 (m, 6H), 7.86–7.78 (m, 2H), 7.67–7.57 (m, 2H), 7.46–7.19 (m, 10H), 3.75–3.43 (m, 4H), 2.63–2.50 (m, 2H), 2.36 (dt, *J* = 3.6, 1.8 Hz, 6H), 1.67–1.51 (m, 4H), 1.21–0.99 (m, 4H), 0.96–0.61 (m, 4H); ^13^C NMR (126 MHz, DMSO-*d6*) δ 177.29, 171.77, 168.82, 156.86, 142.77, 136.78, 129.95, 129.02, 128.81, 128.24, 125.11, 120.02, 110.60, 66.76, 62.17, 58.47, 52.72, 52.15, 49.83, and 21.68; MS (ESI) m/z: 773.32 [M + H]^+^.

***Diethyl((1R,1’R)-((2S,2’S)-(((9H-fluorene-2,7-diyl)bis(azanediyl))bis(carbonyl))bis(pyrrolidine-2,1-diyl))bis(2-oxo-1-phenylethane-2,1-diyl))dicarbamate (26)***

Yellow oil; Yield 18%; ^1^H NMR (500 MHz, CDCl_3_*-d*) δ 9.43 (d, *J* = 11.9 Hz, 2H), 7.74 (d, *J* = 16.3 Hz, 2H), 7.65 (s, 2H), 7.47 (d, *J* = 8.4 Hz, 4H), 7.42–7.35 (m, 10H), 6.05 (dd, *J* = 13.9, 6.7 Hz, 2H), 5.58–5.52 (m, 2H), 4.89 (s, 2H), 3.69 (dd, *J* = 16.8, 7.4 Hz, 4H), 3.34 (dd, *J* = 17.3, 9.2 Hz, 4H), 2.38 (dd, *J* = 19.5, 11.9 Hz, 4H), 2.13–2.01 (m, 4H), 1.25–1.21 (m, 6H); ^13^C NMR (126 MHz, CDCl_3_*-d*) δ 170.43, 170.19, 169.03, 155.97, 143.71, 136.74, 136.30, 129.22, 128.83, 127.96, 119.34, 116.26, 109.60, 61.60, 61.33, 56.78, 47.47, 36.98, 29.70, 27.47, and 14.66; MS (ESI) m/z: 801.35 [M + H]^+^. 

***Dibutyl((1R,1’R)-((2S,2’S)-(((9H-fluorene-2,7-diyl)bis(azanediyl))bis(carbonyl))bis(pyrrolidine-2,1-diyl))bis(2-oxo-1-phenylethane-2,1-diyl))dicarbamate (27)***

Yellow oil; Yield 20.6%; ^1^H NMR (400 MHz, CDCl_3_-*d*) δ 9.22 (s, 2H), 7.90 (s, 2H), 7.82 (s, 2H), 7.62 (d, J = 7.9 Hz, 2H), 7.56–7.36 (m, 10H), 7.24–7.13 (m, 2H), 5.90 (s, 2H), 5.50 (s, 2H), 4.93–4.76 (m, 2H), 3.67–3.53 (m, 4H), 3.35–3.17 (m, 4H), 2.44–2.32 (m, 4H), 2.12–1.88 (m, 4H), 1.41–1.31 (m, 8H), 0.98–0.86 (m, 6H),^13^C NMR (126 MHz, CDCl_3_-*d*) δ 170.31, 169.76, 164.89, 162.51, 147.97, 143.78, 130.27, 129.46, 127.88, 125.85, 119.35, 110.75, 94.04, 66.00, 45.68, 32.12, 31.09, 29.77, 25.58, 22.99, 19.08, 14.66, and 13.89; MS (ESI) m/z: 857.42 [M + H]^+^.

***Dibenzyl((1R,1’R)-((2S,2’S)-(((9H-fluorene-2,7-diyl)bis(azanediyl))bis(carbonyl)) Bis(pyrrolidine-2,1-diyl))bis(2-oxo-1-phenylethane-2,1-diyl))dicarbamate (28)***

Yellow oil; Yield 22%; ^1^H NMR (500 MHz, DMSO-*d6*) δ 10.11 (s, 2H), 7.89 (d, J = 16.4 Hz, 2H), 7.79 (s, 2H), 7.72 (d, J = 3.4 Hz, 2H), 7.55 (d, J = 7.4 Hz, 2H), 7.51–7.41 (m, 20H), 5.51–5.46 (m, 2H), 5.04 (s, 4H), 3.88 (s, 2H), 3.13 (dd, J = 18.0, 11.0 Hz, 2H), 2.25–2.01 (m, 4H), 1.93–1.77 (m, 8H); ^13^C NMR (126 MHz, DMSO-*d6*) δ 170.56, 169.18, 168.57, 156.30, 155.98, 149.61, 144.06, 138.43, 137.49, 137.33, 136.70, 129.06, 128.55, 127.88, 120.06, 118.32, 116.36, 66.11, 61.05, 57.15, 47.36, 37.07, 29.82, 25.13; MS (ESI) m/z: 925.38 [M + H]^+^.

***Dimethyl ((((9H-fluorene-2,7-diyl)bis(azanediyl))bis(carbonyl))bis(piperidine-3,1-diyl))bis(3-methyl-1-oxobutane-1,2-diyl))dicarbamate (29)***

Yellow oil; Yield 18%;^1^H NMR (400 MHz, CDCl_3_-*d*) δ 7.85 (d, J = 11.0 Hz, 2H), 7.65–7.61 (m, 2H), 7.45 (s, 2H), 7.33 (d, J = 13.4 Hz, 2H), 7.26 (s, 2H), 5.12 (s, 2H), 4.32 (s, 2H), 3.84 (s, 6H), 3.69–3.62 (m, 4H), 3.58–3.44 (m, 4H), 2.58 (t, J = 12 Hz, 2H), 2.37–2.22 (m, 2H), 2.09–1.96 (m, 4H), 1.41–1.24 (m, 4H), 1.23 (s, 12H); ^13^C NMR (126 MHz, CDCl_3_*-d*) δ 171.03, 170.65, 167.73, 132.47, 128.87, 119.69, 118.91, 116.94, 68.17, 49.14, 39.09, 38.74, 37.01, 30.37, 28.93, 23.76, 22.99, 14.05, and 10.96; MS (ESI) m/z: 733.38 [M + H]^+^.

***Dimethyl ((((9H-fluorene-2,7-diyl)bis(azanediyl))bis(carbonyl))bis(piperidine-3,1-diyl))bis(4-methyl-1-oxopentane-1,2-diyl))dicarbamate (30)***

Yellow oil; Yield 17.5%; ^1^H NMR (500 MHz, CDCl_3_-*d*) δ 7.75–7.71 (m, 4H), 7.57–7.53 (m, 6H), 4.27–4.19 (m, 4H), 3.79–3.74 (m, 4H), 3.73–3.67 (m, 6H), 1.73–1.71 (m, 2H), 1.70–1.67 (m, 4H), 1.47–1.43 (m, 4H), 1.44–1.41 (m, 2H), 1.40–1.37 (m, 4H), 1.01 (dd, *J* = 12.1, 5.2 Hz, 4H), 0.92 (s, 12H); ^13^C NMR (126 MHz, CDCl_3_*-d*) δ 171.03, 170.65, 167.73, 132.47, 130.89, 128.87, 119.69, 118.91, 116.94, 68.17, 49.14, 39.09, 38.74, 37.01, 30.37, 28.93, 23.76, 22.99, 14.05, and 10.96; MS (ESI) m/z: 761.42 [M + H]^+^.

***Dimethyl ((((9H-fluorene-2,7-diyl)bis(azanediyl))bis(carbonyl))bis(piperidine-3,1-diyl))bis(3-methyl-1-oxopentane-1,2-diyl))dicarbamate (31)***

Yellow oil; Yield 16%; ^1^H NMR (500 MHz, DMSO-*d6*) δ 10.05 (s, 2H), 7.88 (s, 2H), 7.70 (d, J = 7.4 Hz, 2H), 7.51 (d, J = 8.3 Hz, 2H), 7.30 (d, J = 8.7 Hz, 2H), 4.12 (s, 2H), 3.86 (s, 2H), 3.17–3.00 (m, 8H), 2.85–2.74 (m, 4H), 2.62 (dd, J = 18.1, 16.4 Hz, 6H), 2.07–1.94 (m, 8H), 1.78–1.65 (m, 4H), 1.46 (d, J = 7.4 Hz, 6H), 0.87–0.79 (m, 6H); ^13^C NMR (126 MHz, DMSO- *d6*) δ 173.50, 168.73, 161.01, 152.03, 130.20, 120.21, 115.08, 110.60, 81.39, 66.64, 62.46, 54.79, 47.70, 44.82, 44.17, 37.73, 33.62, 29.42, 24.58, 22.69, and 12.71; MS (ESI) m/z: 761.42 [M + H]^+^.

***Dimethyl((((9H-fluorene-2,7-diyl)bis(azanediyl))bis(carbonyl))bis(piperidine-3,1-diyl))bis(2-oxo-1-phenylethane-2,1-diyl))dicarbamate (32)***

Yellow oil; Yield 15%; ^1^H NMR (400 MHz, CDCl_3_-*d*) δ 7.45–7.36 (m, 10H), 7.15–6.99 (m, 5H), 6.80–6.67 (m, 5H), 4.05–3.86 (m, 4H), 3.81–3.57 (m, 8H), 1.39–1.27 (m, 2H), 1.23 (s, 6H), 0.89–0.77 (m, 8H); ^13^C NMR (126 MHz, CDCl_3_*-d*) δ 171.47, 171.25, 165.77, 161.64, 153.89, 145.04, 138.20, 129.97, 129.27, 127.73, 116.82, 115.53, 115.27, 71.13, 63.75, 46.76, 40.13, 31.93, 29.70, 22.70, and 14.12; MS (ESI) m/z: 801.35 [M + H]^+^.

### 3.2. Biological Assays

#### 3.2.1. Cell Culture

The stable replicon cell line Huh5-2 (genotype 1b; Con1) (kindly provided by R. Bartenschlager, Heidelberg University, Germany) has been described previously [22]. The stable cell lines Huh7.5-3a and Huh7.5-4a have been constructed [27] with the subgenomic reporter replicons of HCV 3a (S52) S52-SG(Feo)(AII) and HCV 4a (ED43) ED43-SG(Feo)(VYG) (kindly provided by C.M. Rice, The Rockefeller University, NY) [28]. Cells were cultured in high glucose (25 mM) Dulbecco’s modified minimal essential medium (Invitrogen), supplemented with 2 mM L-glutamine, 0.1 mM non-essential amino acids, 100 U/mL penicillin, 100 µg/mL streptomycin, and 10% (*v/v*) fetal calf serum (referred to as complete DMEM). Complete DMEM was supplemented with G418 at 500 μg/mL for Huh5-2, 750 μg/mL for Huh7.5-3a, and 350 μg/mL for Huh7.5-4a. 

#### 3.2.2. Cell-Based Antiviral and Cytotoxicity Assays 

Cells were treated with serial dilutions of the test compounds or the solvent DMSO for 72 h. Antiviral activity and cytotoxicity were determined by measuring virus-derived luciferase activity or intracellular ATP levels, respectively. The median effective concentration (EC_50_) of the compounds, reducing luciferase signal by 50%, and their median cytotoxic concentration (CC_50_), causing 50% cell death, were determined by nonlinear regression analysis using the Prism 6.0 software (GraphPad Software Inc.). Daclatasvir was kindly provided by Dr. Marc Windisch (Institut Pasteur Korea).

#### 3.2.3. Luciferase and Bradford Assays 

Firefly luciferase activity was measured using Luciferase Assay System (Promega), according to the manufacturer’s instructions. Measurements were performed with a GloMax 20/20 single tube luminometer (Promega) for 10 s. Values were normalized to total protein amounts as quantified by Bradford assay (Pierce).

#### 3.2.4. Measurement of Intracellular ATP Levels 

ATP was measured using the ViaLight HS BioAssay kit (Lonza) according to the manufacturer’s protocol in a GloMax 20/20 single-tube luminometer (Promega) for 1 s. Values were normalized to total protein amounts.

#### 3.2.5. Gel Electrophoresis and Western Blot Analysis

Denaturing SDS-polyacrylamide gel electrophoresis and Western blotting were performed as described previously [29]. Dilutions of 1:2000 for HCV NS5A (9E10) monoclonal antibody (kindly provided by C. Rice), 1:6000 for β-actin monoclonal antibody (Merck-Millipore), and 1:2000 for the secondary anti-mouse horseradish peroxidase-conjugated antibody (Cell Signaling) were used. Image quantification analysis was performed with Quantity I Bio-Rad software.

#### 3.2.6. Total RNA Extraction and Quantification of Viral Replicons

Total RNA was extracted from Huh5-2 cells with Nucleozol reagent (Macherey-Nagel), according to the manufacturer’s instructions, and used to perform reverse-transcription (RT) and quantitative real-time polymerase chain reaction (qPCR) for replicon RNA. RT was performed using Moloney Murine Leukemia Virus (MMLV) reverse transcriptase (Promega) and reverse primers specific for Con1 IRES (5’-GGATTCGTGCTCATGGTGCA-3’) and the housekeeping gene YWHAZ (5’- GGATGTGTTGGTTGCATTTCCT-3’). For qPCR, primers specific for the Con1 IRES (forward: 5’-GGCCTTGTGGTACTGCCTGATA-3’and reverse: 5’-GGATTCGTGCTCATGGTGCA-3’) and KAPA SYBR FAST qPCR Master Mix (Kapa Biosystems) were used. YWHAZ mRNA was used as a normalization control (forward: 5’-GCTGGTGATGACAAGAAAGG-3’ and reverse: 5’- GGATGTGTTGGTTGCATTTCCT-3’). 

#### 3.2.7. Statistical Analysis

In all diagrams, bars represent the mean values of three independent experiments in triplicate. Error bars represent standard deviation. Only results subjected to statistical analysis using Student’s *t-*test with *p* ≤ 0.05 were considered as statistically significant and presented. Statistical calculations were performed with Excel Microsoft Office^®^. 

## 4. Conclusions

Novel 2,7-diaminofluorene-*S*-prolinamide or 2,7-diaminofluorene-(*S,R*)-piperidine-3-caboxamide bearing several capping groups were prepared and evaluated for their anti-HCV activity. We concluded that the 2,7-diaminofluorene-*S*-prolinamide core analogs exhibited better activity over the 2,7-diaminofluorene-(*S,R*)-piperidine-3-carboxamide core counterparts, a fact that can be attributed to the extra methylene carbon in the piperidine carboxamide analogs, which forces a conformation and changes the relative orientation of some essential groups in the binding site. Additionally, the derivatives with *R*-phenylglycine as a terminal capping group were more potent than the frequently tested *S*-valine analogs. This can help to design of novel NS5A inhibitors with great diversity. Our findings encourage the development of new peptidomimetic analogs. They support the successful implementation of the peptidomimetic approach for the development of novel NS5A inhibitors with improved physicochemical and pharmacokinetic properties. This work will be extended to investigate the conformational and stereoelectronic aspects of the core scaffold and obtain more SAR information to help refine the critical requirements for optimal activity.

## Figures and Tables

**Table 1:** Effective concentration (EC_50_), cytotoxic concentration (CC_50_), and selectivity index (SI_50_) of compounds (**1**–**28**) tested on Hepatitis C virus (HCV) genotype (GT) 1b replicons.

Code	R	Stereo-Chemistry *	R΄	HCV Replicon 1b		
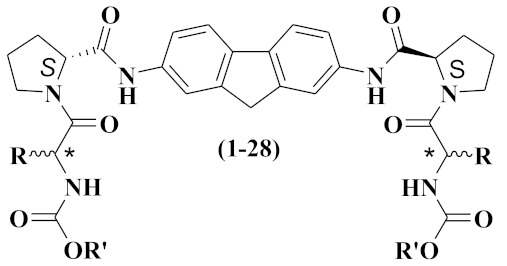						
**1**	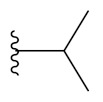	*S*	-CH_3_	**EC**_**50**_ **(nM)**	**CC**_**50**_ **(nM)**	**SI**_**50**_^**a**^
**2**	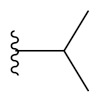	*S*	-CH_2_CH_3_	>1000	>100,000	ND ^b^
**3**	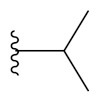	*S*	-C_4_H_9_	>1000	>100,000	ND
**4**	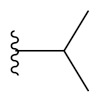	*S*	-CH_2_C_6_H_5_	966.90	>100,000	>103
**5**	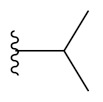	*R*	-CH_3_	801.40	>100,000	>125
**6**	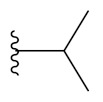	*R*	-CH_2_CH_3_	102	79,550	780
**7**	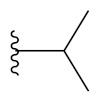	*R*	-C_4_H_9_	981.40	>100,000	>102
**8**	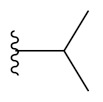	*R*	-CH_2_C_6_H_5_	45.42	>100,000	>2202
**9**	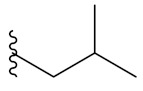	*S*	-CH_3_	27.50	>100,000	>3636
**10**	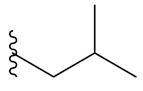	*S*	-CH_2_CH_3_	0.137	85,510	622,344
**11**	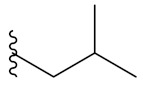	*S*	-C_4_H_9_	976.60	65,950	68
**12**	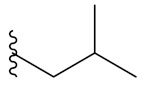	*S*	-CH_2_C_6_H_5_	>1000	46,450	ND
**13**	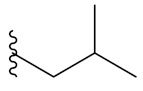	*R*	-CH_3_	0.081	>100,000	>1,238,850
**14**	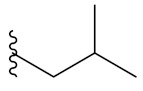	*R*	-CH_2_CH_3_	2.55	>100,000	>39,185
**15**	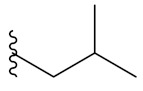	*R*	-C_4_H_9_	>1000	34,220	ND
**16**	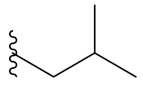	*R*	-CH_2_C_6_H_5_	>1000	>100,000	ND
**17**	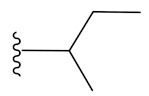	*S*	-CH_3_	10.66	>100,000	>9381
**18**	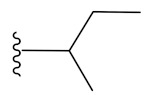	*S*	-CH_2_CH_3_	150.60	>100,000	>664
**19**	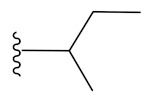	*S*	-C_4_H_9_	>1000	>100,000	ND
**20**	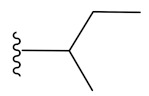	*S*	-CH_2_C_6_H_5_	>1000	98,730	ND
**21**	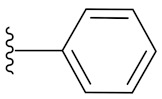	*S*	-CH_3_	8.50	87,510	10,298
**22**	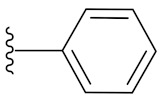	*S*	-CH_2_CH_3_	7.49	>100,000	>13,349
**23**	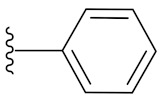	*S*	-C_4_H_9_	2.25	>100,000	>44,444
**24**	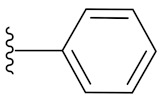	*S*	-CH_2_C_6_H_5_	1.12	>100,000	>89,606
**25**	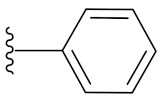	*R*	-CH_3_	0.142	95,390	669,874
**26**	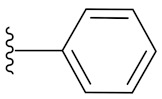	*R*	-CH_2_CH_3_	0.036	>100,000	>2,779,322
**27**	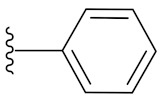	*R*	-C_4_H_9_	4.03	>100,000	>24,795
**28**	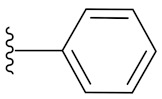	*R*	-CH_2_C_6_H_5_	94.55	>100,000	>1,058
**Daclatasvir**	---	---	---	0.027	17,700	655,556
**IV**	---	---	---	>1000	44990	ND

^a^ SI_50_ = CC_50_/EC_50_; ^b^ ND: Not Determined; * absolute configuration of terminal AA.

**Table 2:** EC_50_, CC_50_, and SI_50_ of compounds (**29–32**) tested on HCV (GT)1b replicon.

Code	R	HCV Replicon 1b		
		EC_50_ (nM)	CC_50_ (nM)	SI_50_^a^
* 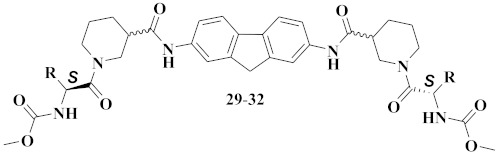 *				
**29**	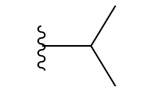	342.20	>100,000	>292
**30**	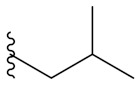	289.10	>100,000	>346
**31**	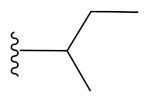	295.50	98,280	333
32	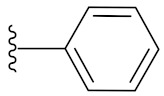	304.50	>100,000	>328

^a^ SI_50_ = CC_50_/EC_50._

## Data Availability

Data is contained within the article or supplementary material.

## Supplementary Materials

The following are available online at https://www.mdpi.com/1424-8247/14/4/292/s1. Figure S1: ^1^H-NMR and ^13^C-NMR spectra of selected compounds.
