# Supplementary material for: Design and Synthesis of Novel Symmetric Fluorene-2,7-Diamine Derivatives as Potent Hepatitis C Virus Inhibitors
Source: Pharmaceuticals (Basel). 2021 Mar 25;14(4):292. doi: 10.3390/ph14040292 (PMC8064491; doi:10.3390/ph14040292)
Supplement: Supplementary file 1 [file pharmaceuticals-14-00292-s001.pdf]

Supplementary Information

Design and synthesis of novel symmetric diaminofluorene prolinamide analogues as potent anti-hepatitis C virus

AUTHOR NAMES

Mai H. A. Mousa, Nermin S. Ahmed*±, Efseveia Frakolaki§, Niki Vassilaki§, Grigoris Zoidis†, Jan J. Weigand‡ and Ashraf H. Abadi**

AUTHOR ADDRESS

*Department of Pharmaceutical Chemistry, Faculty of Pharmacy and Biotechnology, German University in Cairo, Cairo 11835, Egypt.

§Molecular Virology Laboratory, Hellenic Pasteur Institute, 127 Vas. Sofias Avenue, 11521, Athens, Greece.

†Department of Pharmacy, Division of Pharmaceutical Chemistry, School of Health Sciences, National and Kapodistrian University of Athens, 15771, Athens, Greece.

‡ Faculty of Chemistry and Food Chemistry, Technische Universität Dresden, 01062 Dresden, Germany

±To whom correspondences should be addressed: email: nermin.salah@guc.edu.eg; Tel: +202-27590700 Ext: 3429; Fax: +202-27581041

Contents

^{1}H-NMR and ^{13}C-NMR spectra of selected compounds ....page S3

^{1}H-NMR and ^{13}C-NMR spectra of selected compounds

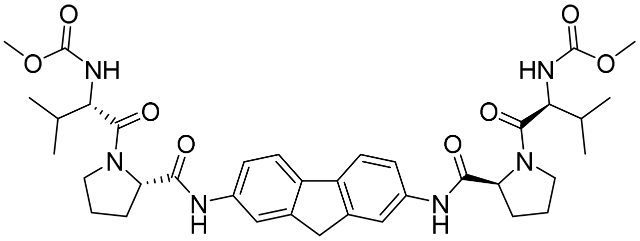

1

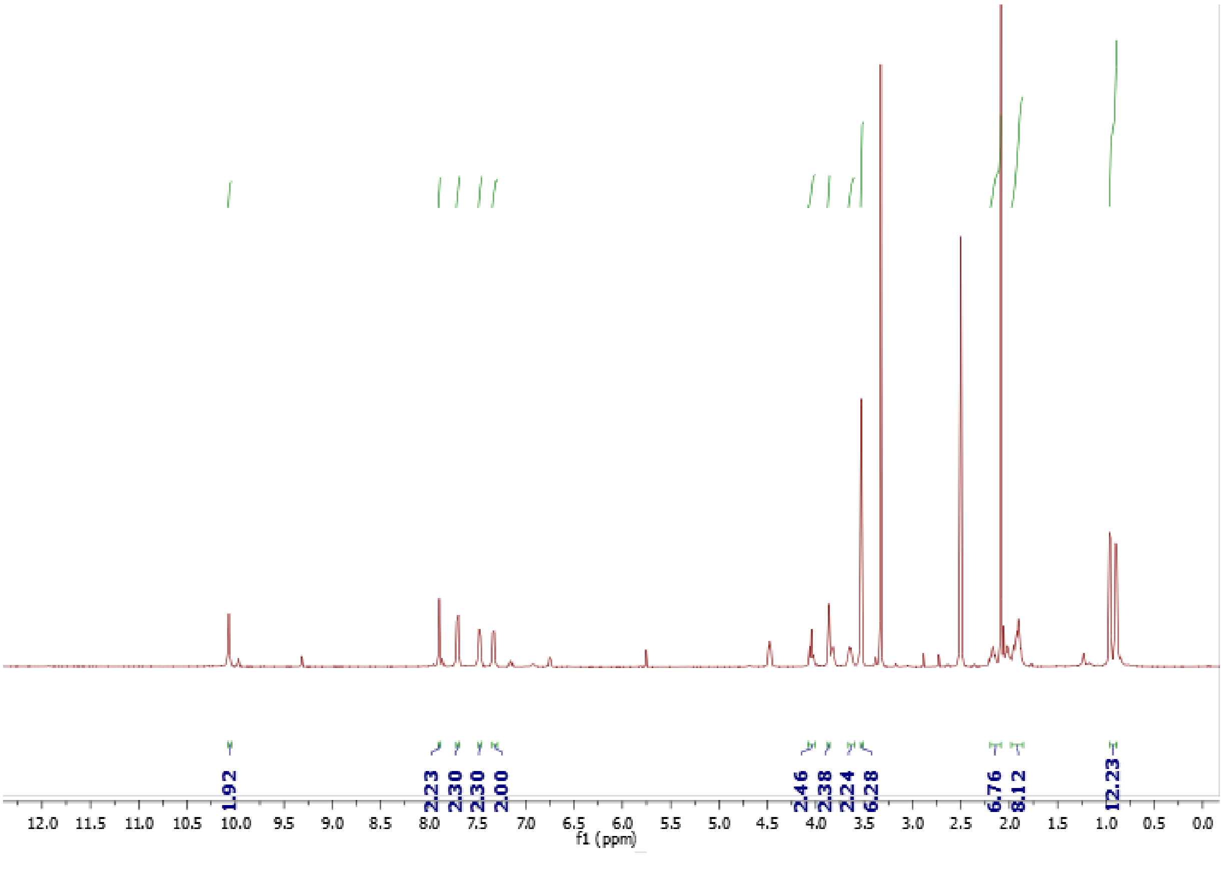

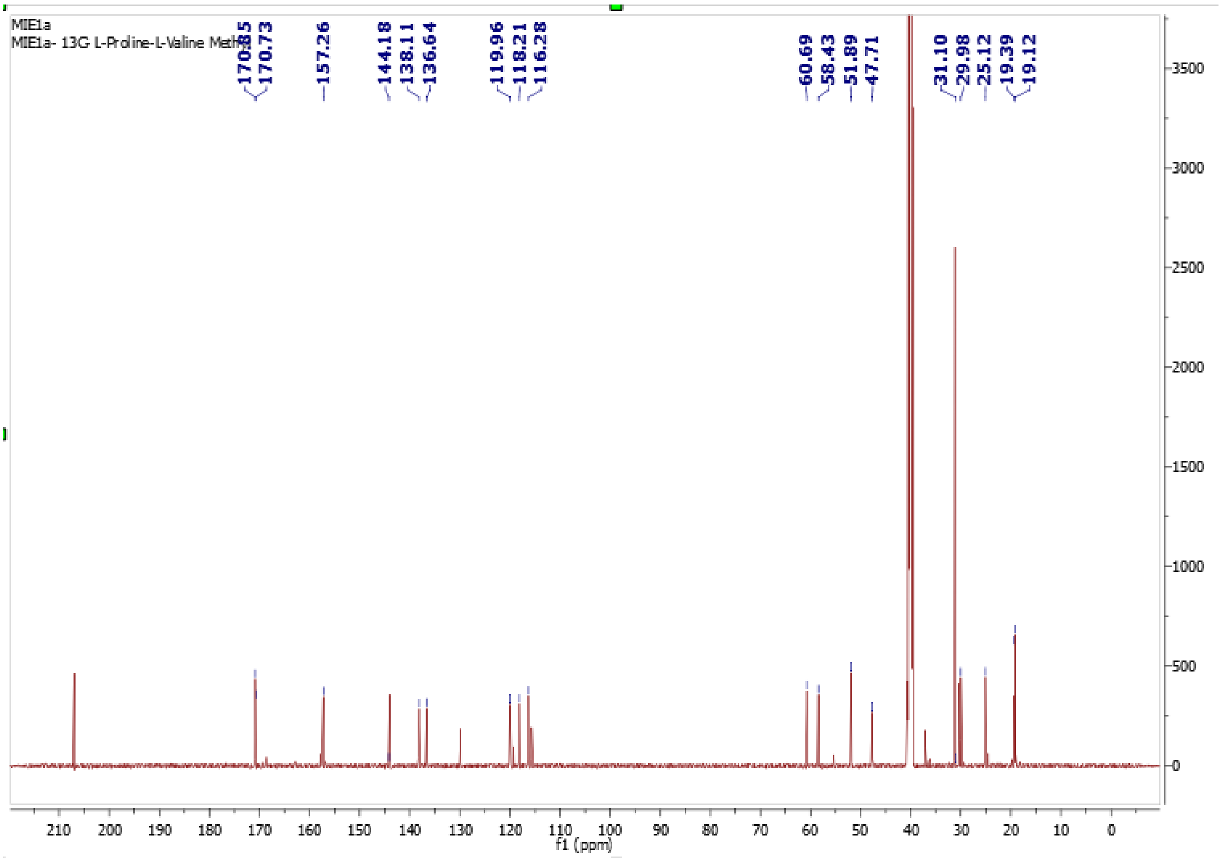
MIE1a
MIE1a- 13C L-Proline-L-Valine Meth
170.85
170.73
157.26
144.18
138.11
136.64
119.96
118.21
116.28
60.69
58.43
51.89
47.71
31.10
29.98
25.12
19.39
19.12
f1 (ppm)

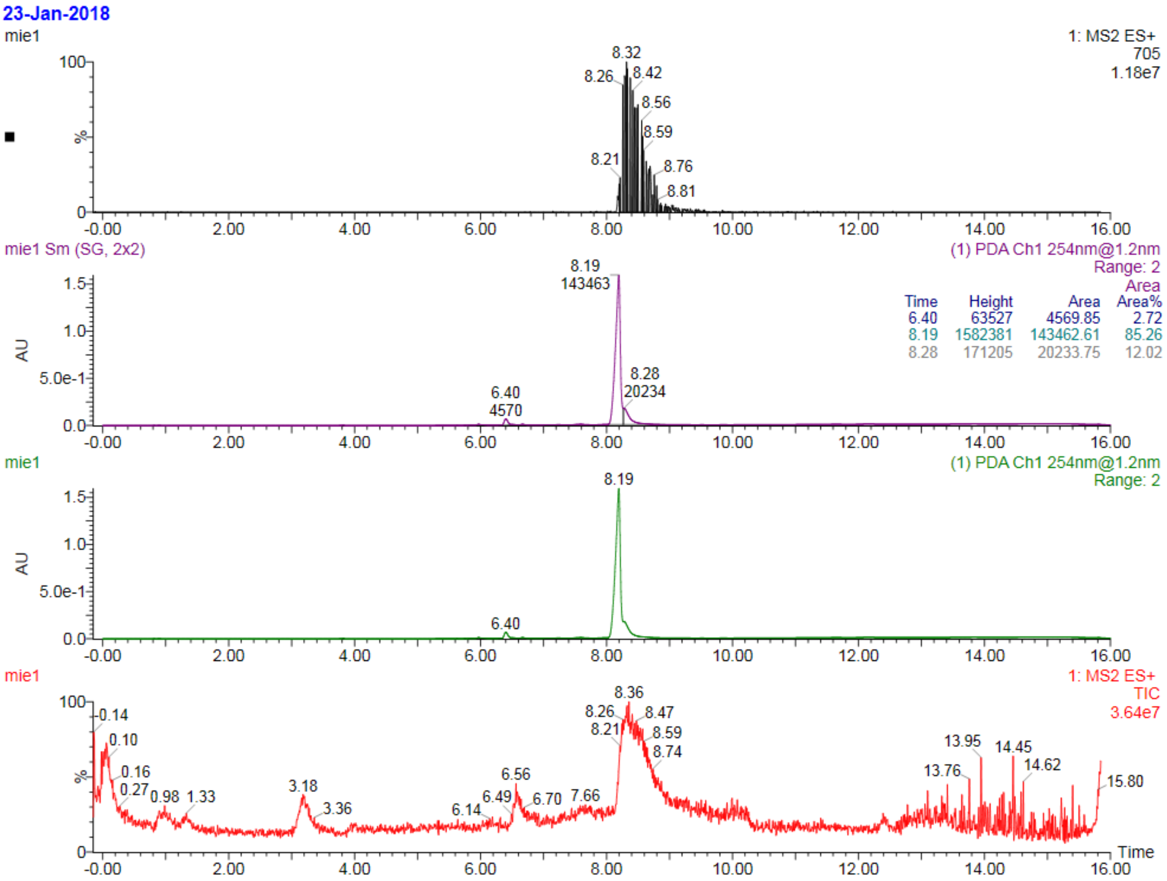

23-Jan-2018
mie1
1: MS2 ES+
705
1.18e7
8.32
8.26
8.42
8.56
8.59
8.21
8.76
8.81
mie1 Sm (SG, 2x2)
(1) PDA Ch1 254nm@1.2nm
Range: 2
8.19
143463
8.28
20234
6.40
4570
Time Height Area Area%
6.40 63527 4569.85 2.72
8.19 1582381 143462.61 85.26
8.28 171205 20233.75 12.02
mie1
(1) PDA Ch1 254nm@1.2nm
Range: 2
8.19
6.40
mie1
1: MS2 ES+
TIC
3.64e7
8.36
8.26
8.47
8.21
8.59
8.74
0.14
0.10
0.16
0.27
0.98
1.33
3.18
3.36
6.56
6.49
6.14
6.70
7.66
13.95
14.45
13.76
14.62
15.80
Time

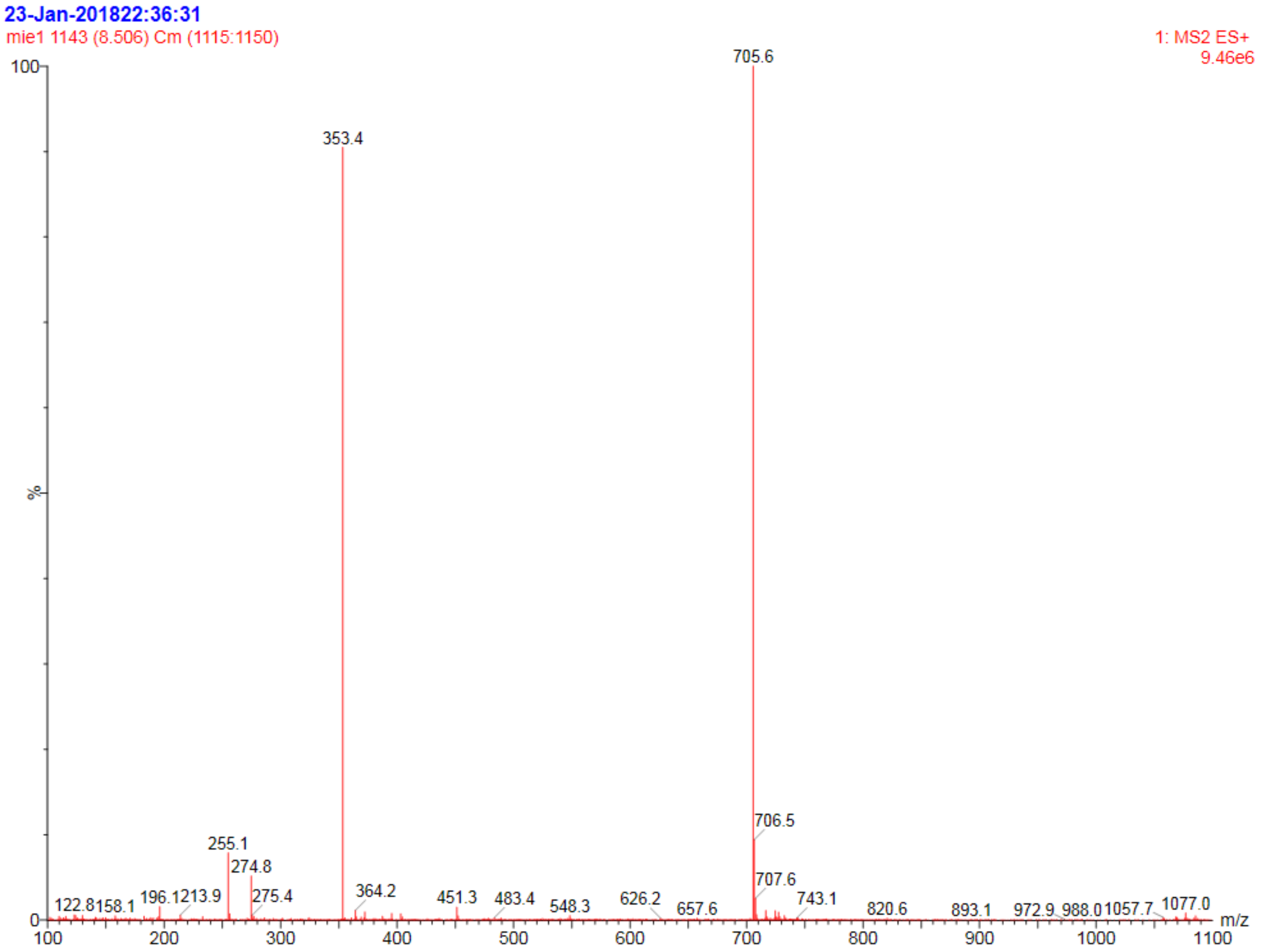

23-Jan-201822:36:31
mie1 1143 (8.506) Cm (1115:1150)
1: MS2 ES+
9.46e6
100
%
0
705.6
353.4
706.5
255.1
274.8
707.6
122.8
158.1
196.1
213.9
275.4
364.2
451.3
483.4
548.3
626.2
657.6
743.1
820.6
893.1
972.9
988.0
1057.7
1077.0
m/z
100
200
300
400
500
600
700
800
900
1000
1100

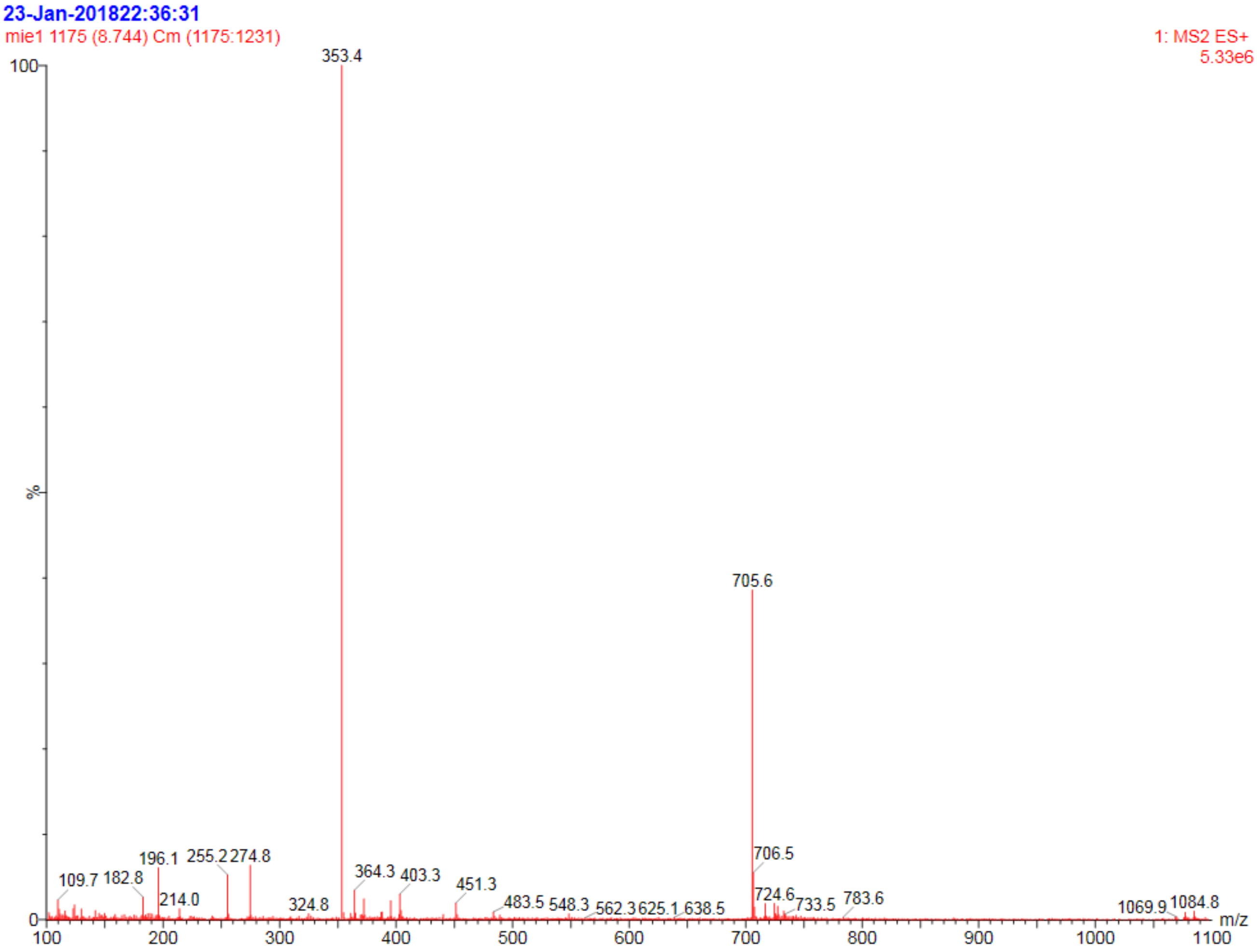

23-Jan-201822:36:31
mie1 1175 (8.744) Cm (1175:1231)
1: MS2 ES+
5.33e6
353.4
705.6
706.5
196.1
255.2
274.8
109.7
182.8
214.0
324.8
364.3
403.3
451.3
483.5
548.3
562.3
625.1
638.5
724.6
733.5
783.6
1069.9
1084.8
%
m/z

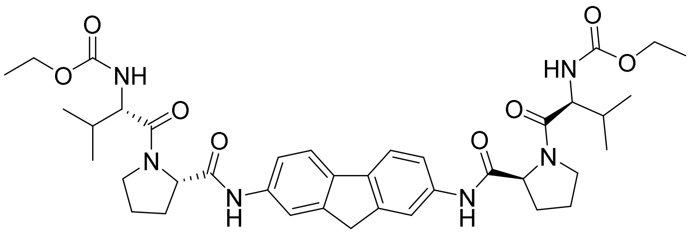

2

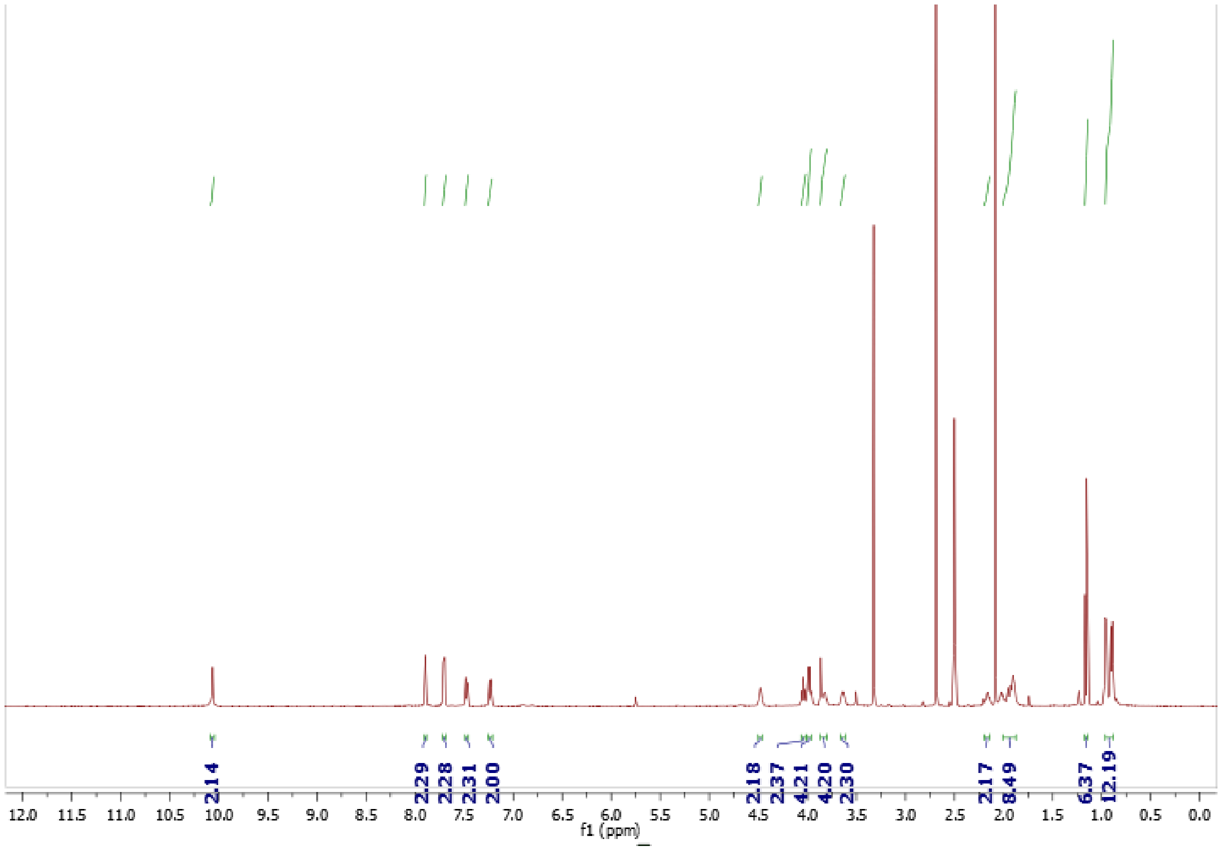

2.14
2.29
2.28
2.31
2.00
2.18
2.37
4.21
4.20
2.30
2.17
8.49
6.37
12.19
12.0
11.5
11.0
10.5
10.0
9.5
9.0
8.5
8.0
7.5
7.0
6.5
6.0
5.5
5.0
4.5
4.0
3.5
3.0
2.5
2.0
1.5
1.0
0.5
0.0
f1 (ppm)

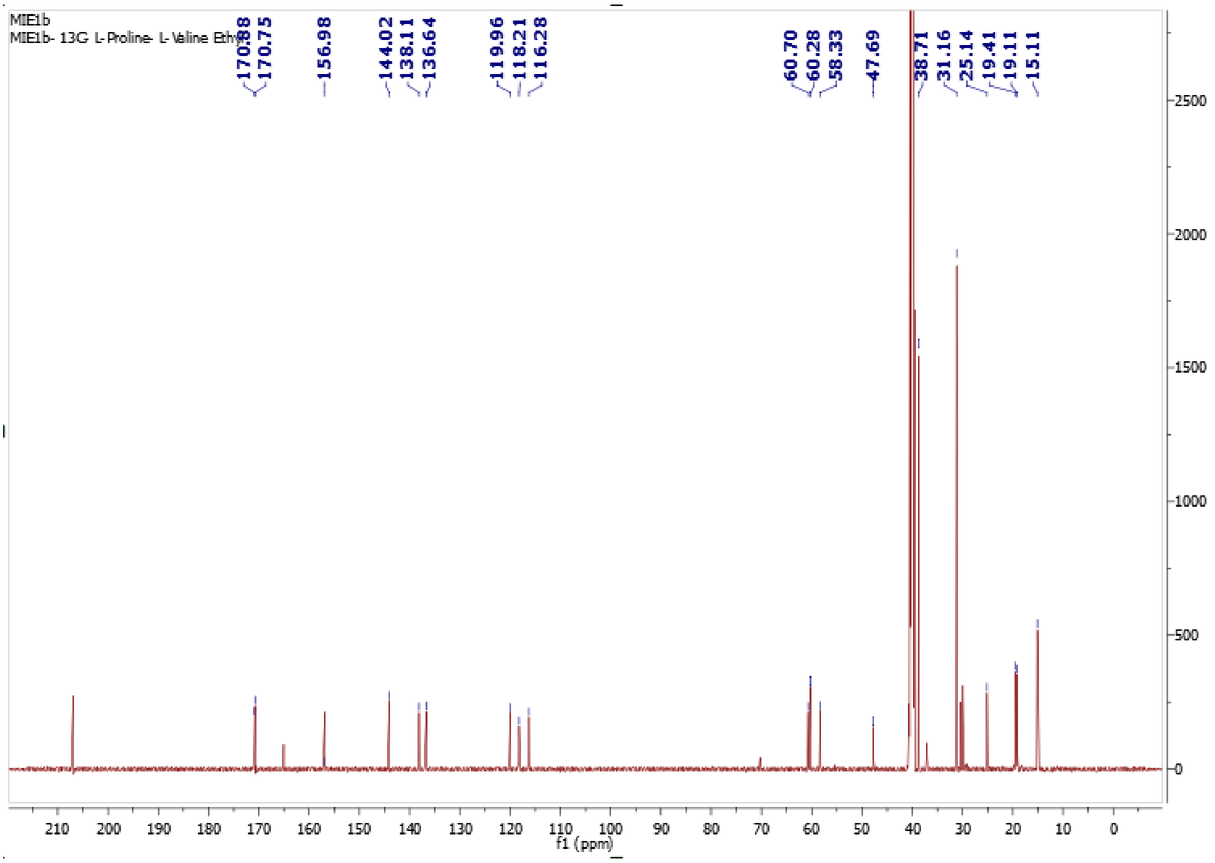
MIE1b
MIE1b- 13C L-Proline- L-Valine Ethyl
170.88
170.75
156.98
144.02
138.11
136.64
119.96
118.21
116.28
60.70
60.28
58.33
47.69
38.71
31.16
25.14
19.41
19.11
15.11
f1 (ppm)

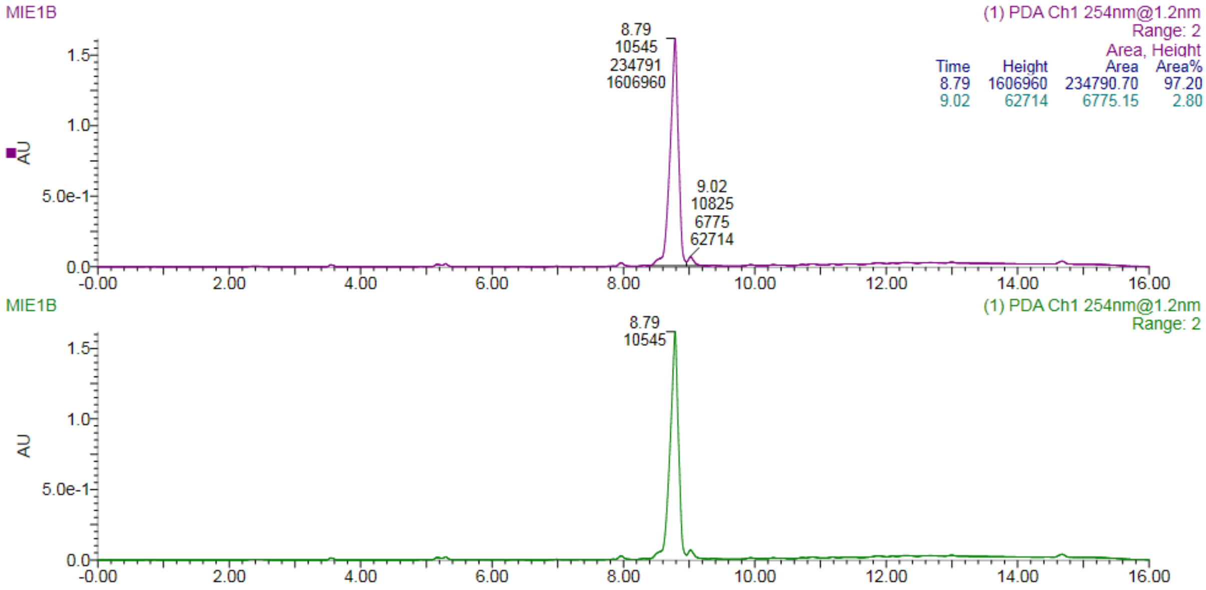
MIE1B
(1) PDA Ch1 254nm@1.2nm
Range: 2
Area, Height
Time Height Area Area%
8.79 1606960 234790.70 97.20
9.02 62714 6775.15 2.80
8.79
10545
234791
1606960
9.02
10825
6775
62714
AU
MIE1B
(1) PDA Ch1 254nm@1.2nm
Range: 2
8.79
10545
AU

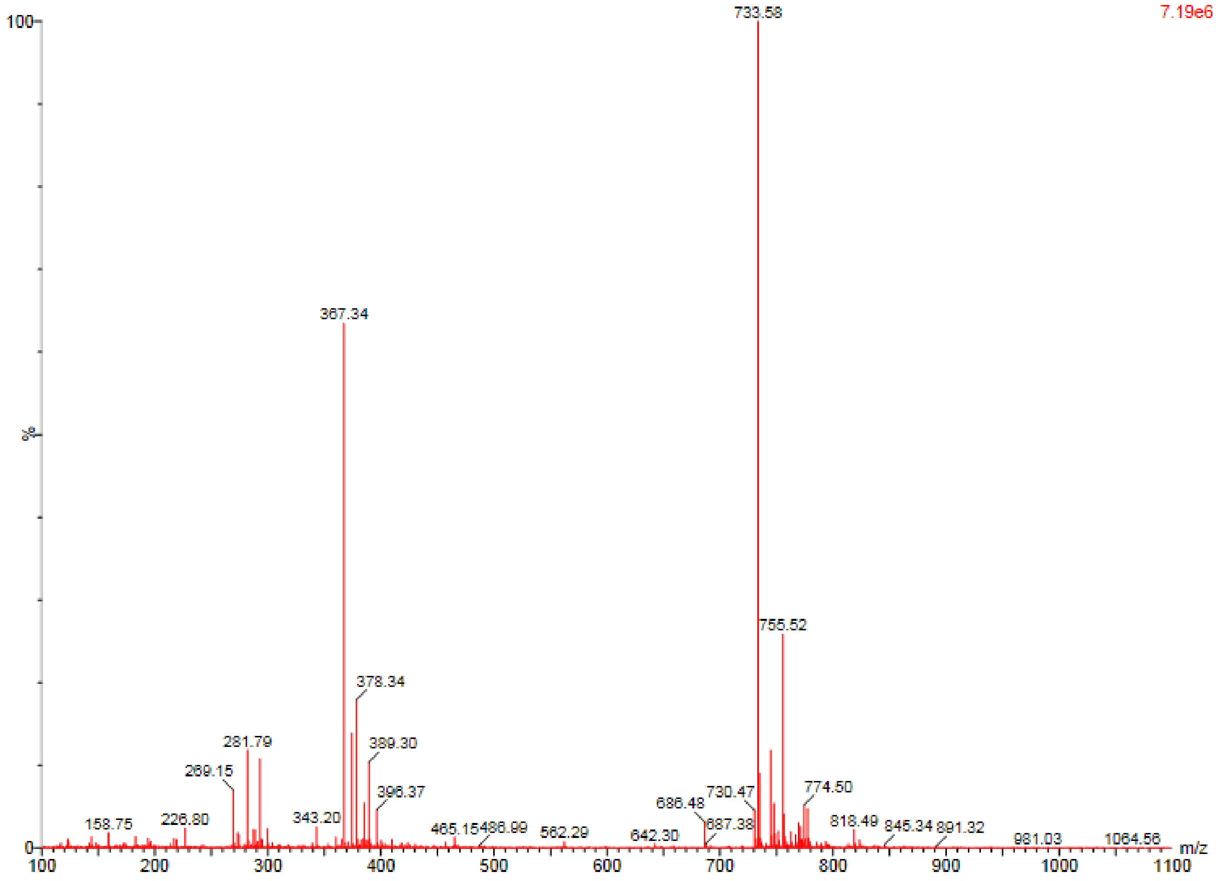

733.58
7.19e6
367.34
755.52
378.34
281.79
389.30
269.15
774.50
396.37
730.47
686.48
343.20
158.75
226.80
687.38
818.49
465.15
486.99
845.34
891.32
562.29
642.30
981.03
1064.56
m/z
%
100
0

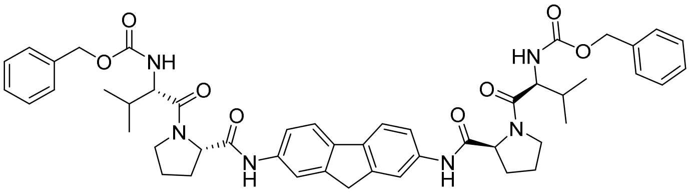

4

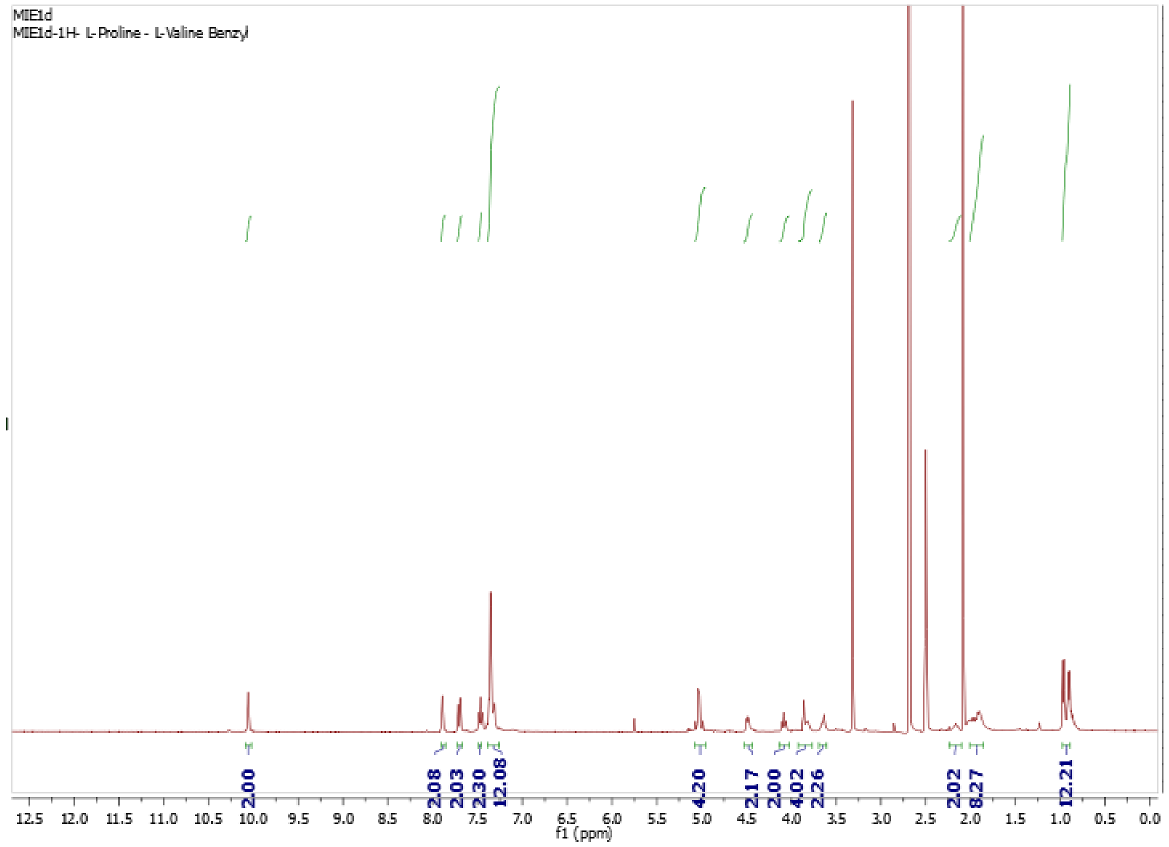
MIE1d
MIE1d-1H- L-Proline - L-Valine Benzyl
2.00
2.08
2.03
2.30
12.08
4.20
2.17
2.00
4.02
2.26
2.02
8.27
12.21
f1 (ppm)

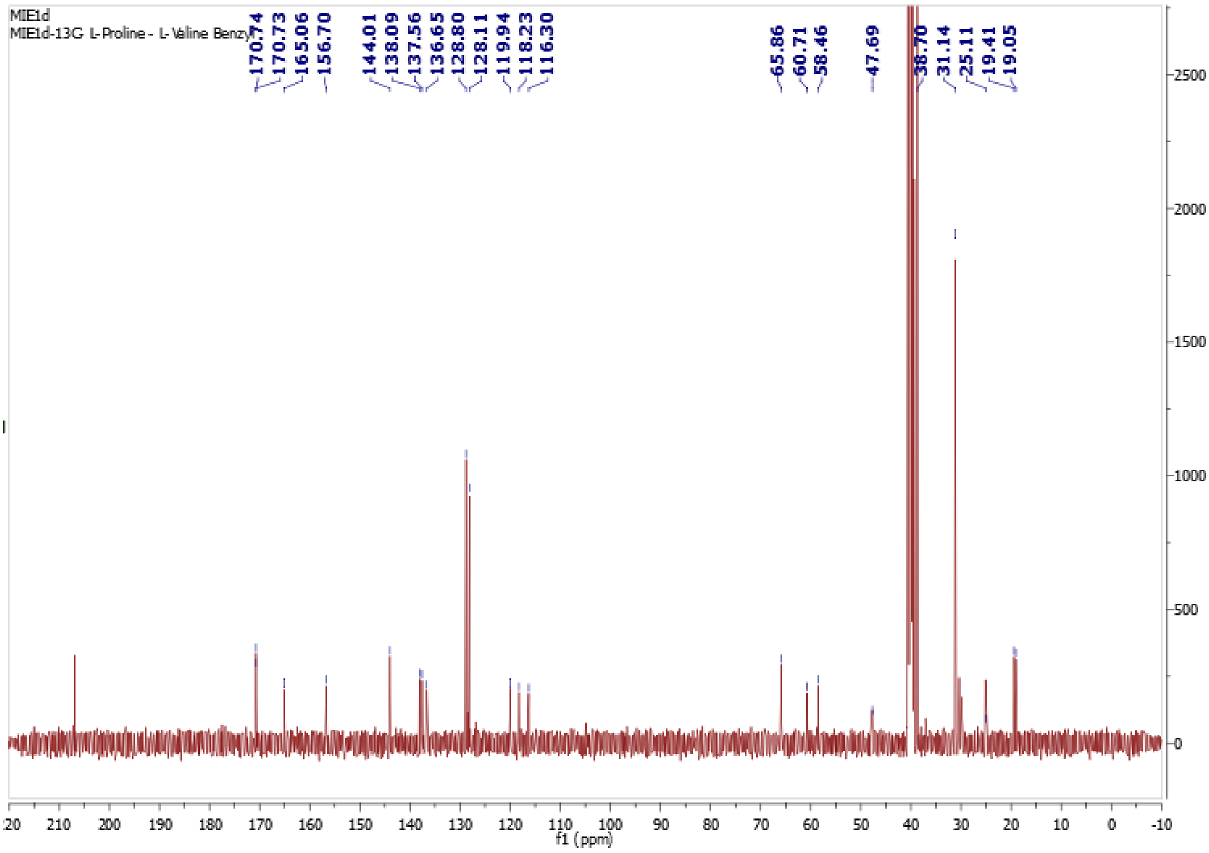
MIE1d
MIE1d-13C L-Proline - L-Valine Benzyl
170.74
170.73
165.06
156.70
144.01
138.09
137.56
136.65
128.80
128.11
119.94
118.23
116.30
65.86
60.71
58.46
47.69
38.70
31.14
25.11
19.41
19.05
f1 (ppm)

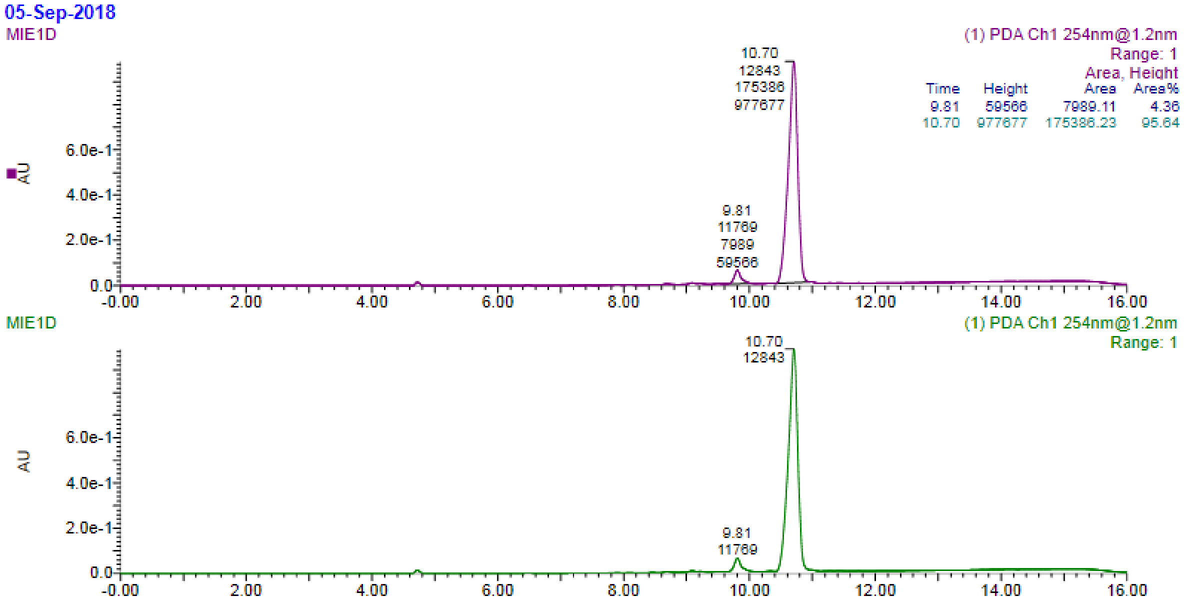
05-Sep-2018
MIE1D
(1) PDA Ch1 254nm@1.2nm
Range: 1
Area, Height
Time Height Area Area%
9.81 59566 7989.11 4.36
10.70 977677 175386.23 95.64
10.70
12843
175386
977677
9.81
11769
7989
59566
AU
MIE1D
(1) PDA Ch1 254nm@1.2nm
Range: 1
10.70
12843
9.81
11769
AU

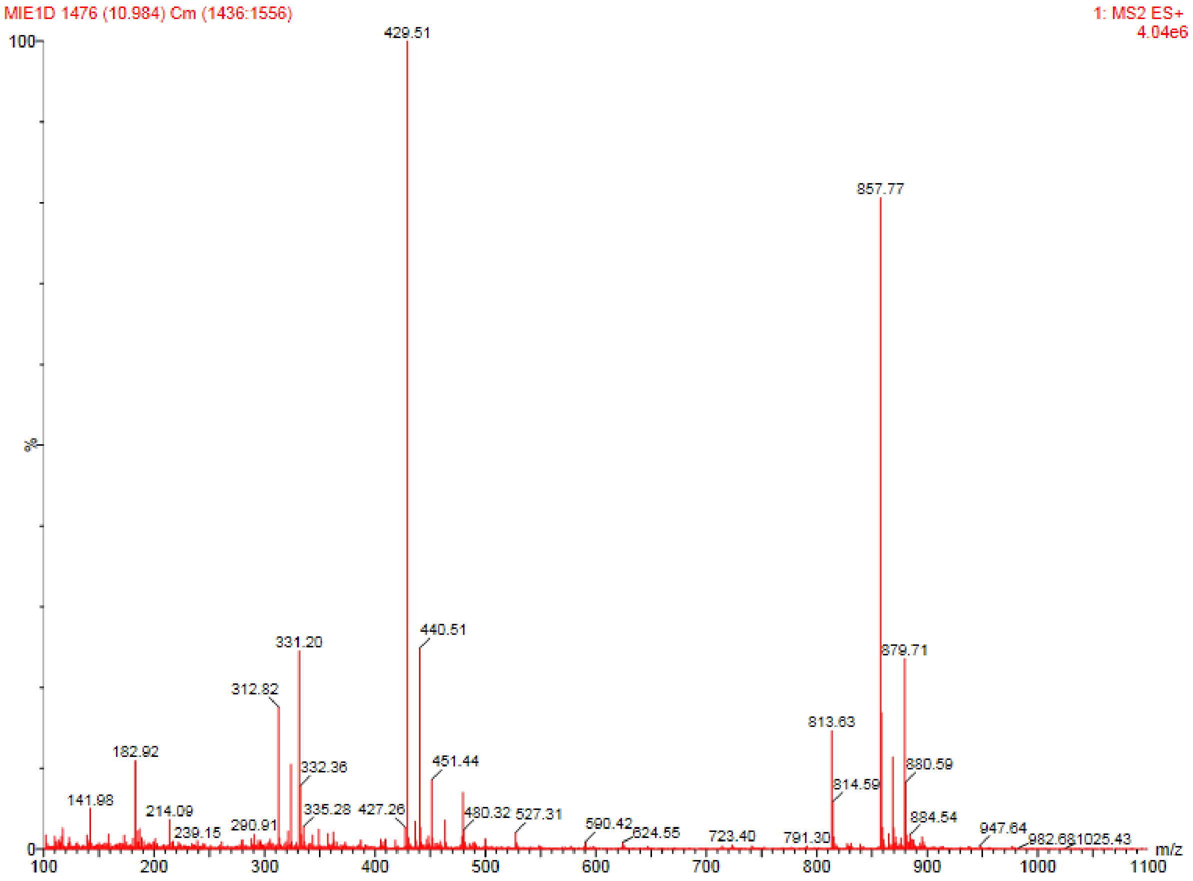

MIE1D 1476 (10.984) Cm (1436:1556)
1: MS2 ES+
4.04e6
100
%
0
429.51
857.77
440.51
331.20
879.71
312.82
813.63
182.92
451.44
332.36
880.59
814.59
141.98
214.09
335.28
427.26
480.32
527.31
884.54
290.91
239.15
590.42
624.55
723.40
791.30
947.64
982.68
1025.43
m/z
100
200
300
400
500
600
700
800
900
1000
1100

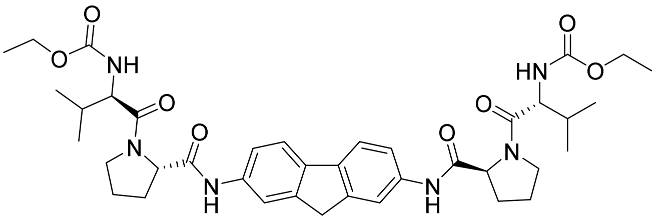

6

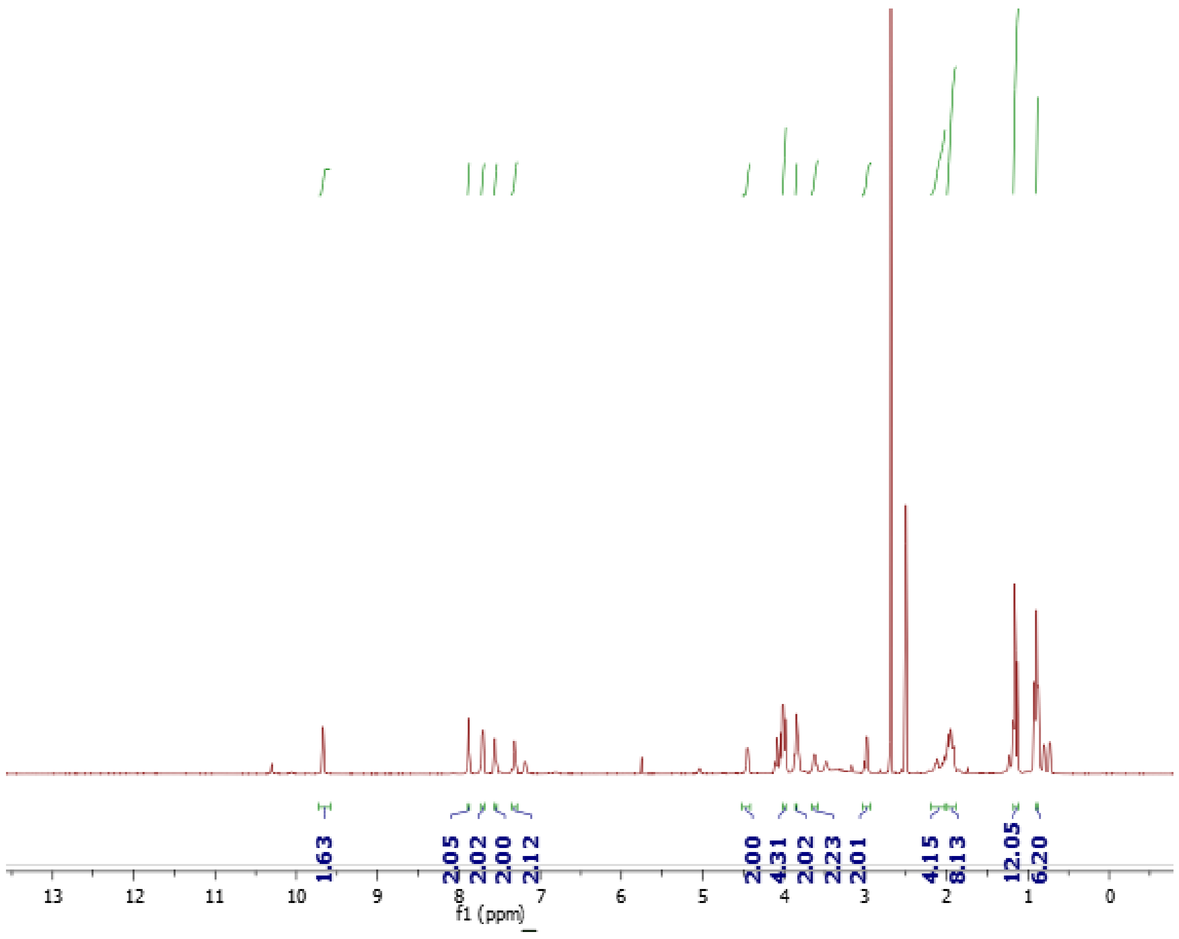

1.63
2.05
2.02
2.00
2.12
2.00
4.31
2.02
2.23
2.01
4.15
8.13
12.05
6.20
13
12
11
10
9
8
7
6
5
4
3
2
1
0
f1 (ppm)

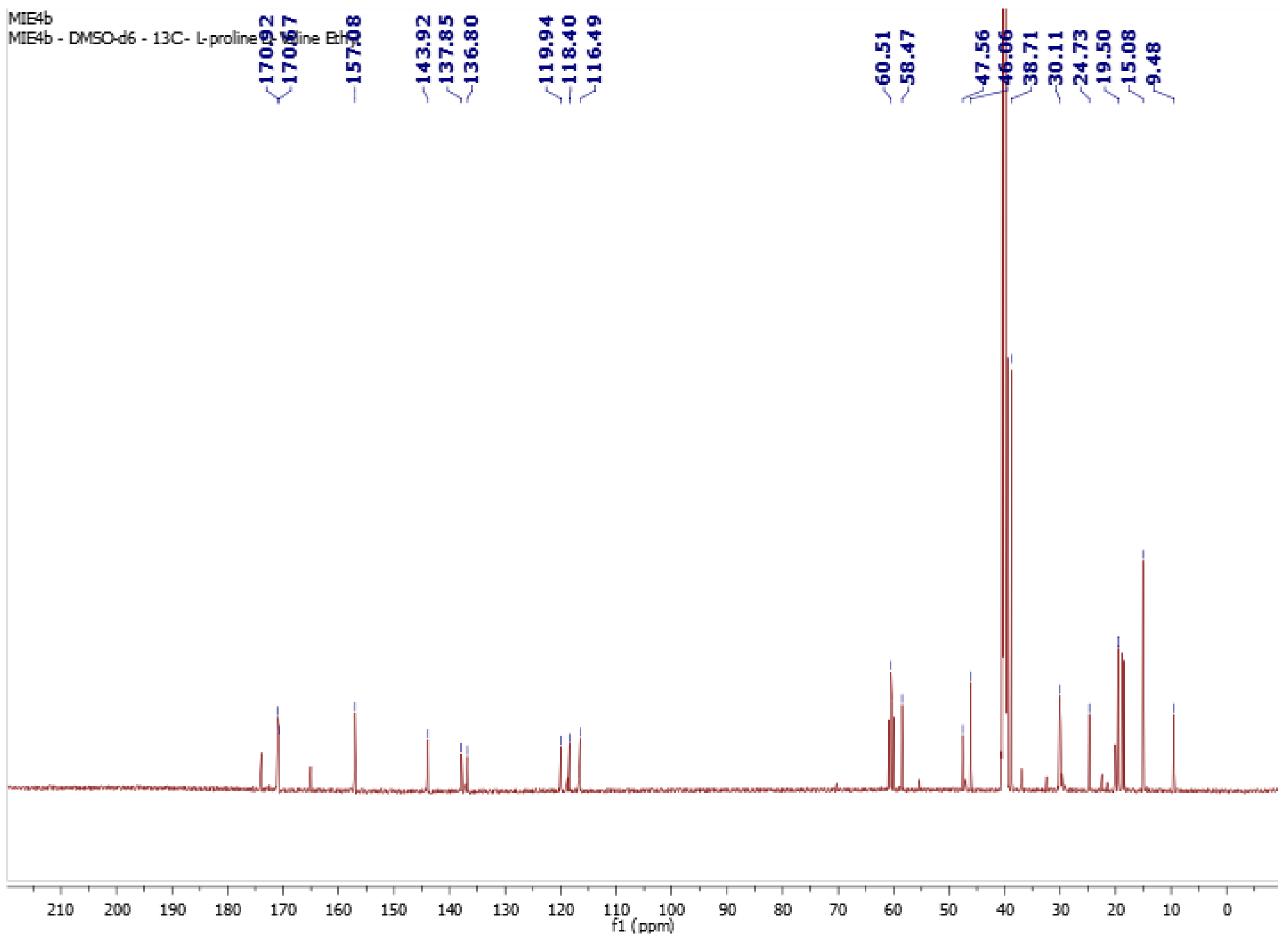
MIE4b
MIE4b - DMSO-d6 - 13C- L-proline-L-Valine Eth
170.92
170.67
157.08
143.92
137.85
136.80
119.94
118.40
116.49
60.51
58.47
47.56
46.06
38.71
30.11
24.73
19.50
15.08
9.48
f1 (ppm)

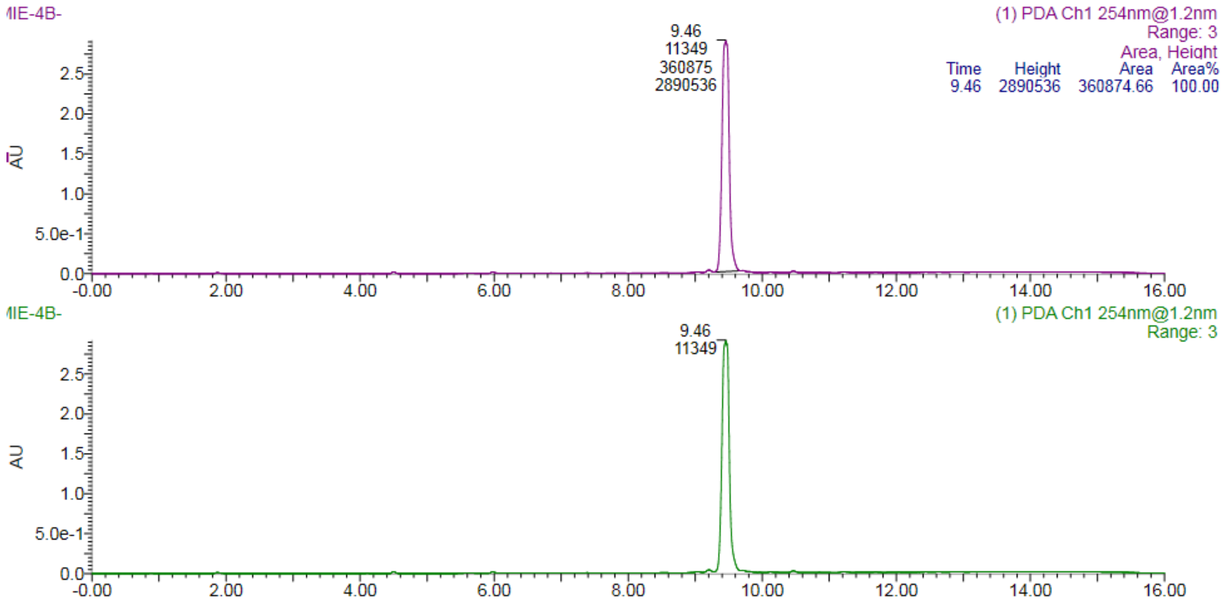
MIE-4B-
(1) PDA Ch1 254nm@1.2nm
Range: 3
Area, Height
Time Height Area Area%
9.46 2890536 360874.66 100.00
9.46
11349
360875
2890536
AU
MIE-4B-
(1) PDA Ch1 254nm@1.2nm
Range: 3
9.46
11349
AU

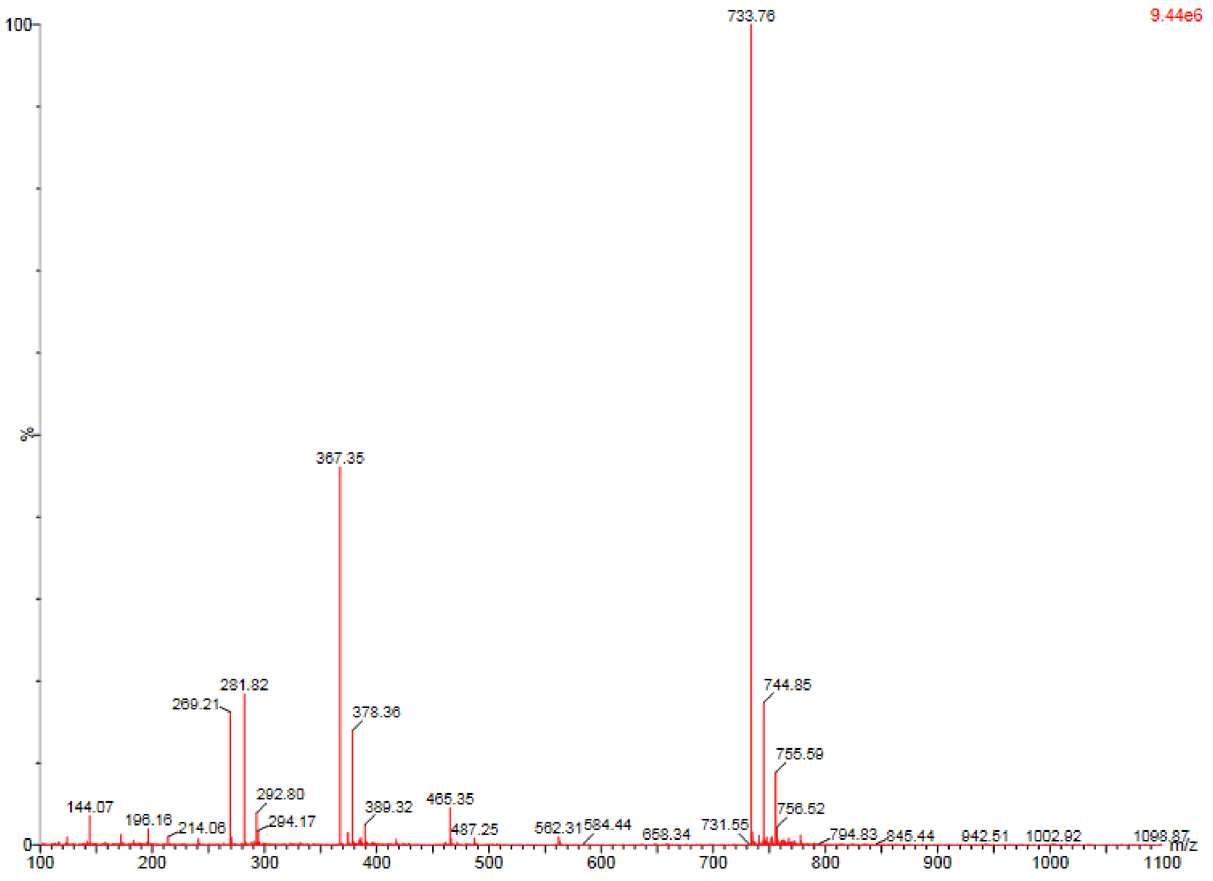

733.76
9.44e6
100
%
0
367.35
281.82
269.21
378.36
744.85
755.59
292.80
465.35
144.07
389.32
756.52
196.16
294.17
214.06
487.25
562.31
584.44
658.34
731.55
794.83
845.44
942.51
1002.92
1098.87
m/z
100
200
300
400
500
600
700
800
900
1000
1100

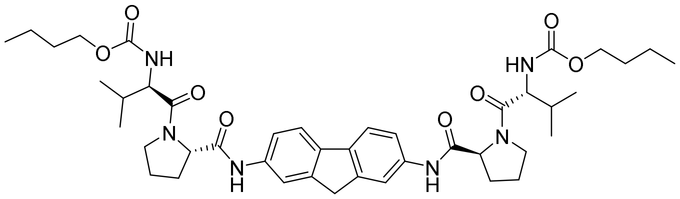

7

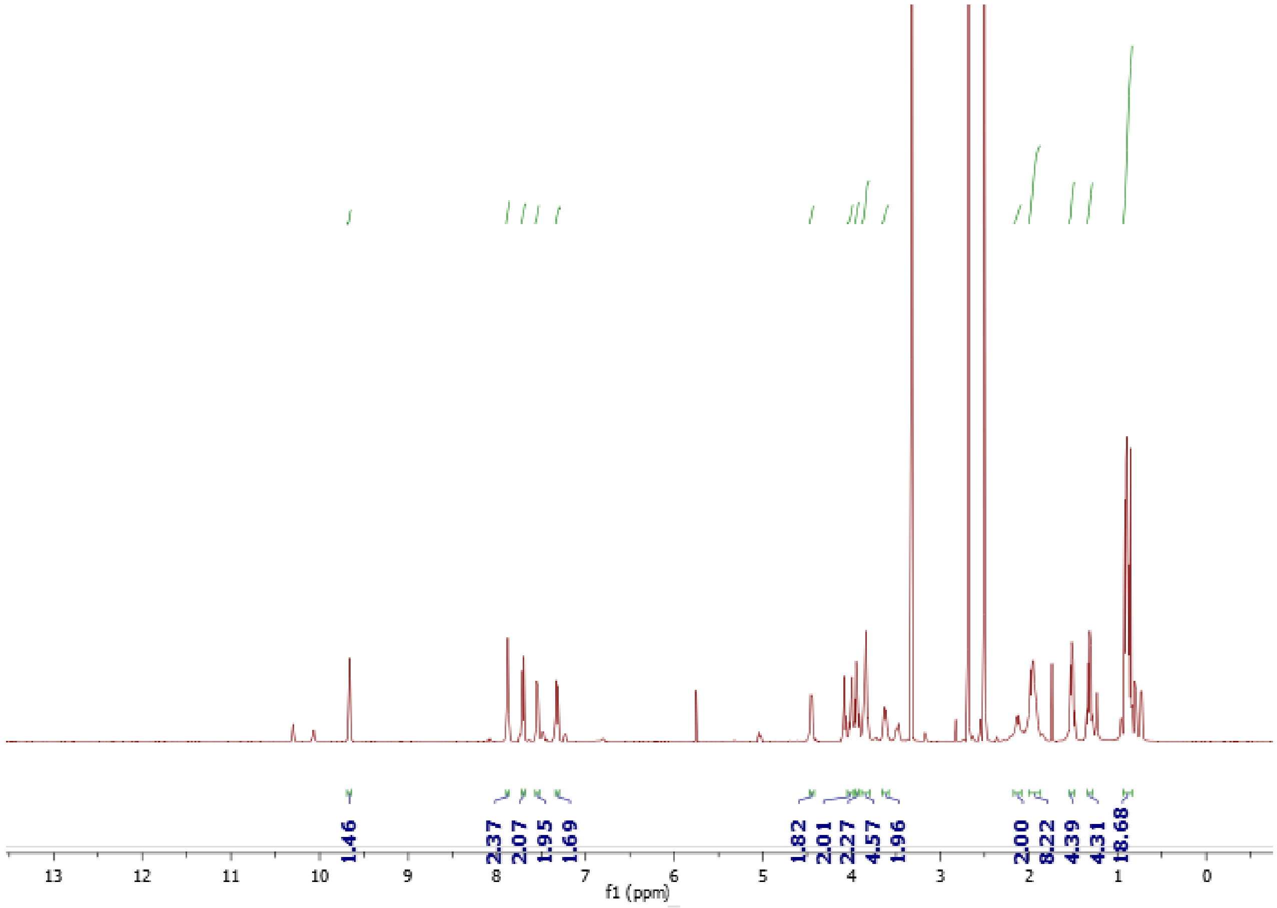

1.46
2.37
2.07
1.95
1.69
1.82
2.01
2.27
4.57
1.96
2.00
8.22
4.39
4.31
18.68
13
12
11
10
9
8
7
6
5
4
3
2
1
0
f1 (ppm)

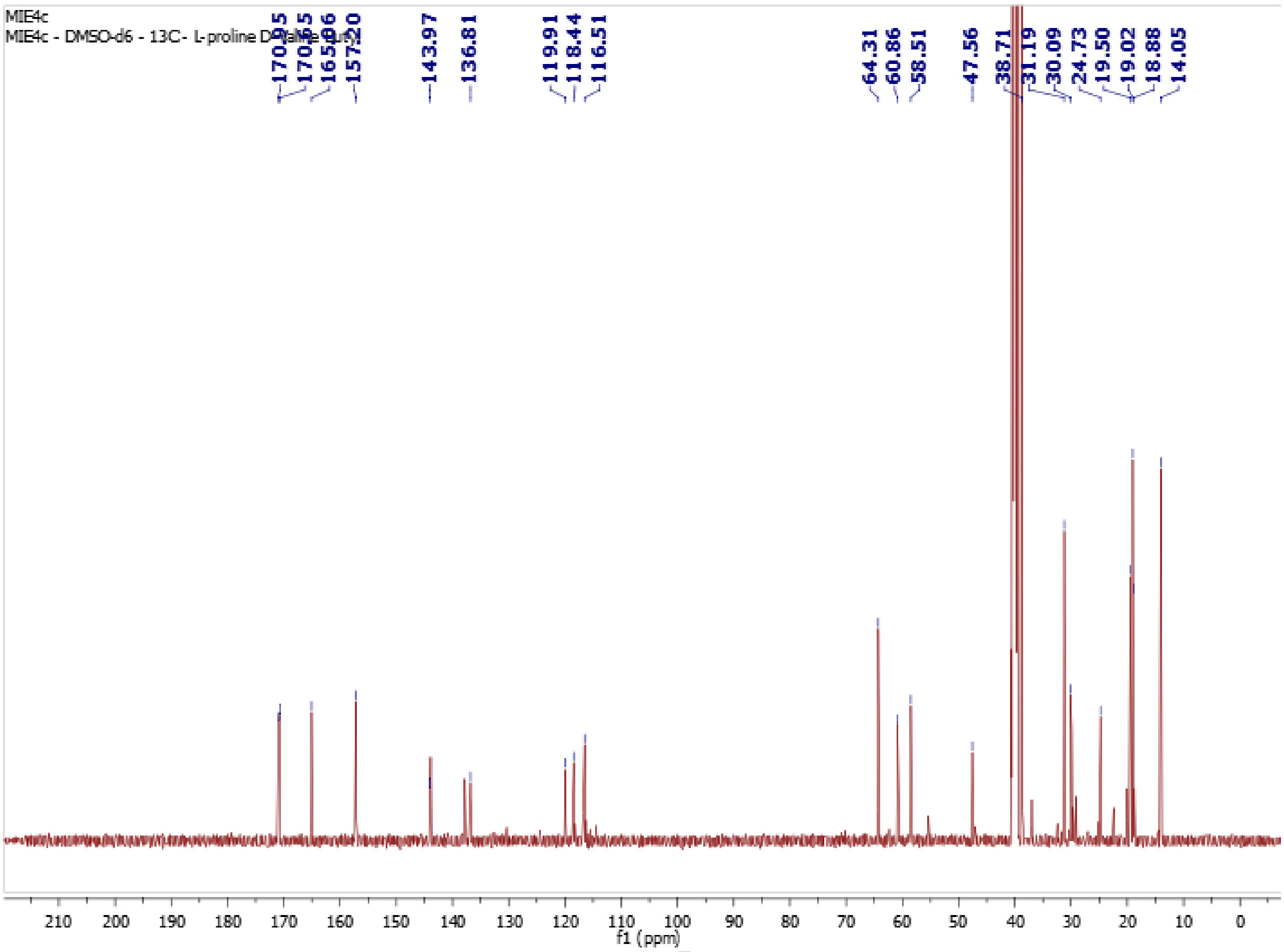
MIE4c
MIE4c - DMSO-d6 - 13C- L-proline D-valine butyl
170.95
170.55
165.06
157.20
143.97
136.81
119.91
118.44
116.51
64.31
60.86
58.51
47.56
38.71
31.19
30.09
24.73
19.50
19.02
18.88
14.05
f1 (ppm)

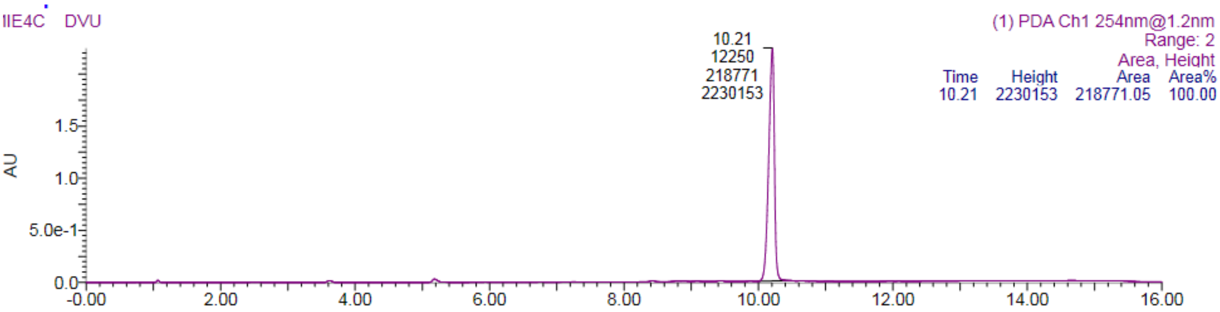
IIE4C DVU
(1) PDA Ch1 254nm@1.2nm
Range: 2
Area, Height
10.21
12250
218771
2230153
Time Height Area Area%
10.21 2230153 218771.05 100.00
AU

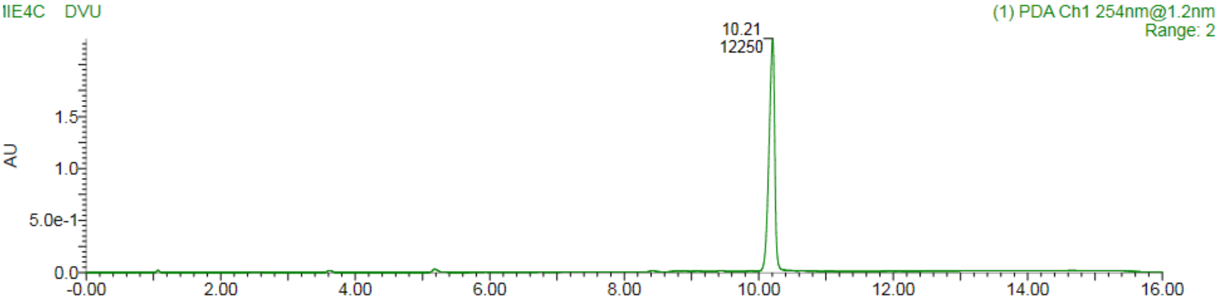
IIE4C DVU
(1) PDA Ch1 254nm@1.2nm
Range: 2
10.21
12250
AU

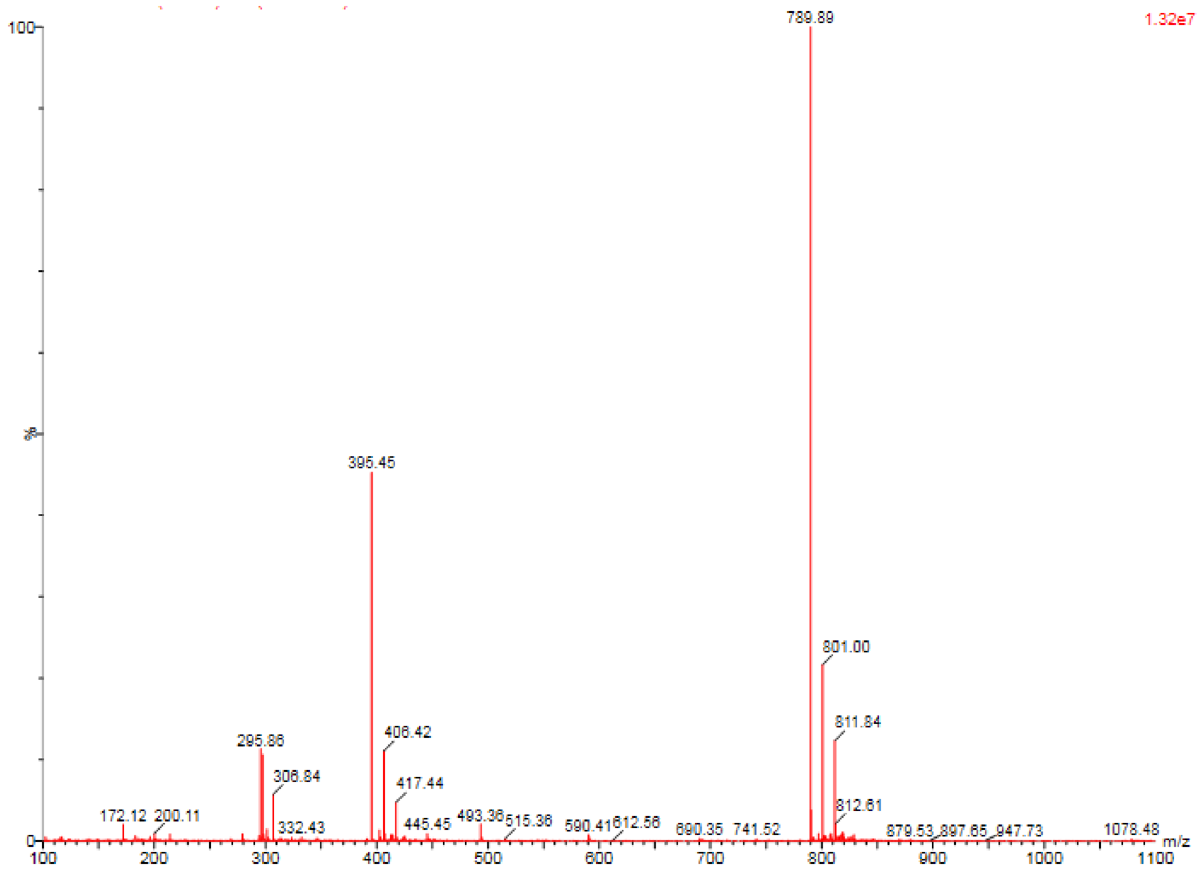

789.89
1.32e7
100
%
0
395.45
801.00
811.84
406.42
295.86
306.84
417.44
812.61
172.12
200.11
332.43
445.45
493.36
515.36
590.41
612.56
690.35
741.52
879.53
897.65
947.73
1078.48
m/z
100
200
300
400
500
600
700
800
900
1000
1100

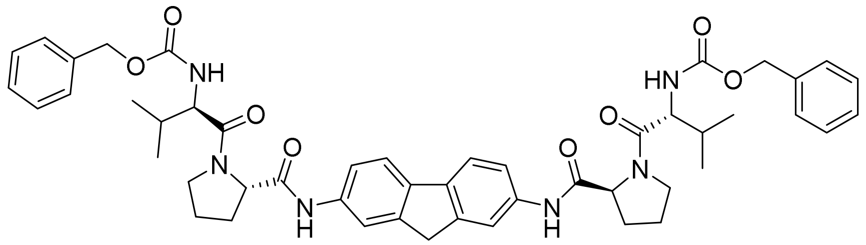

8

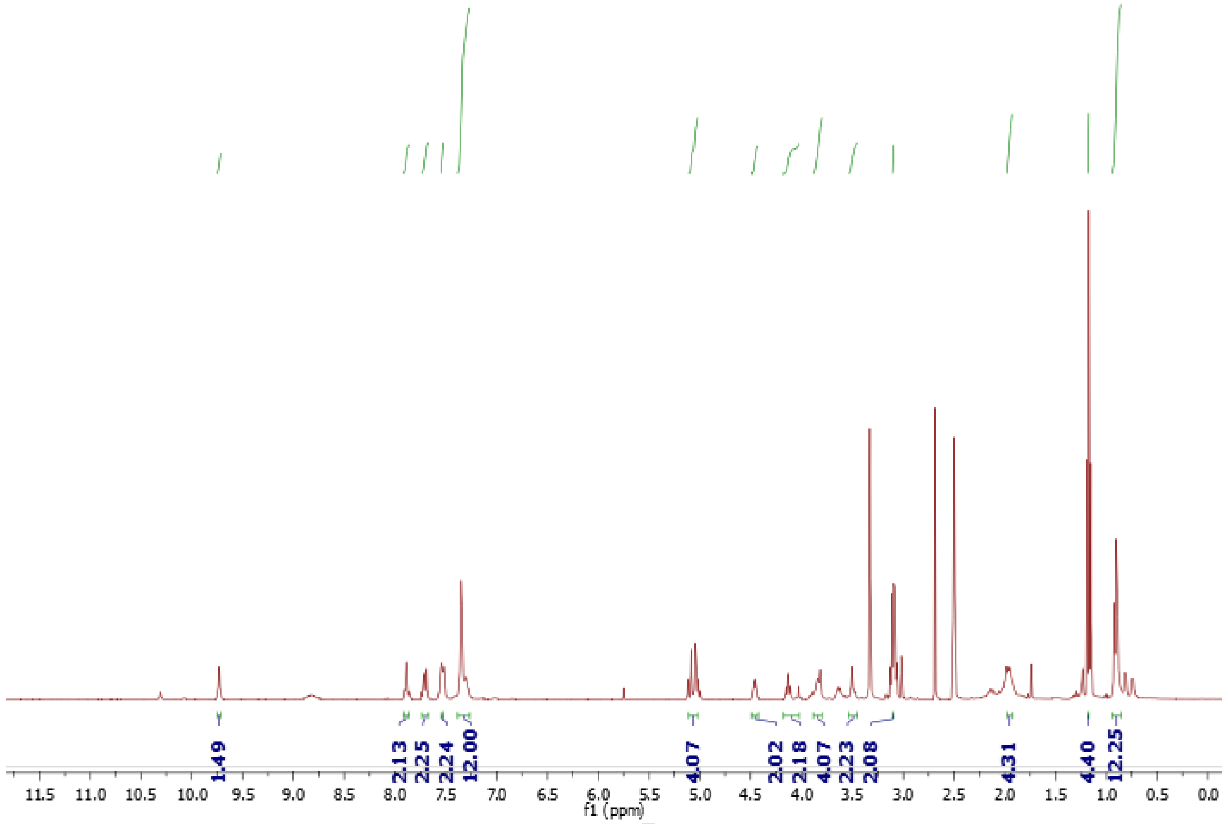
1.49
2.13
2.25
2.24
12.00
4.07
2.02
2.18
4.07
2.23
2.08
4.31
4.40
12.25
11.5 11.0 10.5 10.0 9.5 9.0 8.5 8.0 7.5 7.0 6.5 6.0 5.5 5.0 4.5 4.0 3.5 3.0 2.5 2.0 1.5 1.0 0.5 0.0
f1 (ppm)

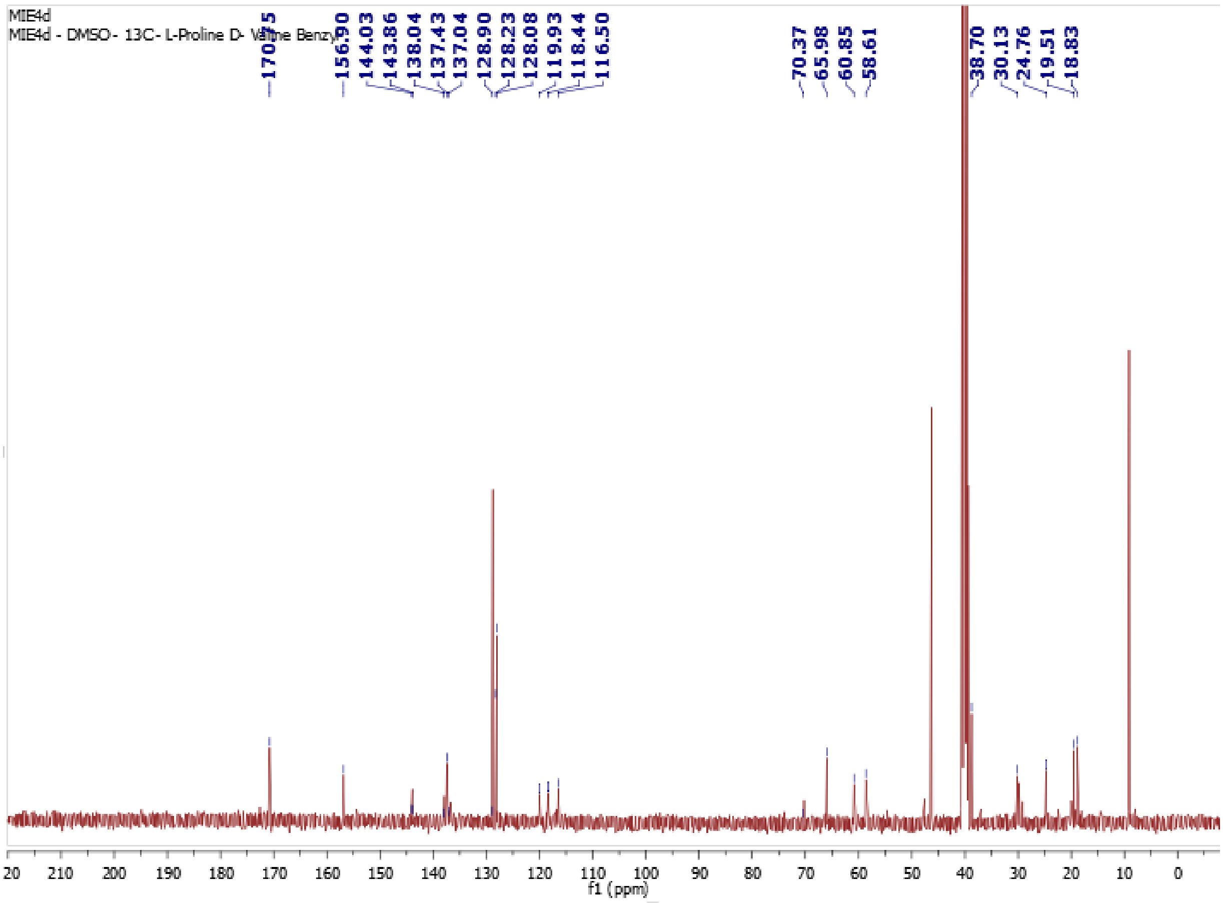
MIE4d
MIE4d - DMSO - 13C - L-Proline D- Valine Benzyl
170.75
156.90
144.03
143.86
138.04
137.43
137.04
128.90
128.23
128.08
119.93
118.44
116.50
70.37
65.98
60.85
58.61
38.70
30.13
24.76
19.51
18.83
f1 (ppm)

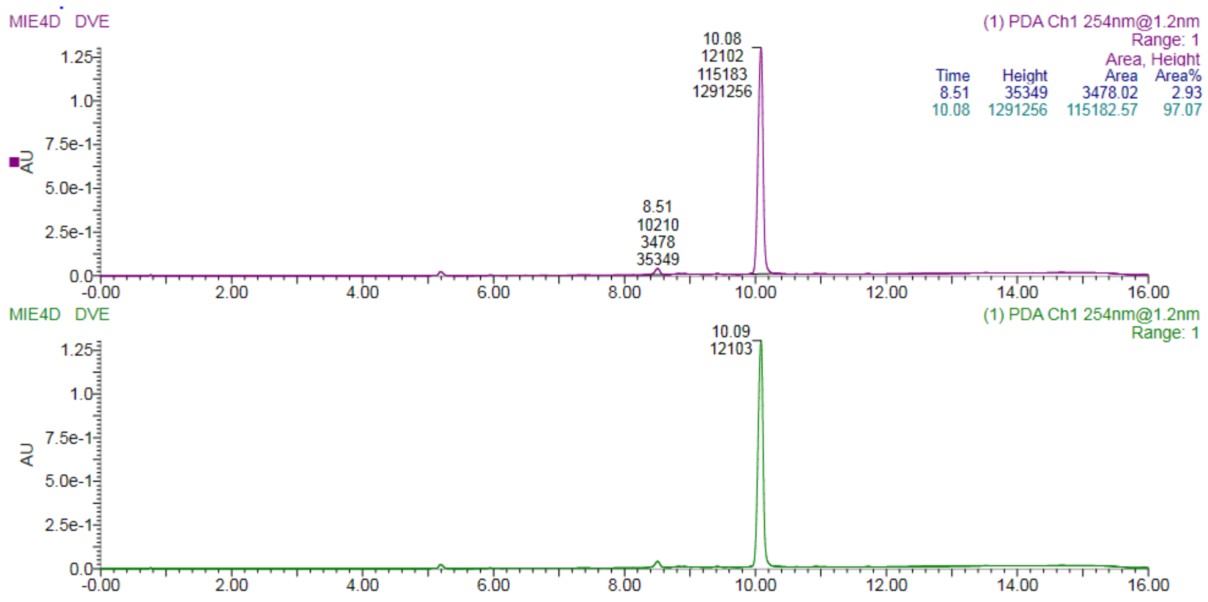
MIE4D DVE
(1) PDA Ch1 254nm@1.2nm
Range: 1
Area, Height
Time Height Area Area%
8.51 35349 3478.02 2.93
10.08 1291256 115182.57 97.07
10.08
12102
115183
1291256
8.51
10210
3478
35349
AU
MIE4D DVE
(1) PDA Ch1 254nm@1.2nm
Range: 1
10.09
12103
AU

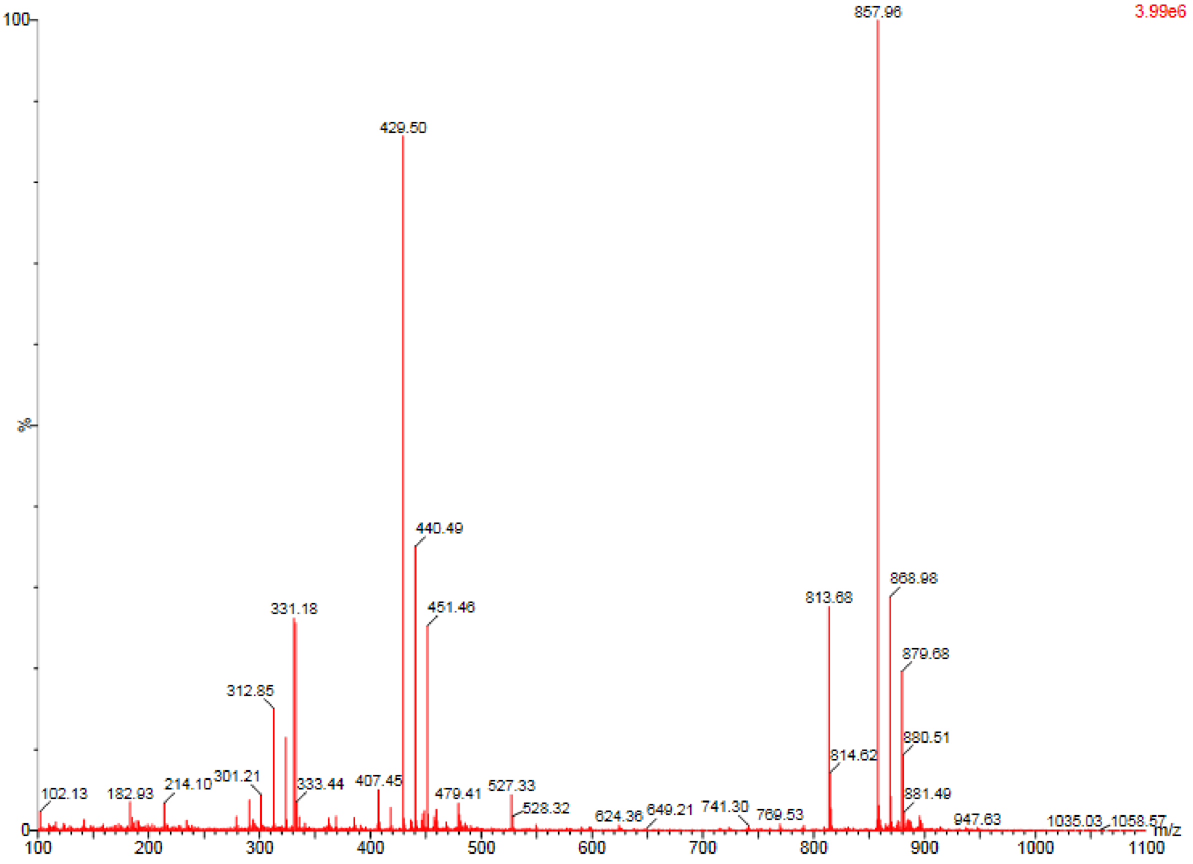

100
%
0
3.99e6
857.96
429.50
440.49
451.46
331.18
312.85
813.68
868.98
879.68
880.51
814.62
881.49
102.13
182.93
214.10
301.21
333.44
407.45
479.41
527.33
528.32
624.36
649.21
741.30
769.53
947.63
1035.03
1058.57
m/z
100
200
300
400
500
600
700
800
900
1000
1100

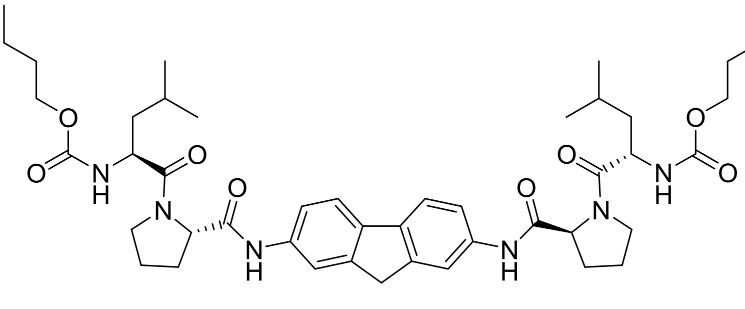

11

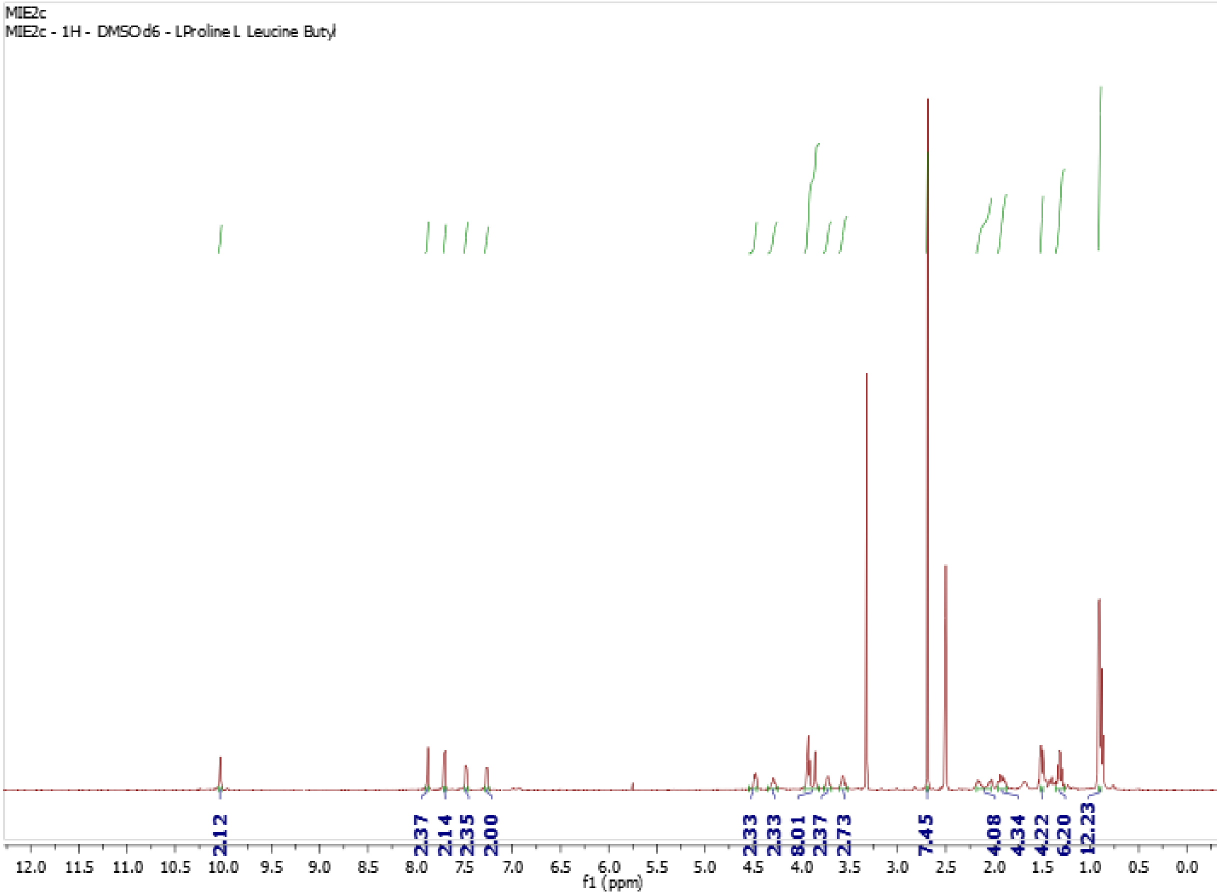
MIE2c
MIE2c - 1H - DMSO d6 - LProline L Leucine Butyl
2.12
2.37
2.14
2.35
2.00
2.33
2.33
8.01
2.37
2.73
7.45
4.08
4.34
4.22
6.20
12.23
12.0 11.5 11.0 10.5 10.0 9.5 9.0 8.5 8.0 7.5 7.0 6.5 6.0 5.5 5.0 4.5 4.0 3.5 3.0 2.5 2.0 1.5 1.0 0.5 0.0
f1 (ppm)

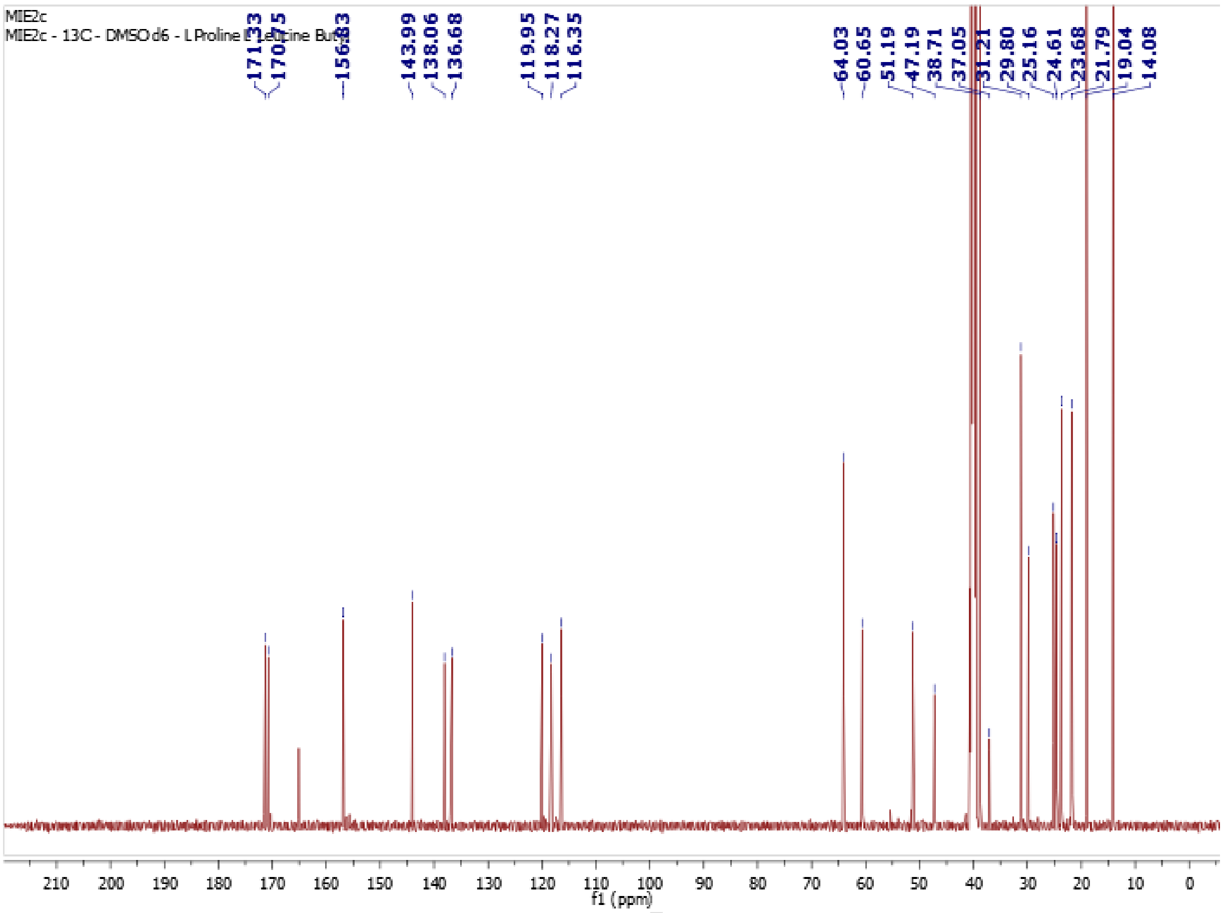
MIE2c
MIE2c - 13C - DMSO d6 - L Proline L Leucine But
171.33
170.75
156.83
143.99
138.06
136.68
119.95
118.27
116.35
64.03
60.65
51.19
47.19
38.71
37.05
31.21
29.80
25.16
24.61
23.68
21.79
19.04
14.08
f1 (ppm)

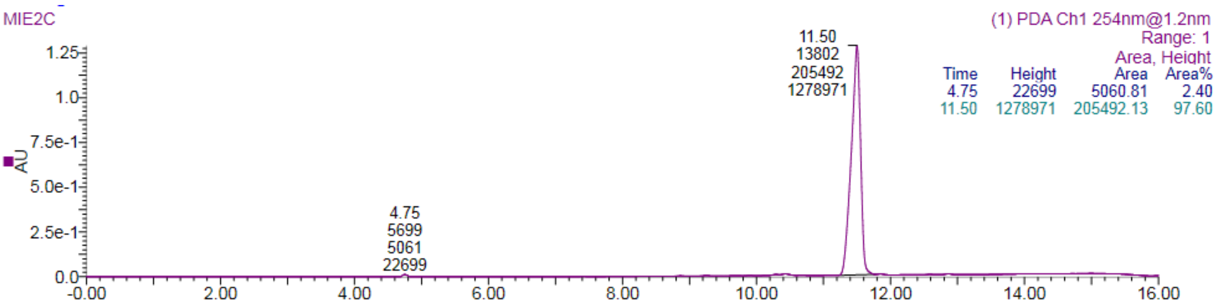
MIE2C
(1) PDA Ch1 254nm@1.2nm
Range: 1
Area, Height
Time Height Area Area%
4.75 22699 5060.81 2.40
11.50 1278971 205492.13 97.60
11.50
13802
205492
1278971
4.75
5699
5061
22699
AU

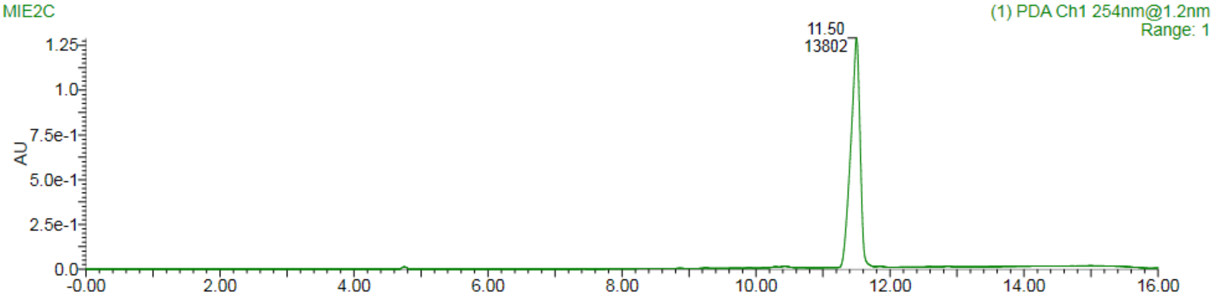
MIE2C
(1) PDA Ch1 254nm@1.2nm
Range: 1
11.50
13802
AU

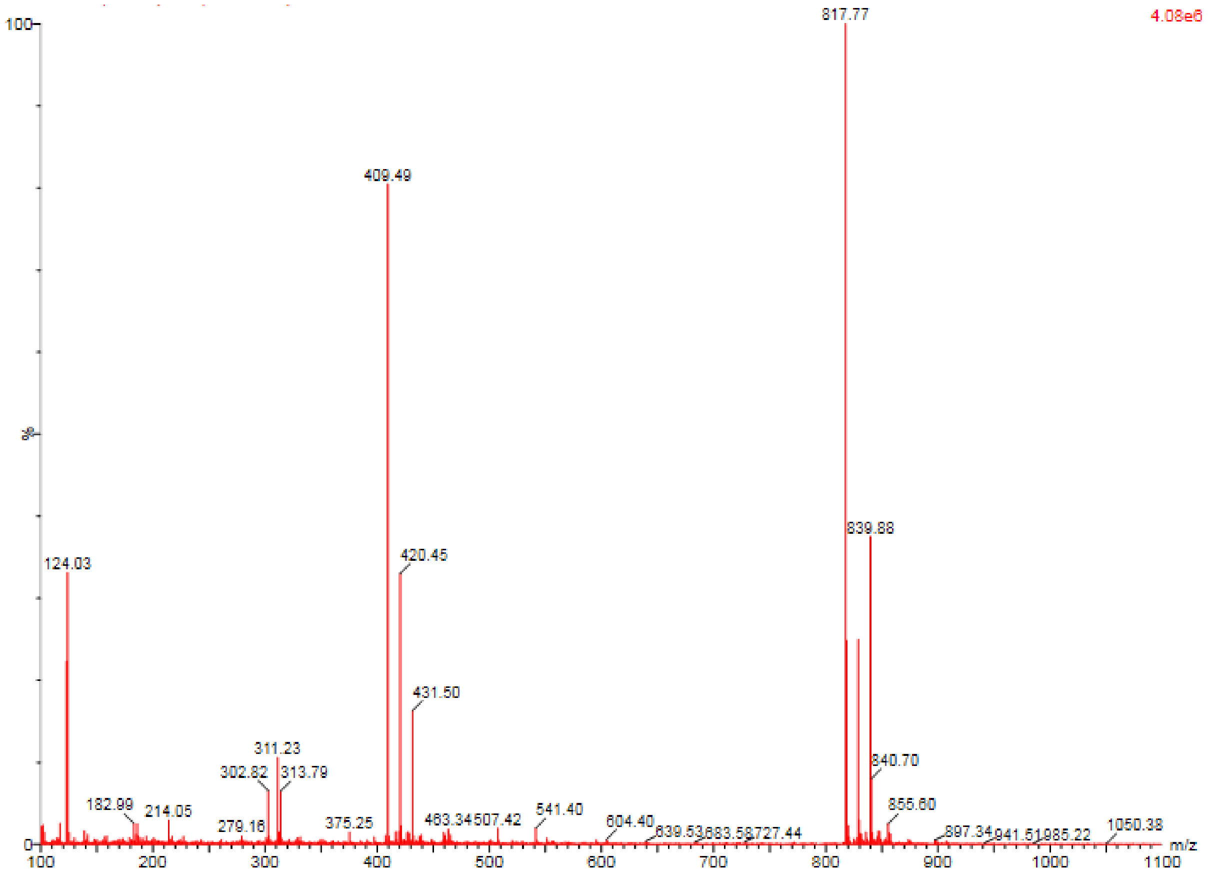

817.77
4.08e6
100
409.49
%
839.88
124.03
420.45
431.50
311.23
302.82
313.79
840.70
182.99
214.05
855.60
279.16
375.25
463.34
507.42
541.40
604.40
639.53
683.58
727.44
897.34
941.51
985.22
1050.38
0
m/z
100
200
300
400
500
600
700
800
900
1000
1100

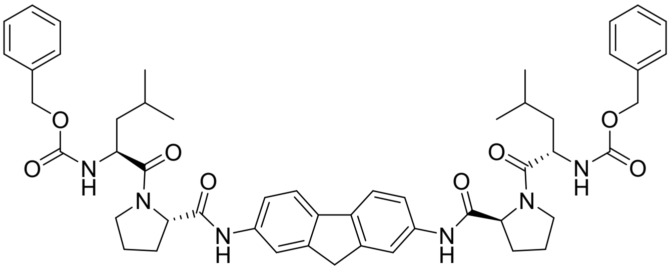

O
NH
N
H

12

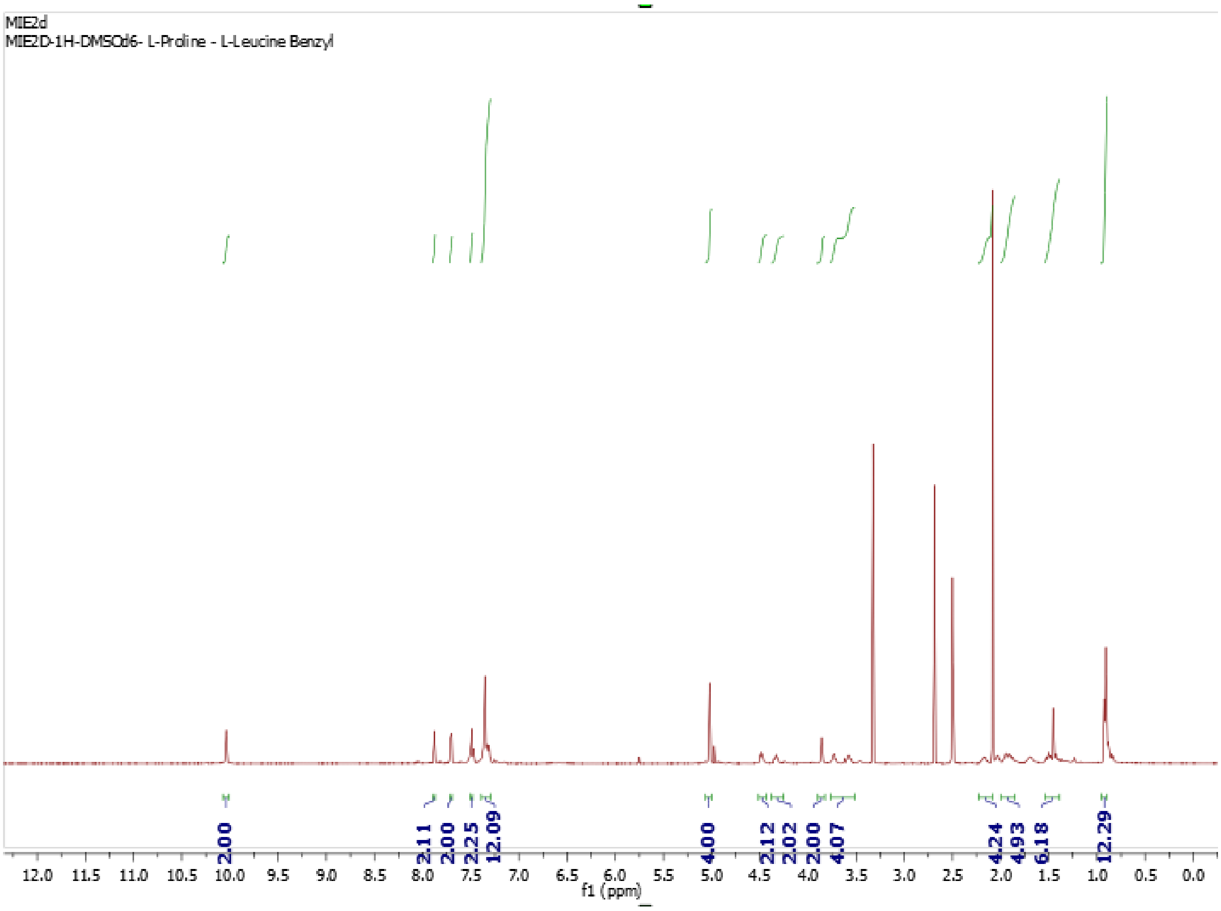

MIE2d
MIE2D-1H-DMSOd6- L-Proline - L-Leucine Benzyl
2.00
2.11
2.00
2.25
12.09
4.00
2.12
2.02
2.00
4.07
4.24
4.93
6.18
12.29
12.0 11.5 11.0 10.5 10.0 9.5 9.0 8.5 8.0 7.5 7.0 6.5 6.0 5.5 5.0 4.5 4.0 3.5 3.0 2.5 2.0 1.5 1.0 0.5 0.0
f1 (ppm)

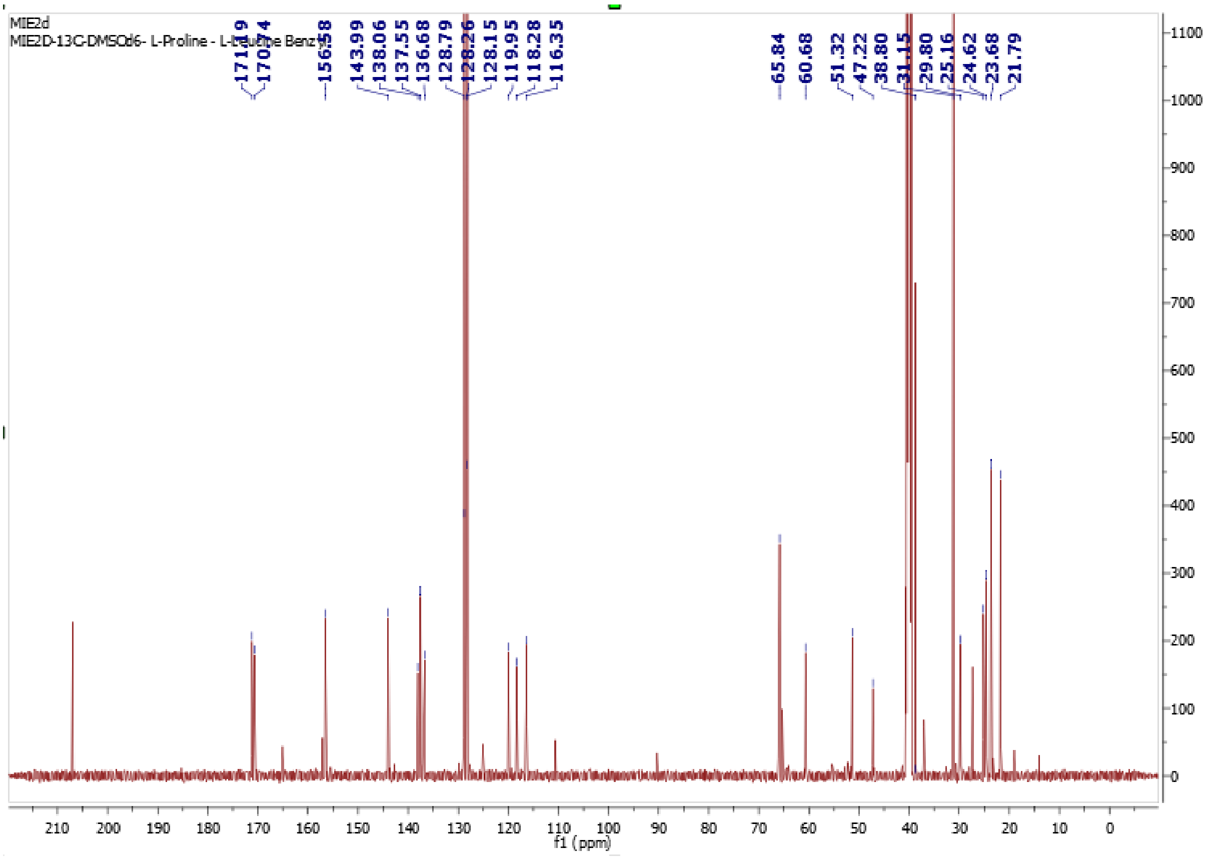

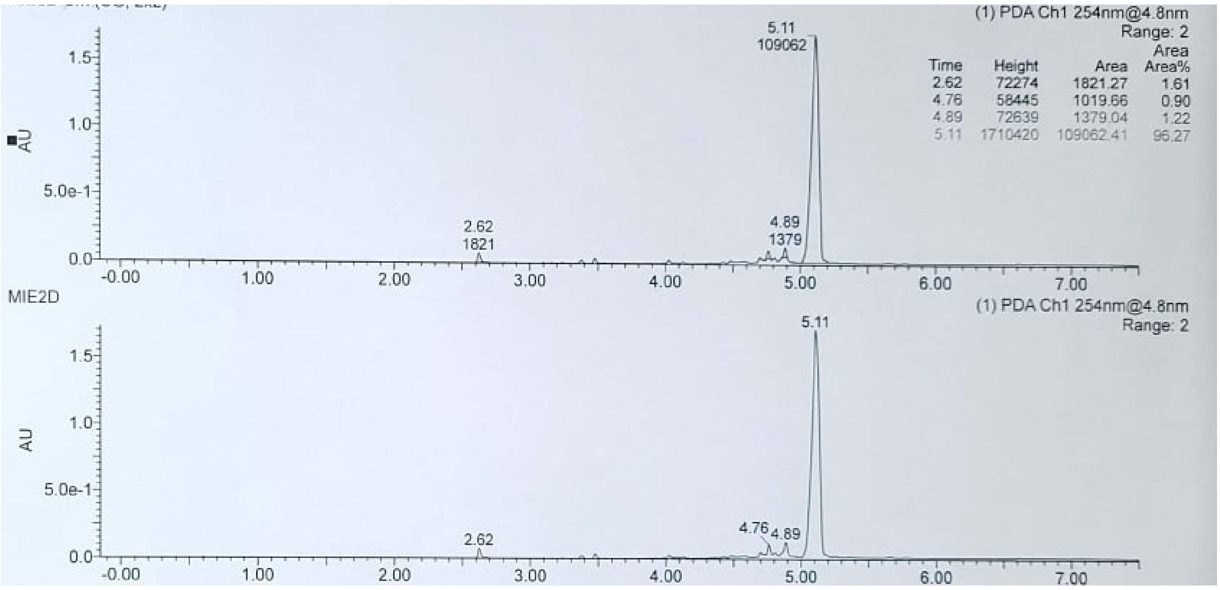

Time	Height	Area	Area Area%
2.62	72274	1821.27	1.61
4.76	58445	1019.66	0.90
4.89	72639	1379.04	1.22
5.11	1710420	109062.41	96.27

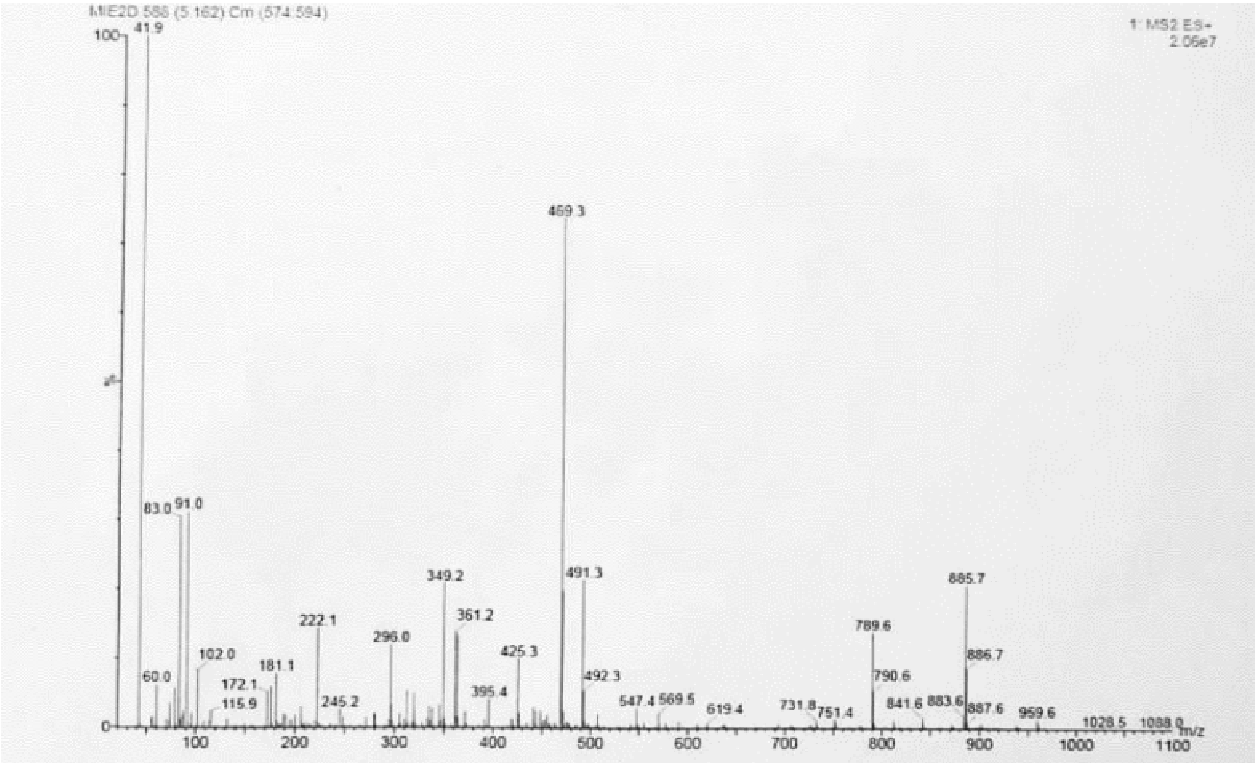

MIE2D 586 (5.162) Cm (574:594)
1: MS2 ES+
2.06e7
100
%
0
41.9
469.3
83.0
91.0
349.2
491.3
885.7
222.1
361.2
296.0
789.6
102.0
425.3
886.7
60.0
181.1
492.3
172.1
790.6
115.9
245.2
395.4
547.4
569.5
619.4
731.8
751.4
841.6
883.6
887.6
959.6
1028.5
1088.0
m/z
100
200
300
400
500
600
700
800
900
1000
1100

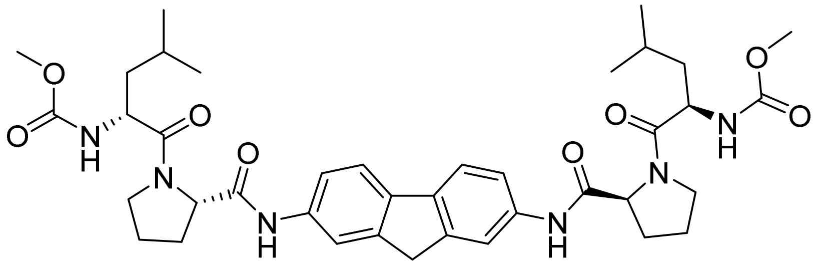

13

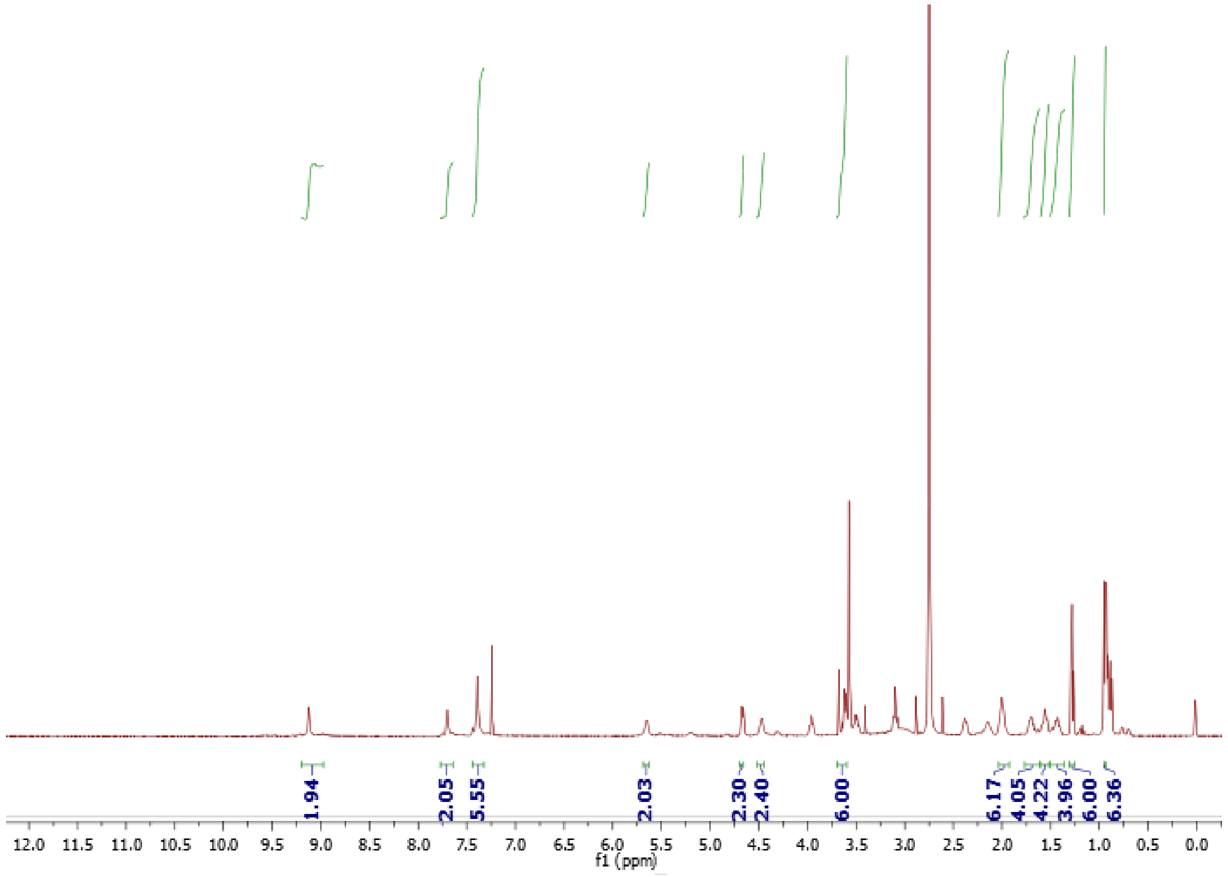

1.94
2.05
5.55
2.03
2.30
2.40
6.00
6.17
4.05
4.22
3.96
6.00
6.36
12.0 11.5 11.0 10.5 10.0 9.5 9.0 8.5 8.0 7.5 7.0 6.5 6.0 5.5 5.0 4.5 4.0 3.5 3.0 2.5 2.0 1.5 1.0 0.5 0.0
f1 (ppm)

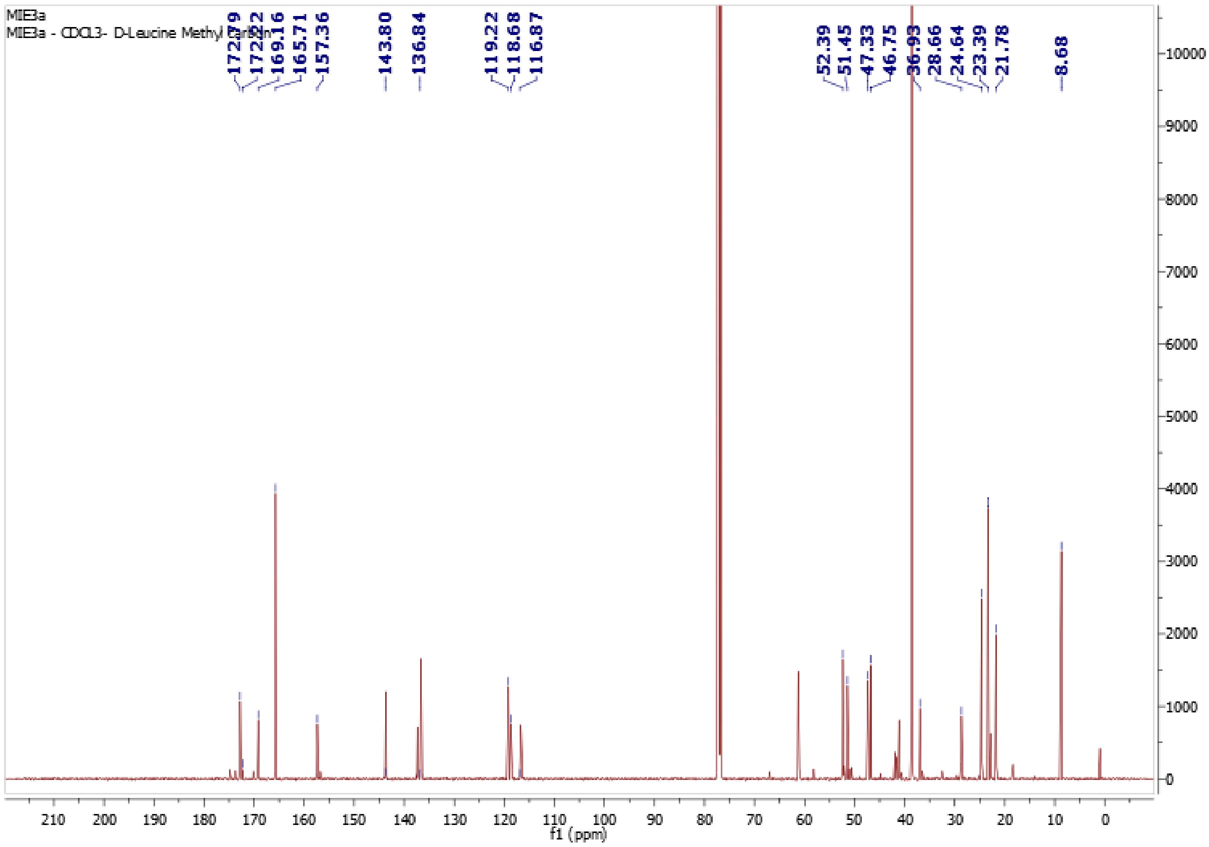
MIE3a
MIE3a - CDCl3- D-Leucine Methyl
172.79
172.22
169.16
165.71
157.36
143.80
136.84
119.22
118.68
116.87
52.39
51.45
47.33
46.75
36.93
28.66
24.64
23.39
21.78
8.68
f1 (ppm)

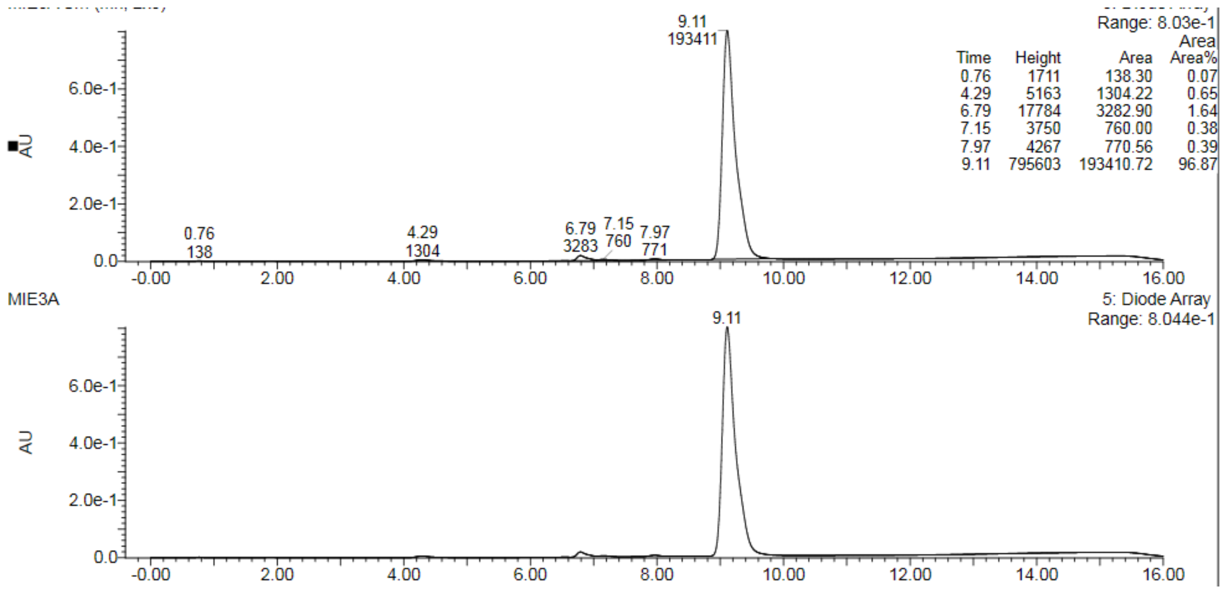
Range: 8.03e-1
Time Height Area Area%
0.76 1711 138.30 0.07
4.29 5163 1304.22 0.65
6.79 17784 3282.90 1.64
7.15 3750 760.00 0.38
7.97 4267 770.56 0.39
9.11 795603 193410.72 96.87
9.11
193411
AU
MIE3A
5: Diode Array
Range: 8.044e-1

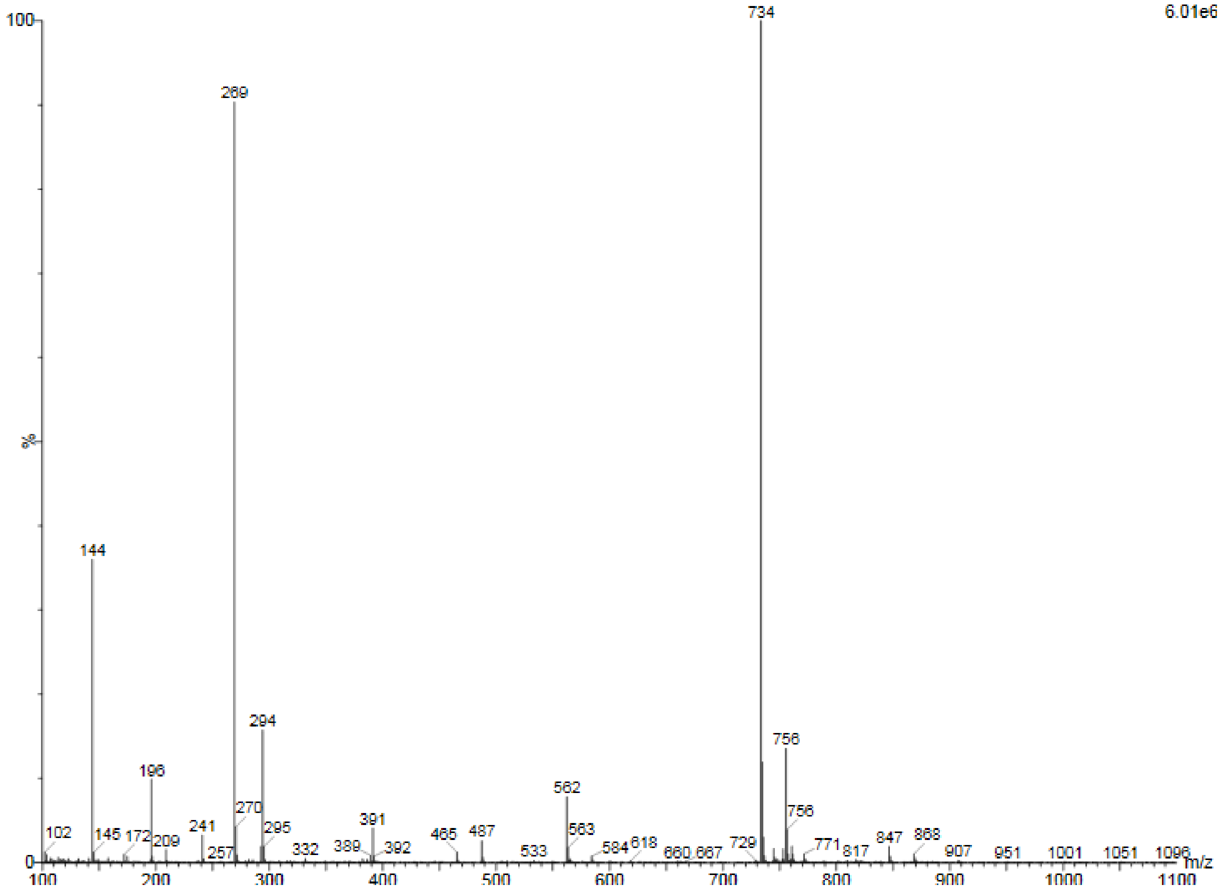

100
734
6.01e6
269
%
144
294
196
756
562
270
758
102
145
172
209
241
257
295
332
389
391
392
465
487
533
563
584
618
660
667
729
771
817
847
868
907
951
1001
1051
1096
m/z
0
100
200
300
400
500
600
700
800
900
1000
1100

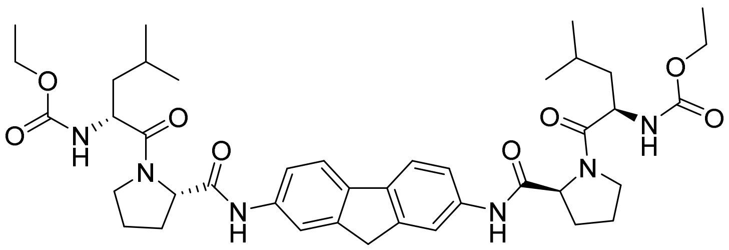

14

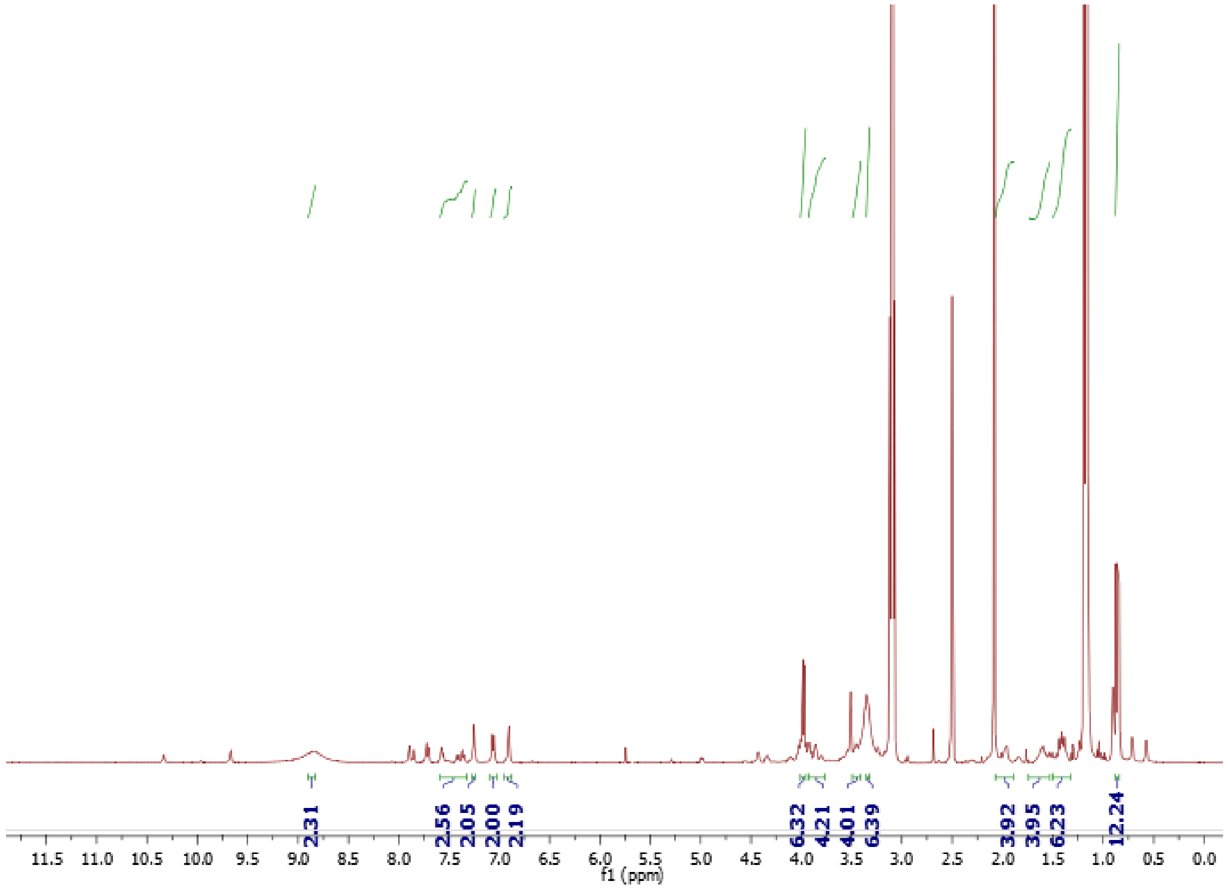

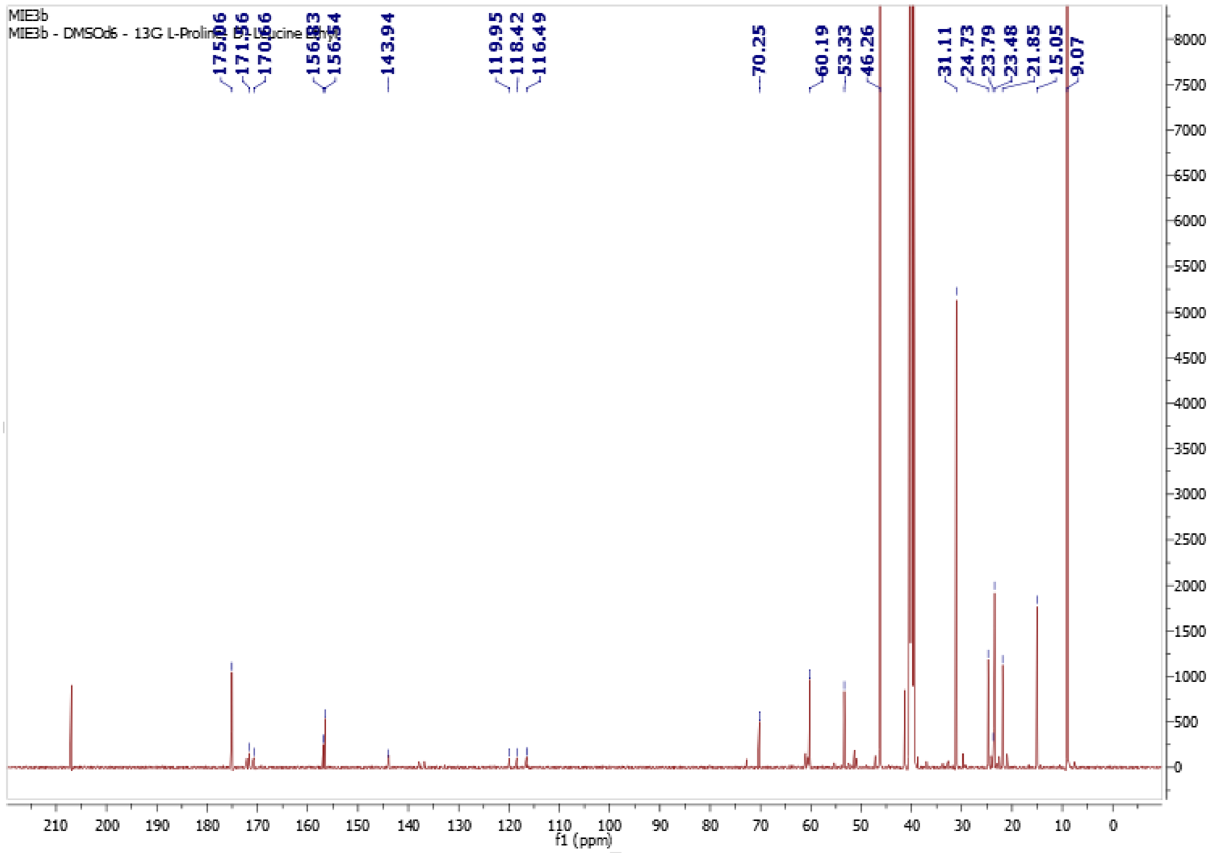
MIE3b
MIE3b - DMSOd6 - 13C L-Proline B-Leucine Ethyl
175.06
171.56
170.66
156.83
156.54
143.94
119.95
118.42
116.49
70.25
60.19
53.33
46.26
31.11
24.73
23.79
23.48
21.85
15.05
9.07
f1 (ppm)

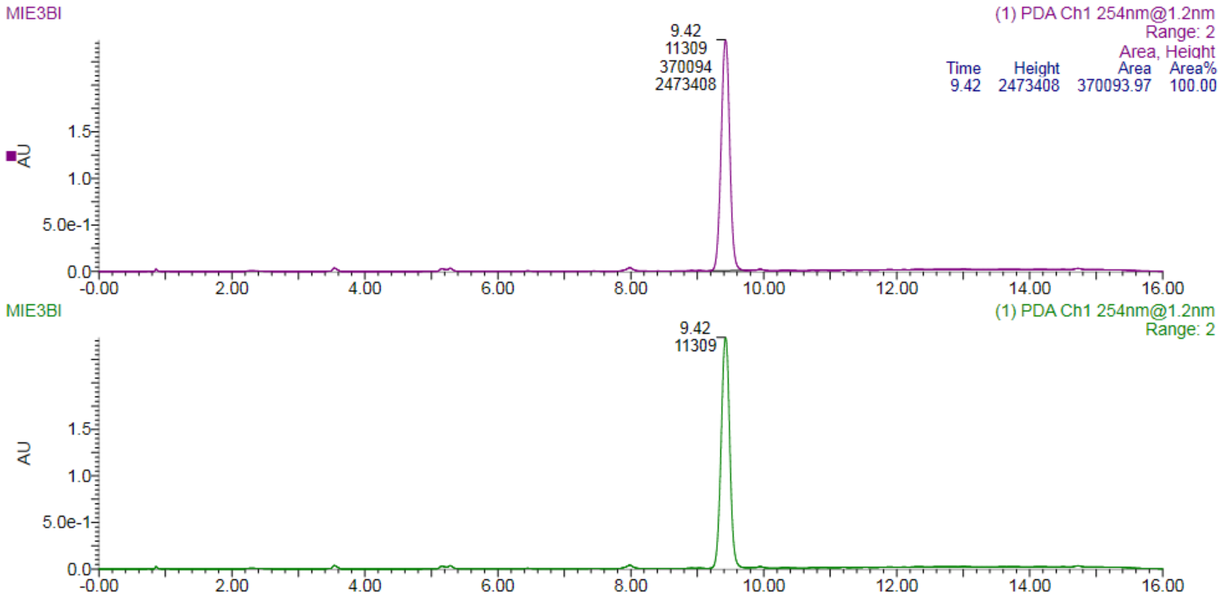
MIE3BI
(1) PDA Ch1 254nm@1.2nm
Range: 2
Area, Height
9.42
11309
370094
2473408
Time Height Area Area%
9.42 2473408 370093.97 100.00
AU
MIE3BI
(1) PDA Ch1 254nm@1.2nm
Range: 2
9.42
11309
AU

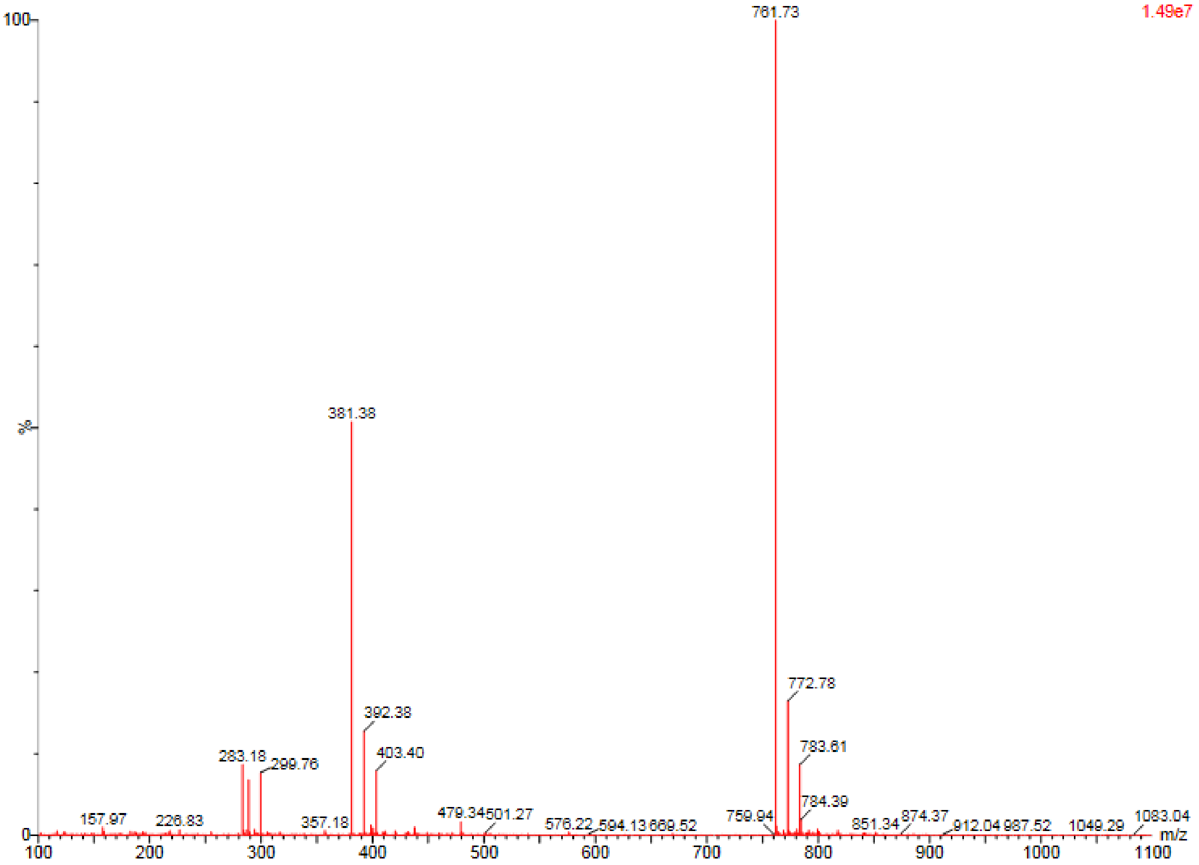

100
%
0
761.73
1.49e7
381.38
772.78
392.38
783.61
283.18
299.76
403.40
784.39
157.97
226.83
357.18
479.34
501.27
576.22
594.13
669.52
759.94
851.34
874.37
912.04
987.52
1049.29
1083.04
m/z
100
200
300
400
500
600
700
800
900
1000
1100

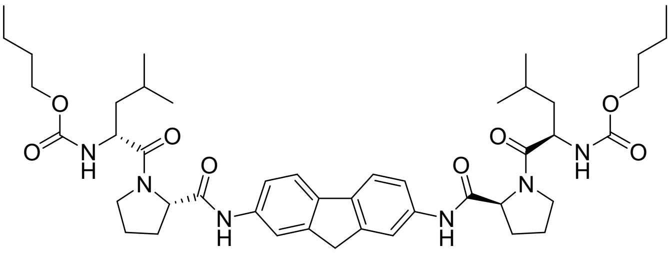

15

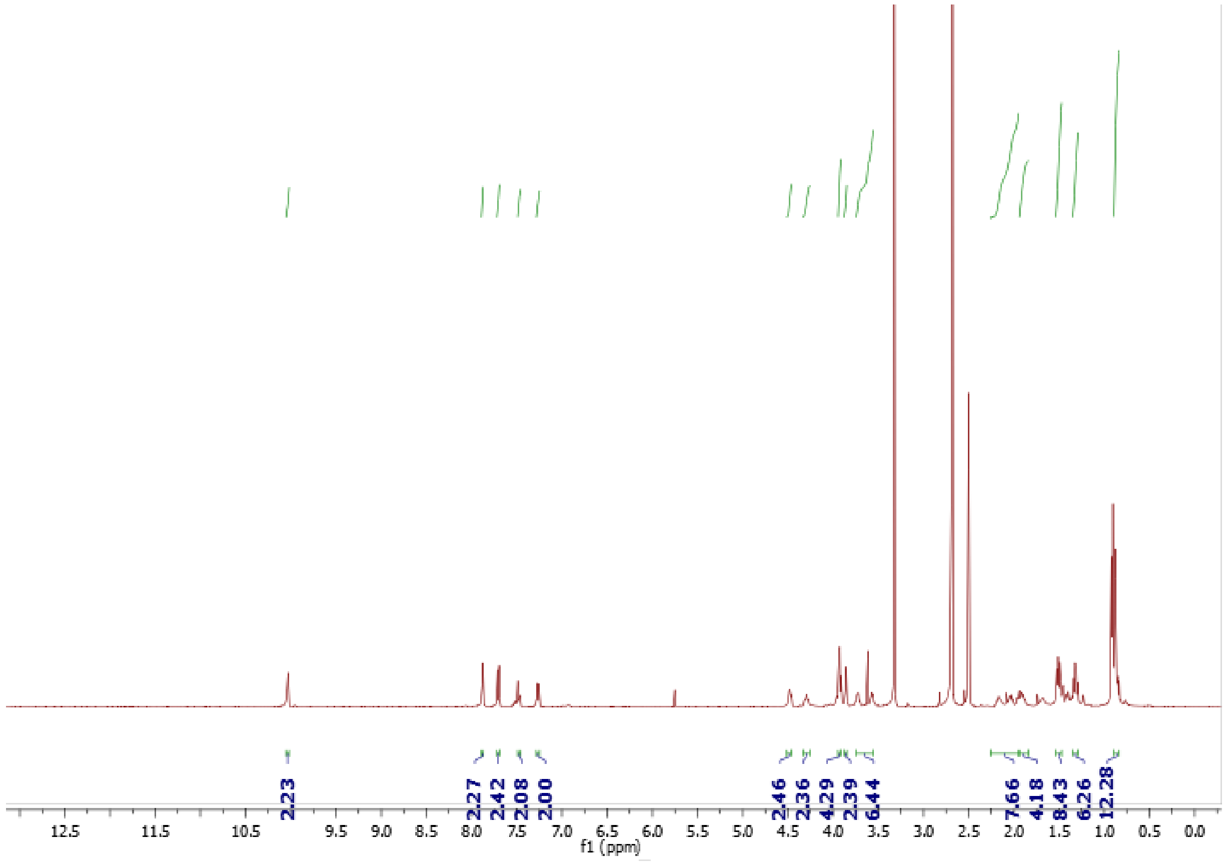
2.23
2.27
2.42
2.08
2.00
2.46
2.36
4.29
2.39
6.44
7.66
4.18
8.43
6.26
12.28
f1 (ppm)

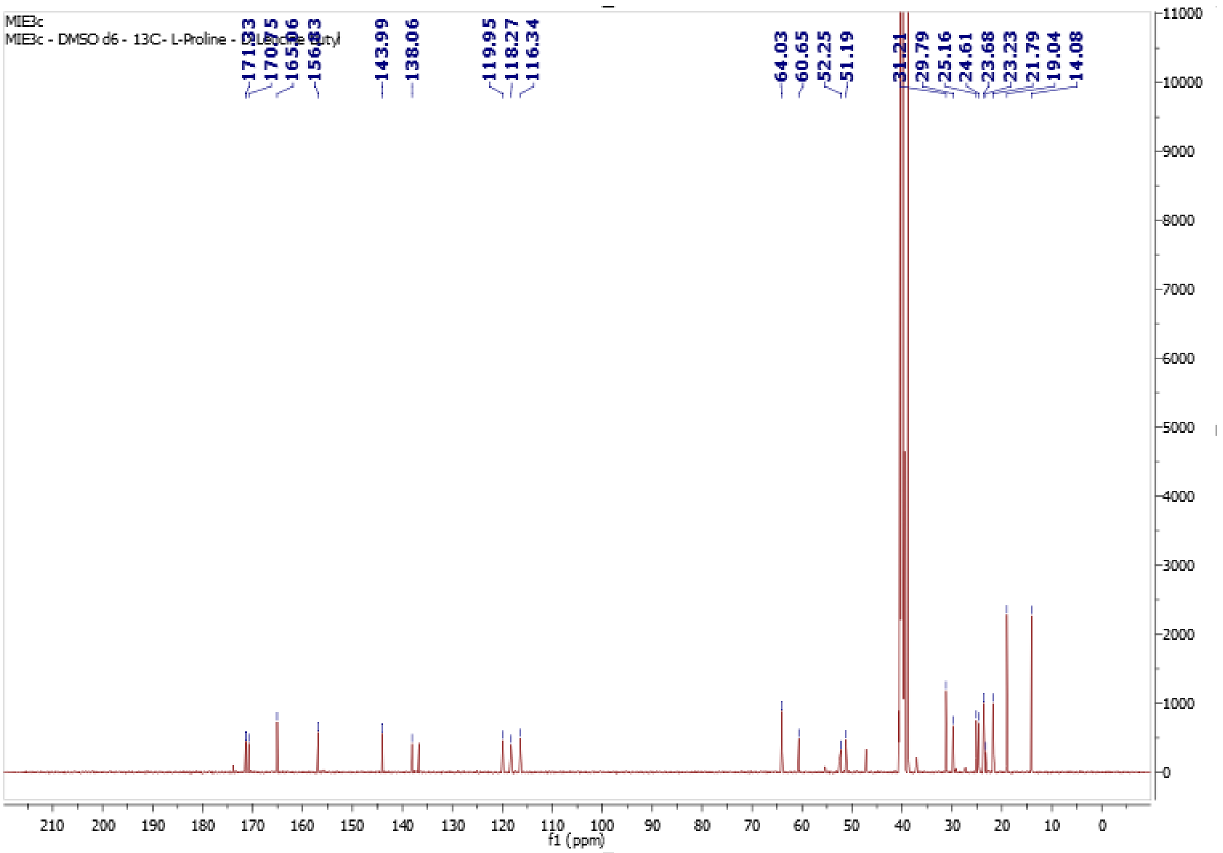
MIE3c
MIE3c - DMSO d6 - 13C- L-Proline - D-Leucine butyl
171.33
170.75
165.36
156.83
143.99
138.06
119.95
118.27
116.34
64.03
60.65
52.25
51.19
31.21
29.79
25.16
24.61
23.68
23.23
21.79
19.04
14.08
f1 (ppm)

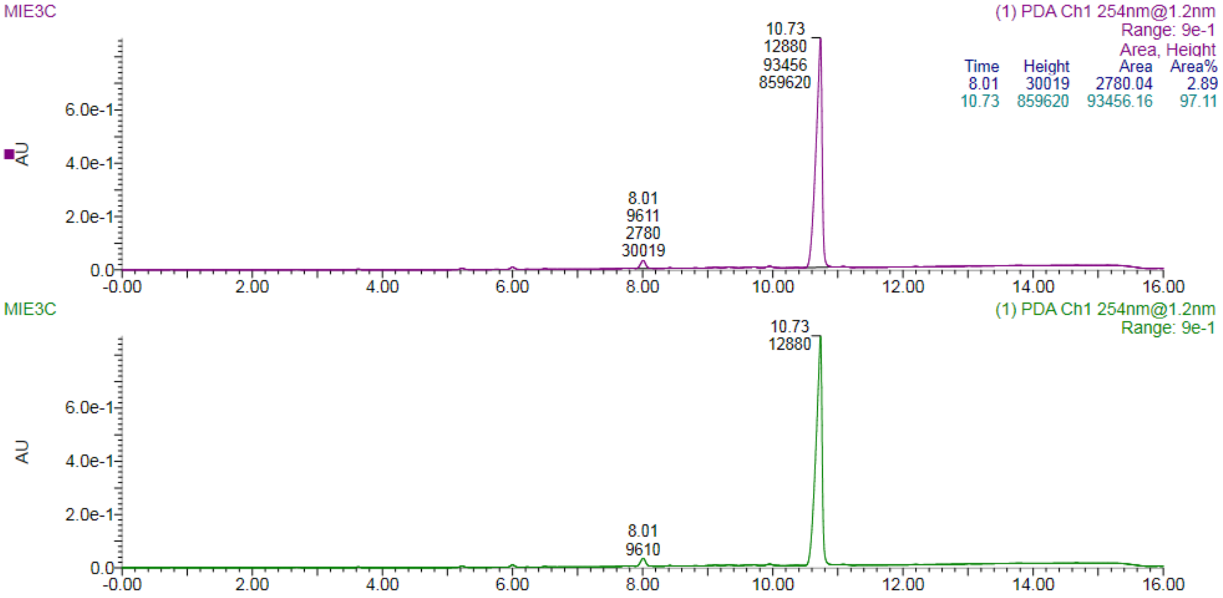
MIE3C
(1) PDA Ch1 254nm@1.2nm
Range: 9e-1
Area, Height
Time Height Area Area%
8.01 30019 2780.04 2.89
10.73 859620 93456.16 97.11
10.73
12880
93456
859620
8.01
9611
2780
30019
AU
MIE3C
(1) PDA Ch1 254nm@1.2nm
Range: 9e-1
10.73
12880
8.01
9610
AU

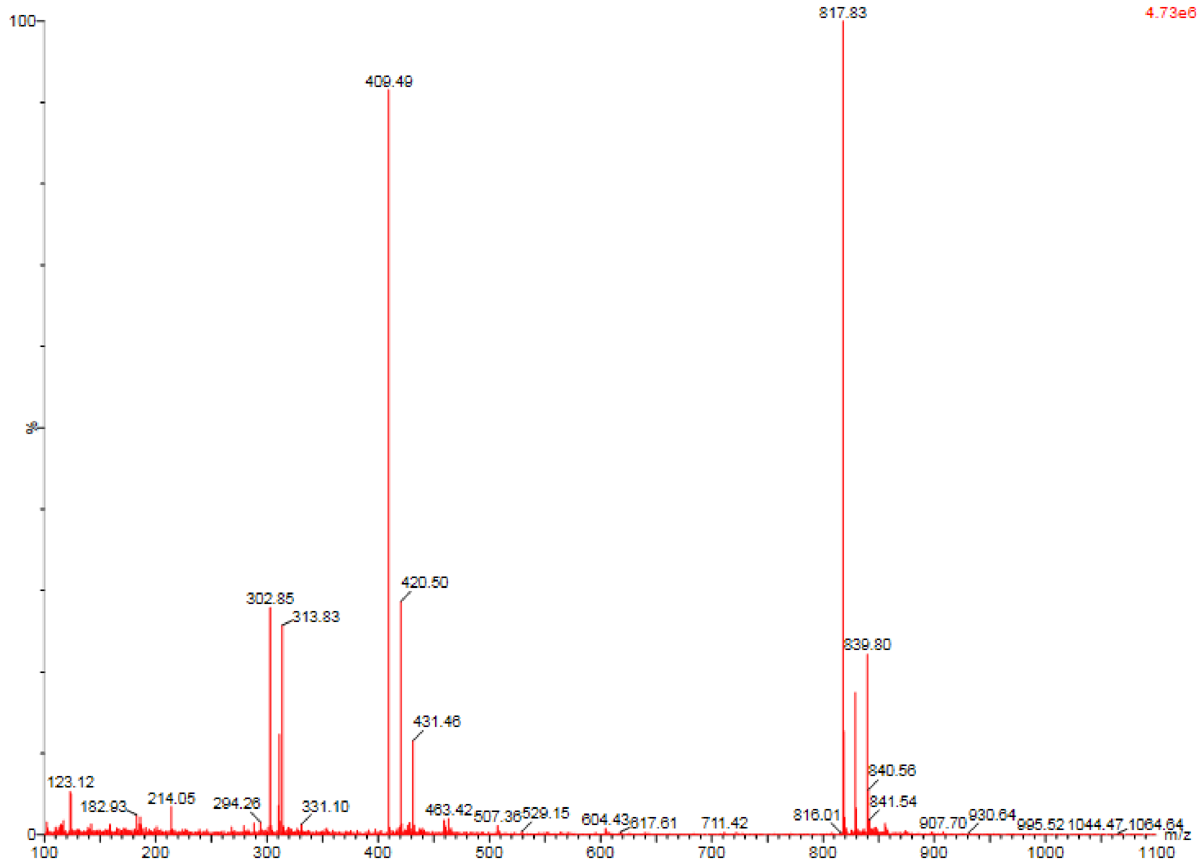
100
%
0
817.83
4.73e6
409.49
302.85
313.83
420.50
431.46
839.80
840.56
841.54
123.12
182.93
214.05
294.26
331.10
463.42
507.36
529.15
604.43
617.61
711.42
816.01
907.70
930.64
995.52
1044.47
1064.64
m/z
100
200
300
400
500
600
700
800
900
1000
1100

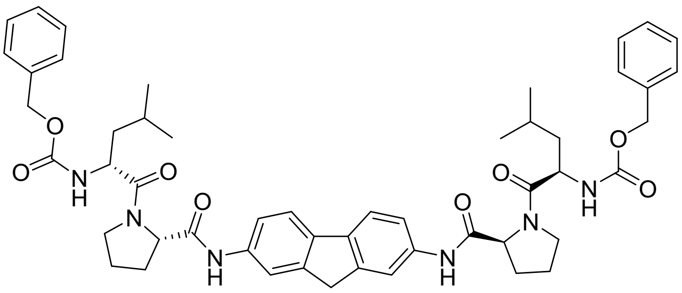

16

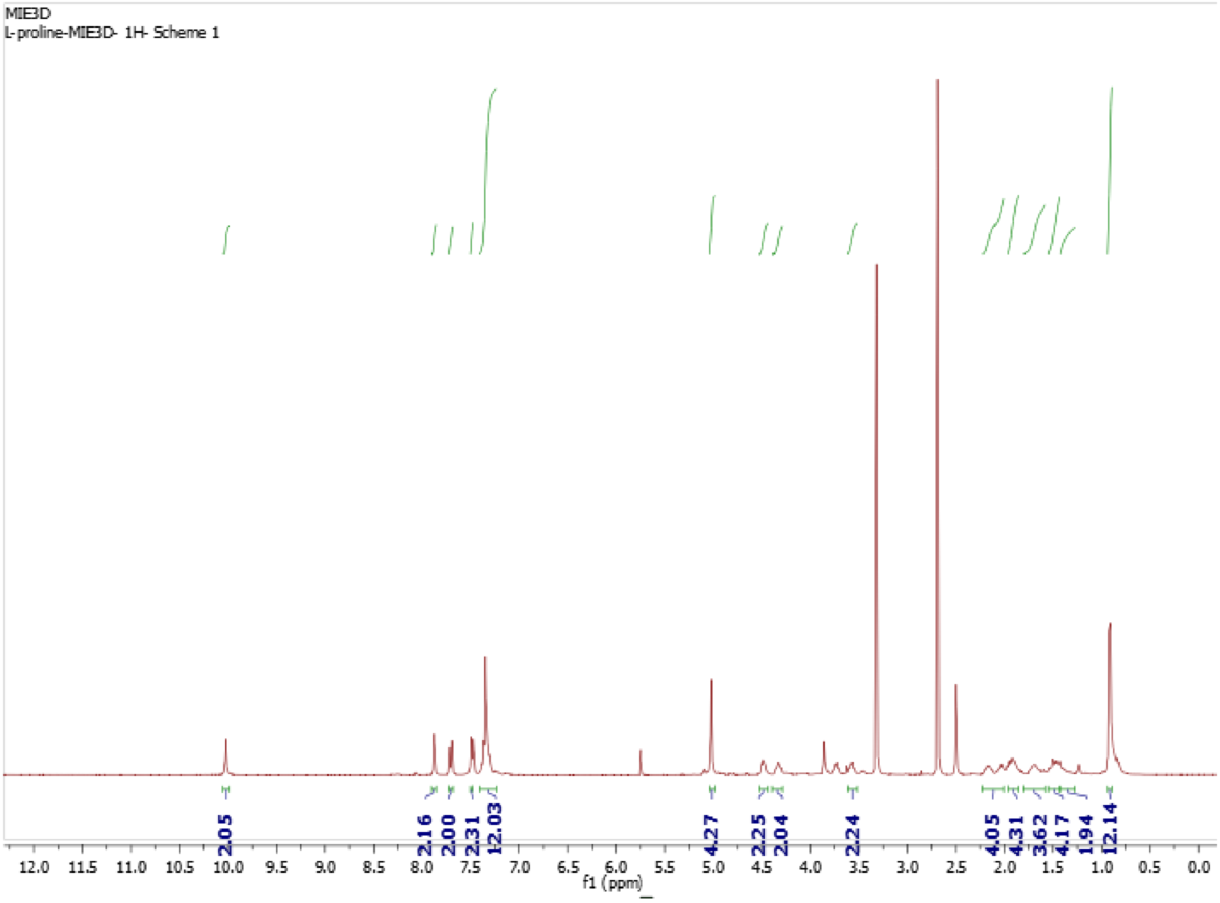
MIE3D
L-proline-MIE3D- 1H- Scheme 1
2.05
2.16
2.00
2.31
12.03
4.27
2.25
2.04
2.24
4.05
4.31
3.62
4.17
1.94
12.14
12.0 11.5 11.0 10.5 10.0 9.5 9.0 8.5 8.0 7.5 7.0 6.5 6.0 5.5 5.0 4.5 4.0 3.5 3.0 2.5 2.0 1.5 1.0 0.5 0.0
f1 (ppm)

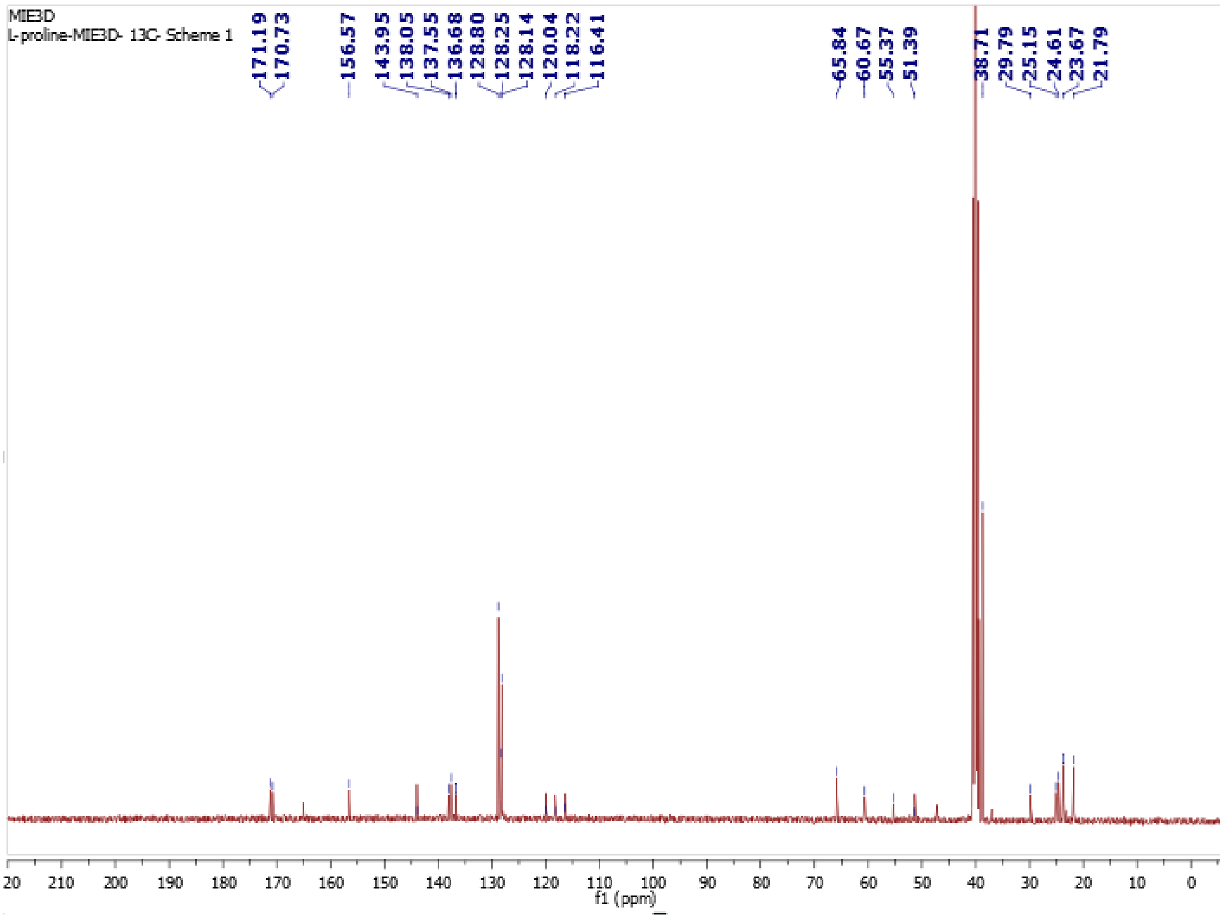
MIE3D
L-proline-MIE3D- 13C- Scheme 1
171.19
170.73
156.57
143.95
138.05
137.55
136.68
128.80
128.25
128.14
120.04
118.22
116.41
65.84
60.67
55.37
51.39
38.71
29.79
25.15
24.61
23.67
21.79
f1 (ppm)

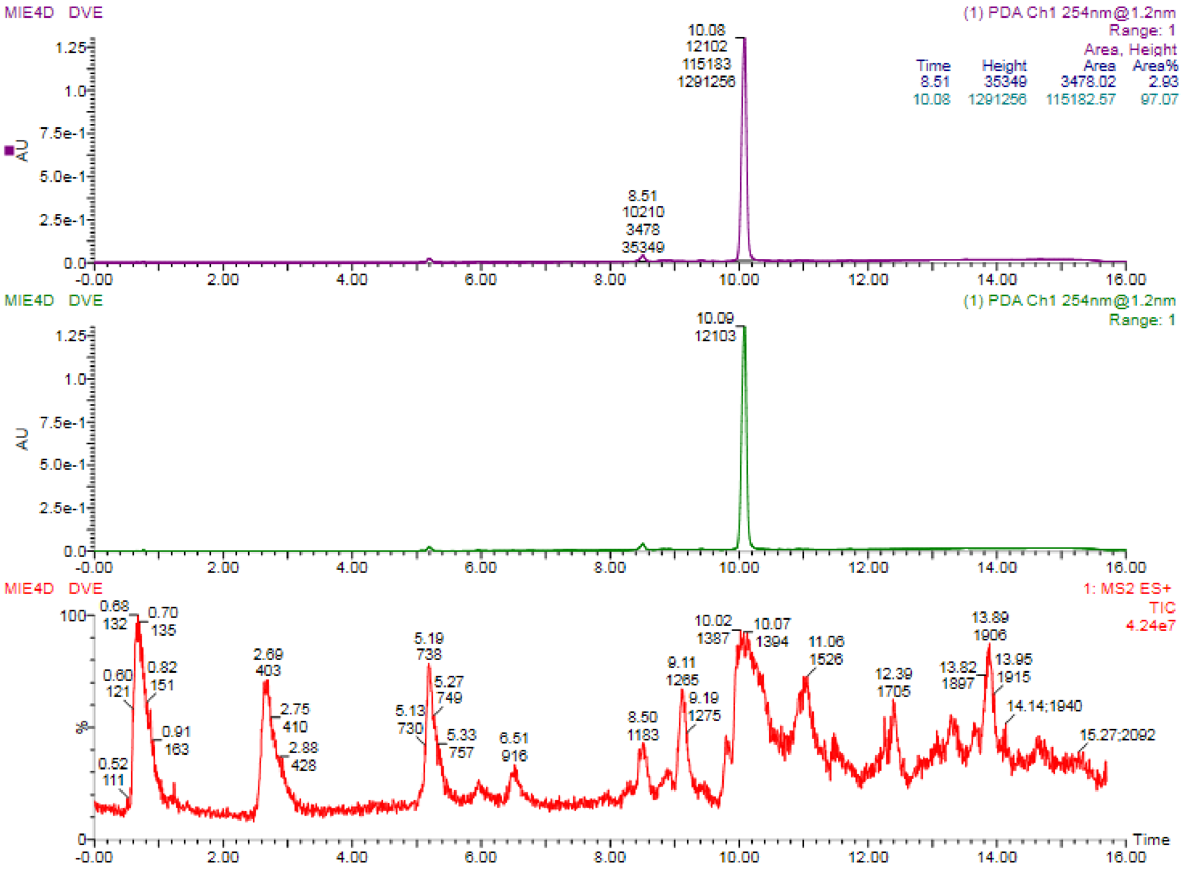

MIE4D DVE
(1) PDA Ch1 254nm@1.2nm
Range: 1
Area, Height
Time Height Area Area%
8.51 35349 3478.02 2.93
10.08 1291256 115182.57 97.07
MIE4D DVE
(1) PDA Ch1 254nm@1.2nm
Range: 1
MIE4D DVE
1: MS2 ES+
TIC
4.24e7
AU
%
Time

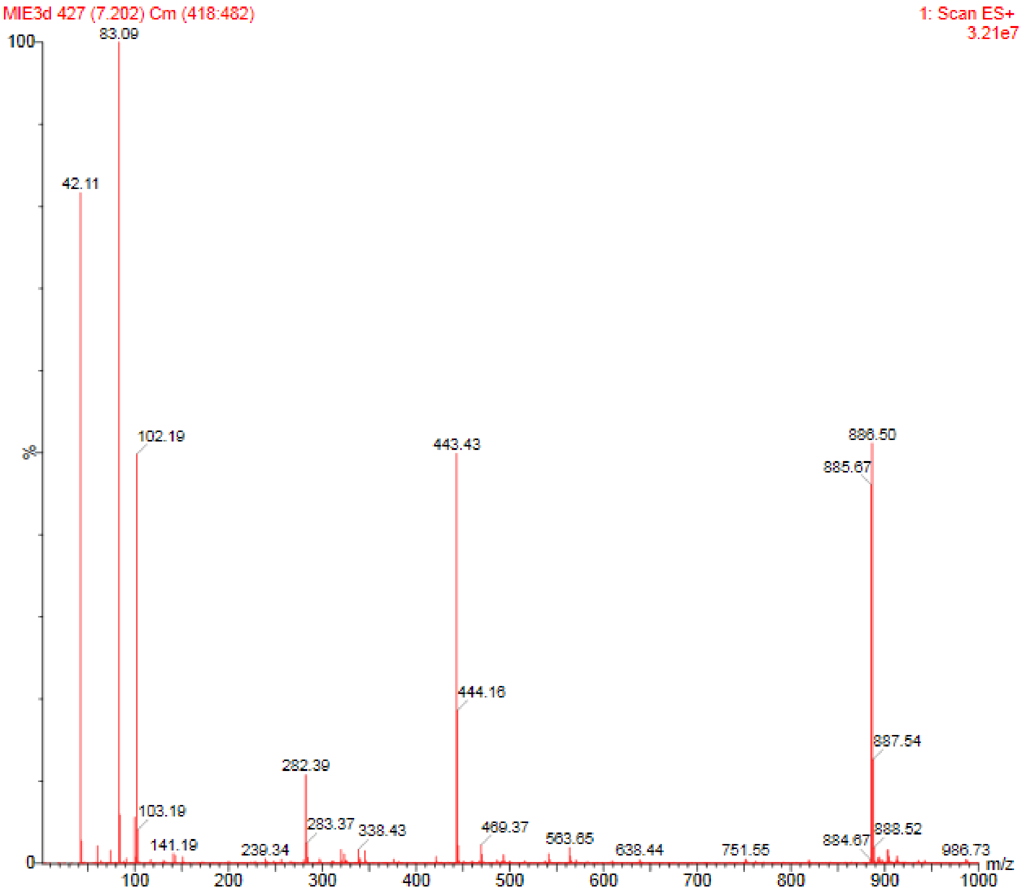
MIE3d 427 (7.202) Cm (418:482)
1: Scan ES+
3.21e7
100
%
0
83.09
42.11
102.19
443.43
886.50
885.67
444.16
887.54
282.39
103.19
283.37
338.43
469.37
888.52
141.19
563.65
884.67
239.34
638.44
751.55
986.73
m/z
100
200
300
400
500
600
700
800
900
1000

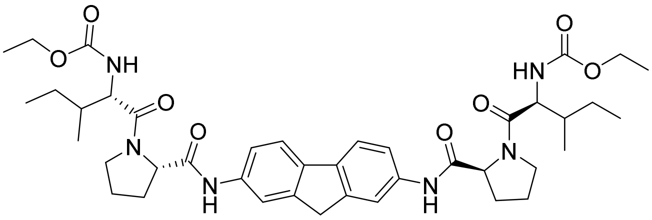
O
NH
O
O
N
O
H
N
HN
O
O
O
N
O
N
H

18

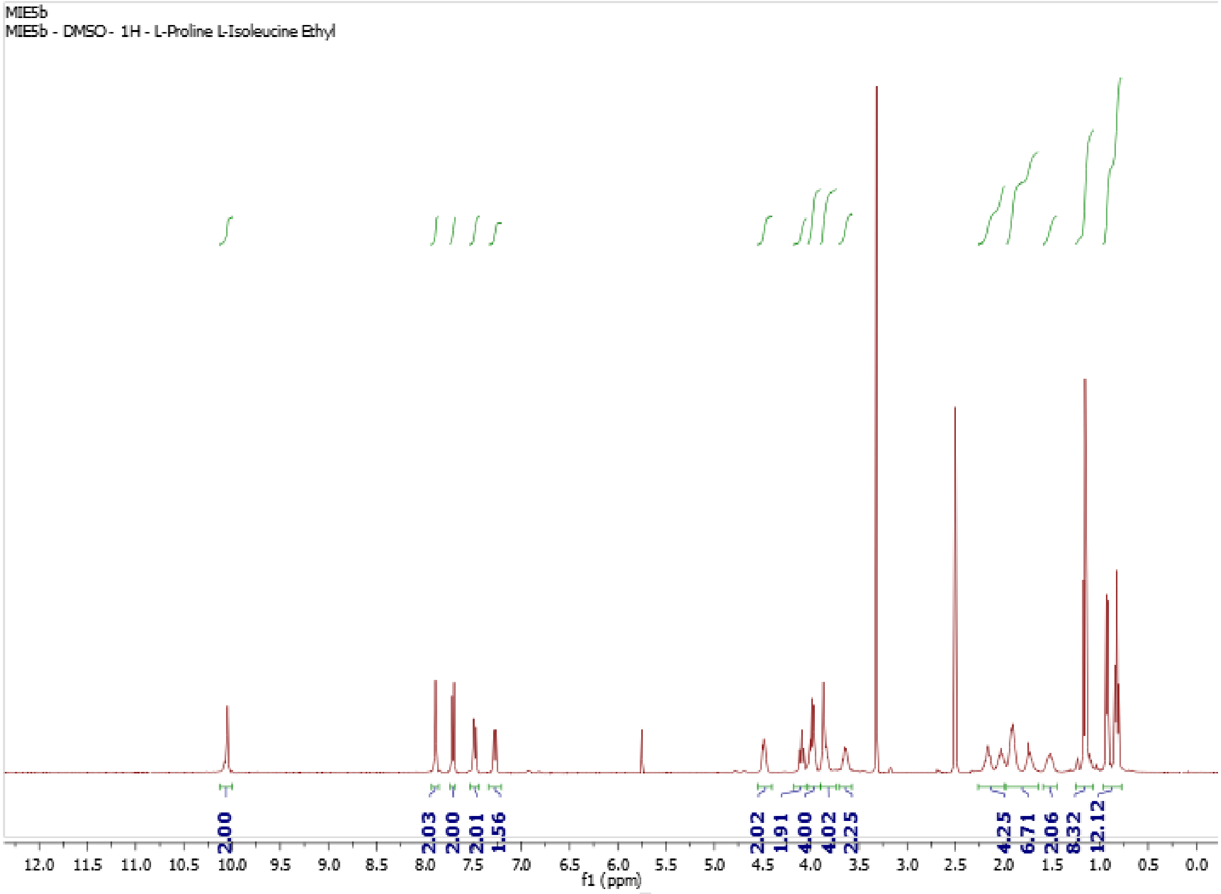
MIE5b
MIE5b - DMSO - 1H - L-Proline L-Isoleucine Ethyl
2.00
2.03
2.00
2.01
1.56
2.02
1.91
4.00
4.02
2.25
4.25
6.71
2.06
8.32
12.12
12.0
11.5
11.0
10.5
10.0
9.5
9.0
8.5
8.0
7.5
7.0
6.5
6.0
5.5
5.0
4.5
4.0
3.5
3.0
2.5
2.0
1.5
1.0
0.5
0.0
f1 (ppm)

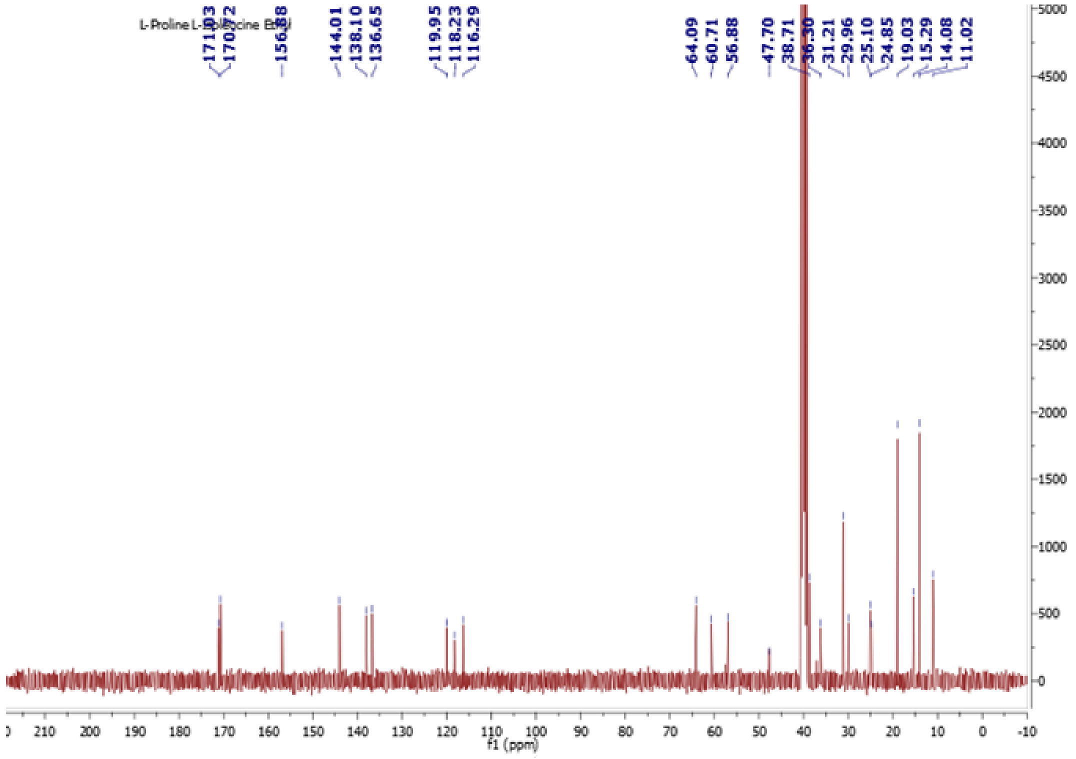
L-Proline L-Isoleucine Ethyl
171.03
170.72
156.88
144.01
138.10
136.65
119.95
118.23
116.29
64.09
60.71
56.88
47.70
38.71
36.30
31.21
29.96
25.10
24.85
19.03
15.29
14.08
11.02
f1 (ppm)

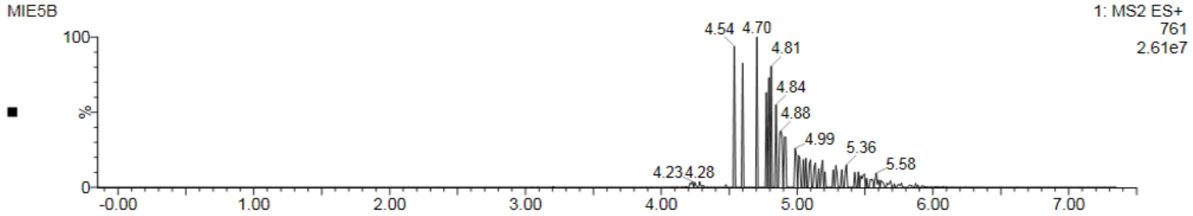
MIE5B
1: MS2 ES+
761
2.61e7
%
4.54 4.70
4.81
4.84
4.88
4.99
5.36
5.58
4.23 4.28

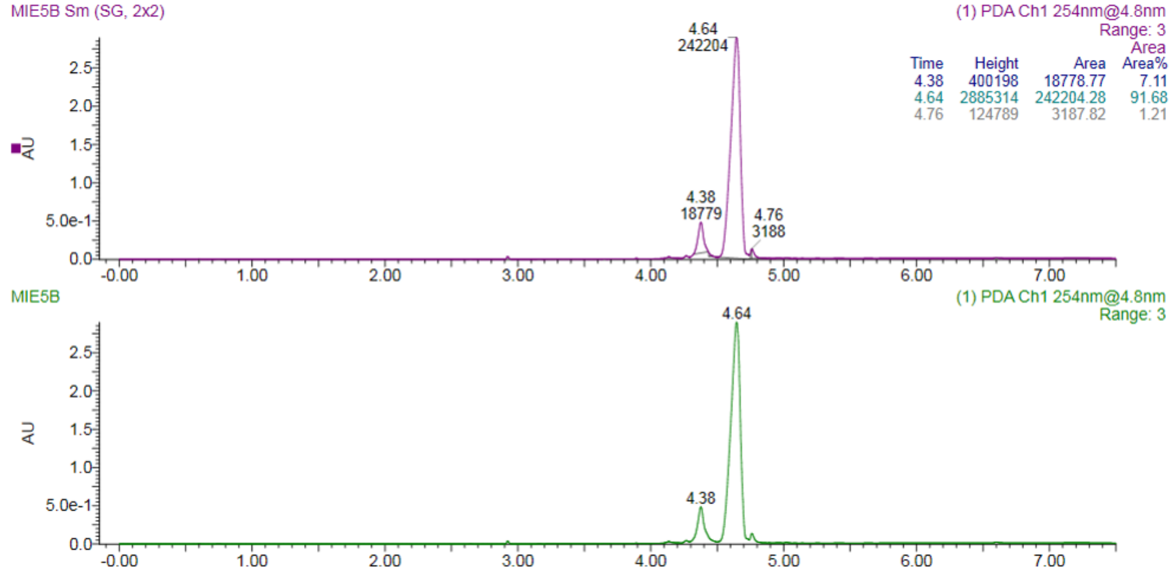
MIE5B Sm (SG, 2x2)
(1) PDA Ch1 254nm@4.8nm
Range: 3
AU
4.64
242204
4.38
18779
4.76
3188
Time Height Area Area%
4.38 400198 18778.77 7.11
4.64 2885314 242204.28 91.68
4.76 124789 3187.82 1.21
MIE5B
(1) PDA Ch1 254nm@4.8nm
Range: 3
4.64
4.38

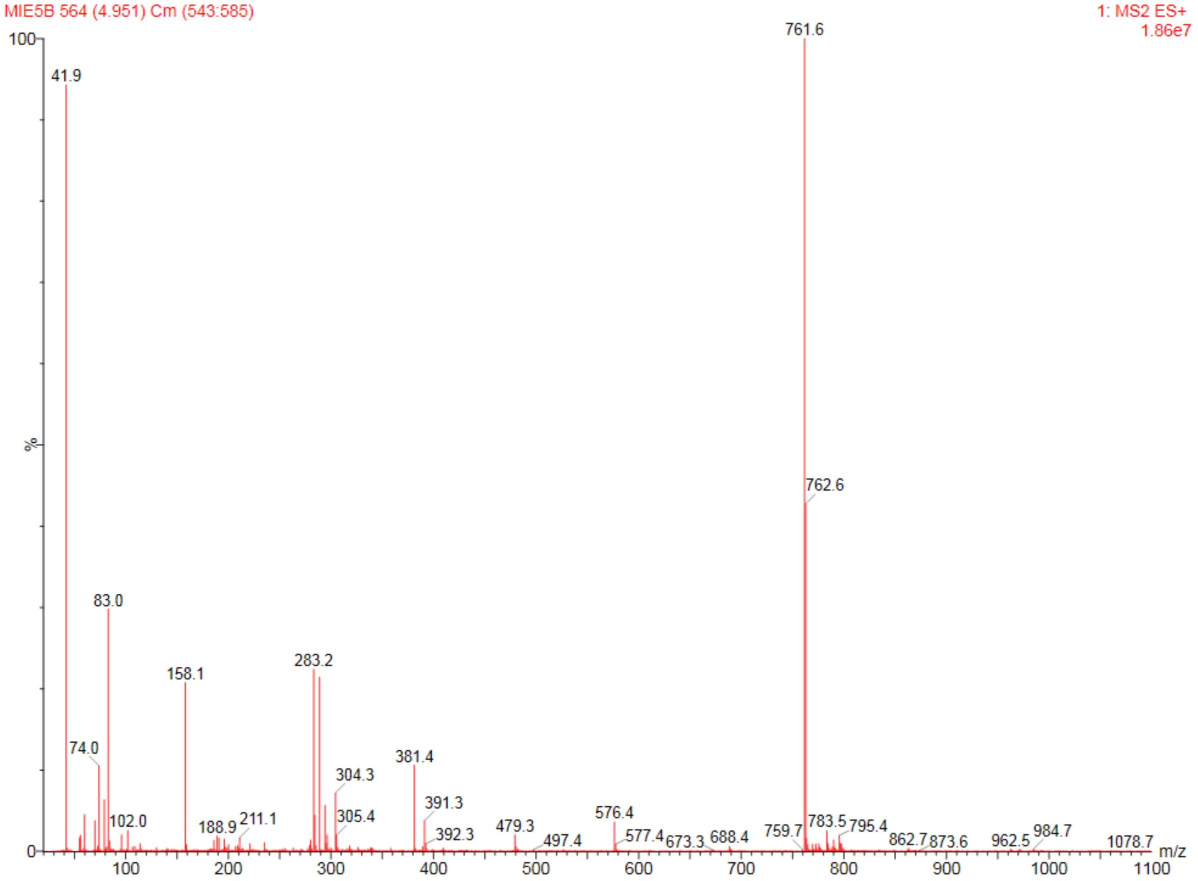
MIE5B 564 (4.951) Cm (543:585)
1: MS2 ES+
1.86e7
761.6
41.9
762.6
83.0
158.1
283.2
74.0
381.4
304.3
391.3
102.0
188.9
211.1
305.4
392.3
479.3
497.4
576.4
577.4
673.3
688.4
759.7
783.5
795.4
862.7
873.6
962.5
984.7
1078.7
m/z
%
100
0
100
200
300
400
500
600
700
800
900
1000
1100

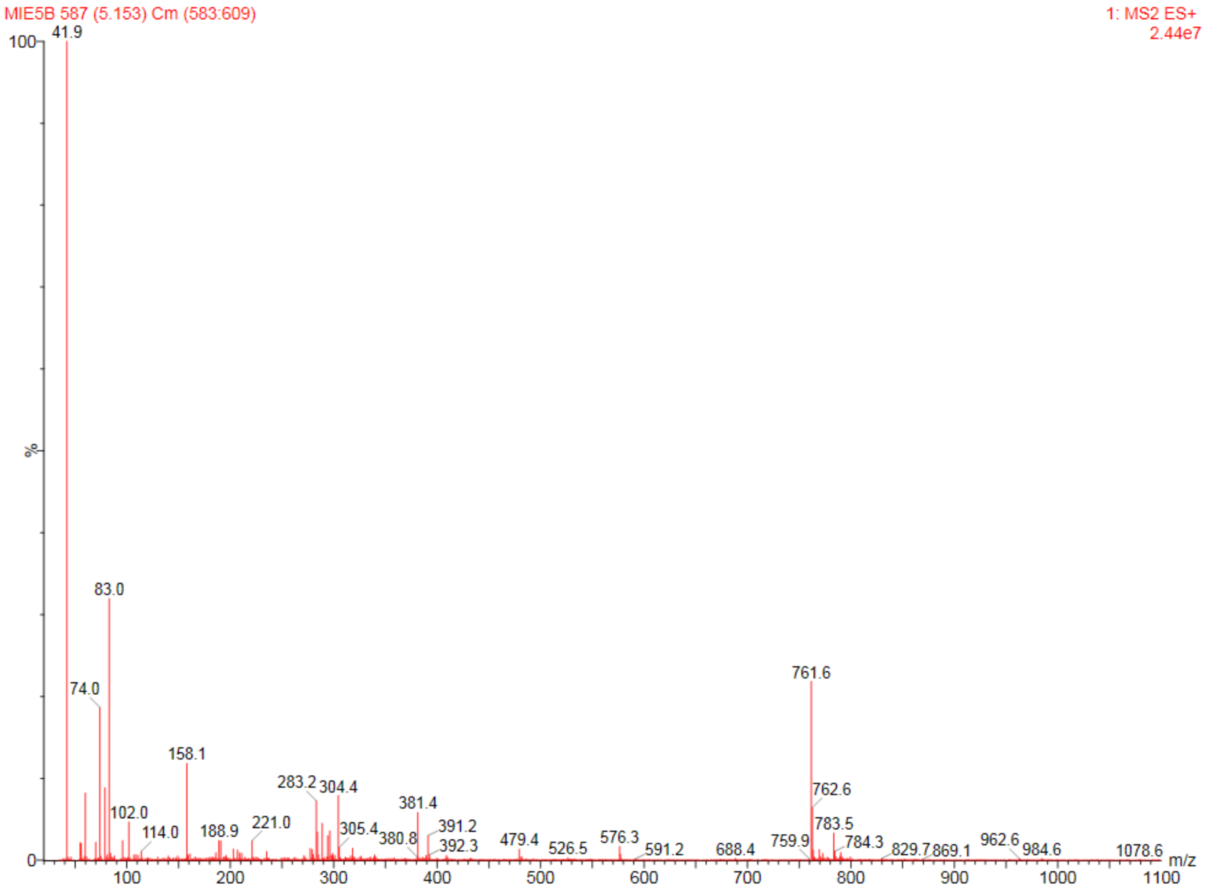
MIE5B 587 (5.153) Cm (583:609)
1: MS2 ES+
2.44e7
100
%
0
41.9
83.0
74.0
158.1
102.0
114.0
188.9
221.0
283.2
304.4
305.4
380.8
381.4
391.2
392.3
479.4
526.5
576.3
591.2
688.4
759.9
761.6
762.6
783.5
784.3
829.7
869.1
962.6
984.6
1078.6
m/z
100
200
300
400
500
600
700
800
900
1000
1100

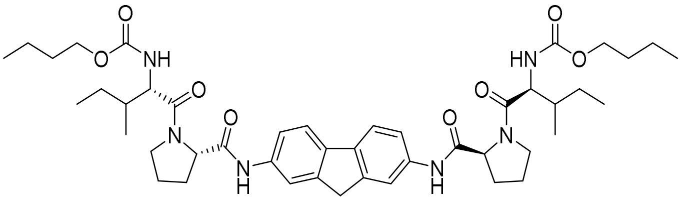

19

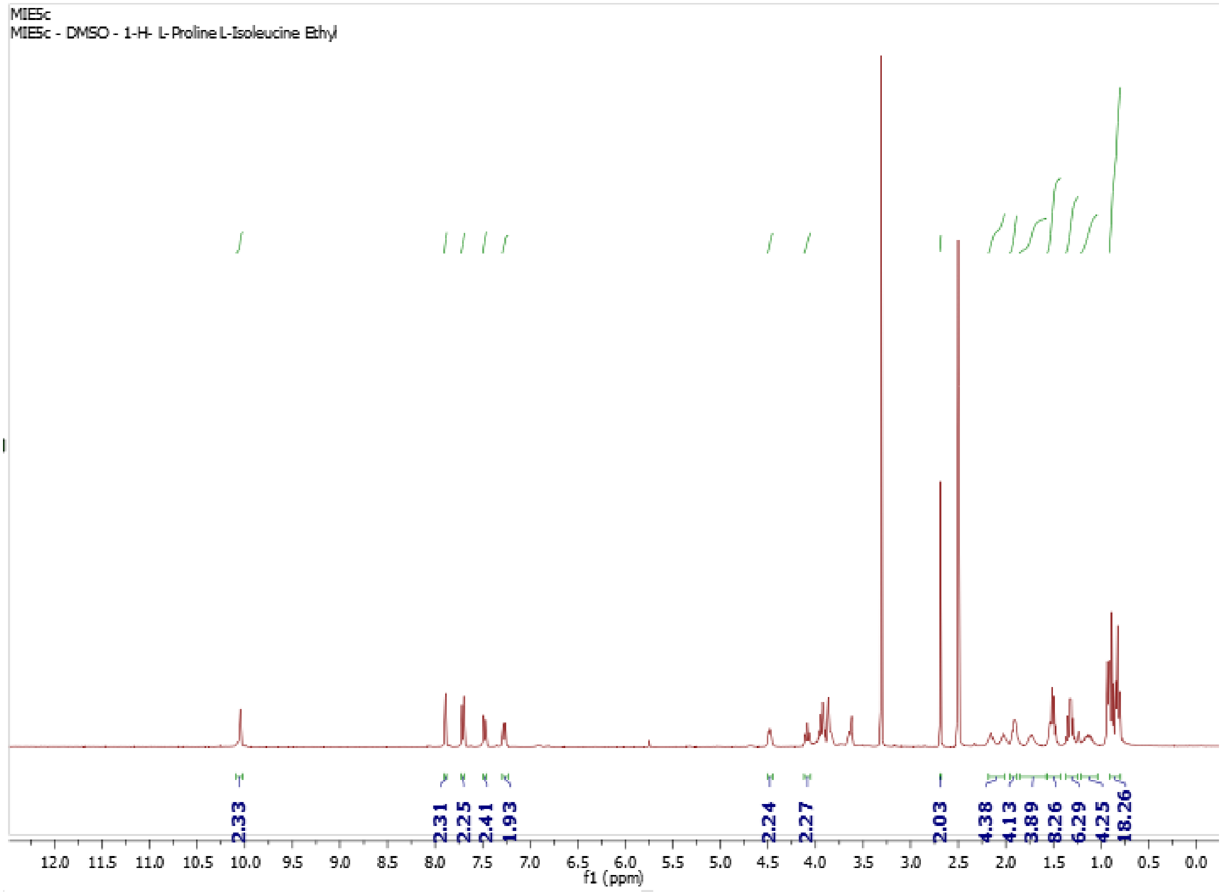

MIE5c
MIE5c - DMSO - 1-H- L-Proline L-Isoleucine Ethyl
2.33
2.31
2.25
2.41
1.93
2.24
2.27
2.03
4.38
4.13
3.89
8.26
6.29
4.25
18.26
12.0 11.5 11.0 10.5 10.0 9.5 9.0 8.5 8.0 7.5 7.0 6.5 6.0 5.5 5.0 4.5 4.0 3.5 3.0 2.5 2.0 1.5 1.0 0.5 0.0
f1 (ppm)

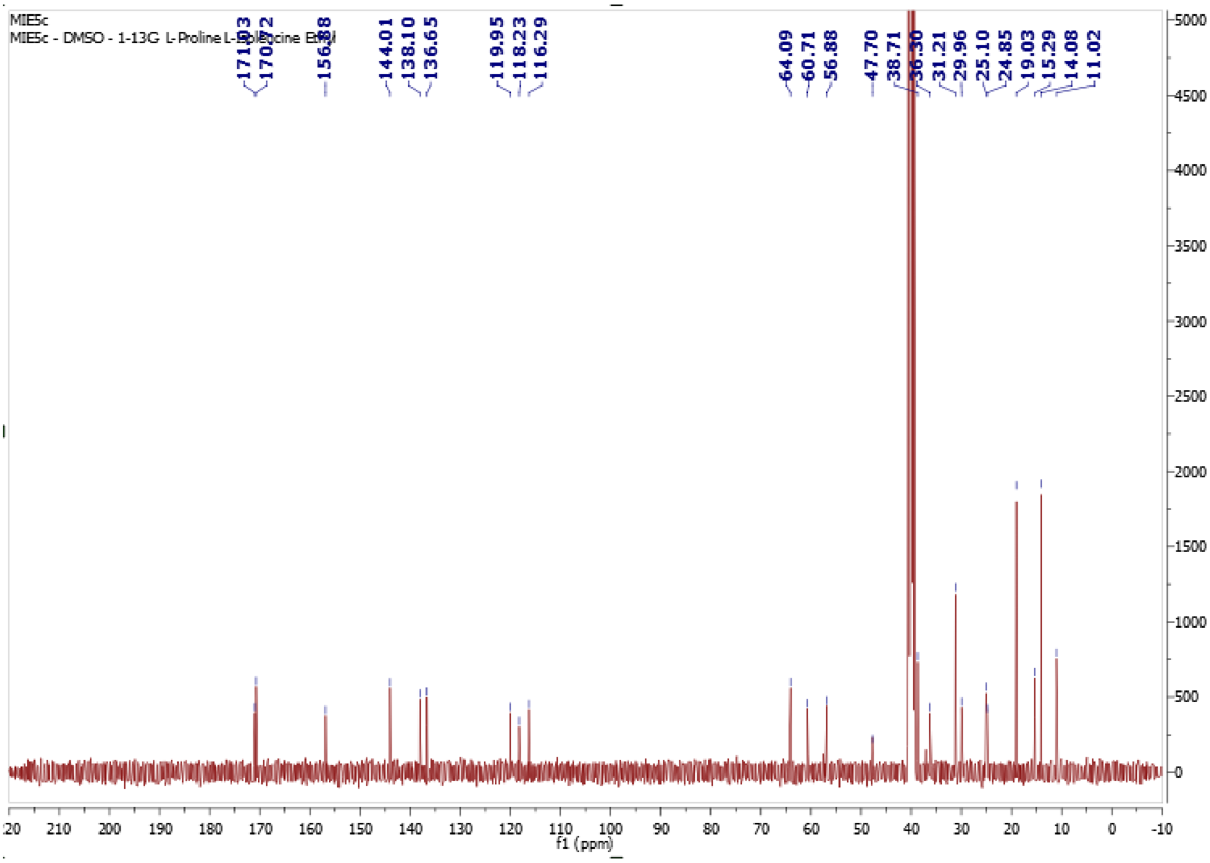
MIE5c
MIE5c - DMSO - 1-13C L-Proline L-Isoleucine Ethyl
171.03
170.22
156.88
144.01
138.10
136.65
119.95
118.23
116.29
64.09
60.71
56.88
47.70
38.71
36.50
31.21
29.96
25.10
24.85
19.03
15.29
14.08
11.02
f1 (ppm)

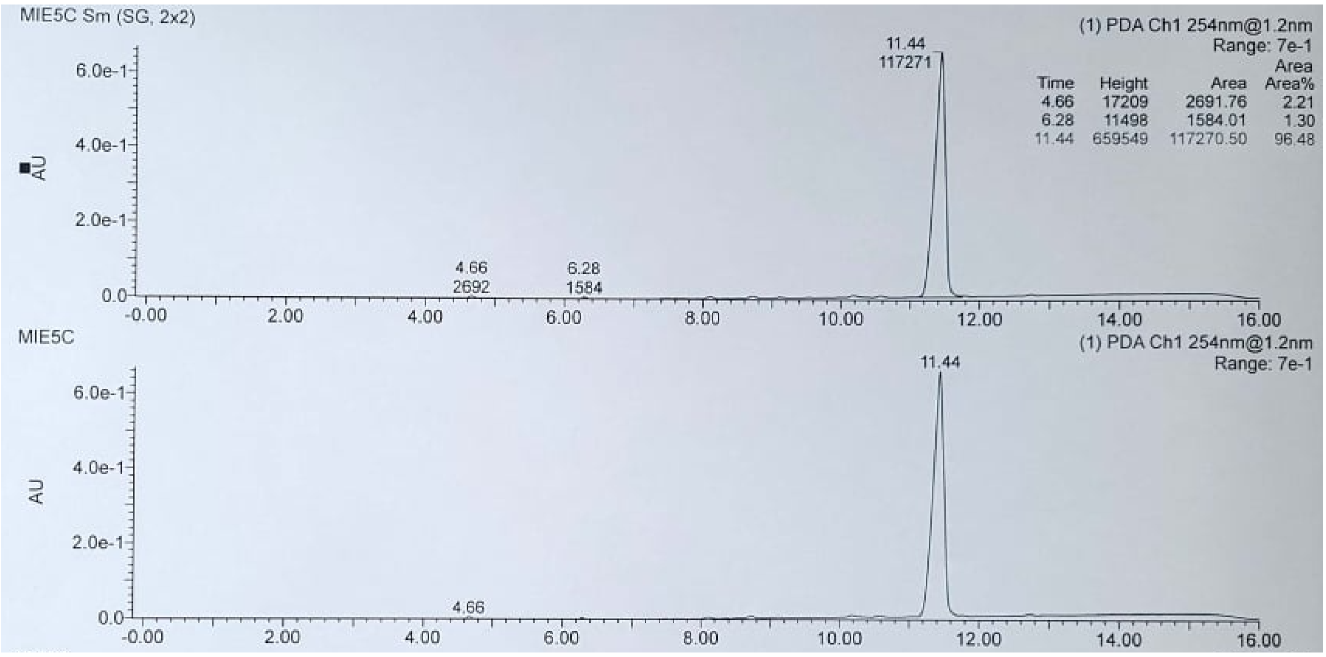
MIE5C Sm (SG, 2x2)
(1) PDA Ch1 254nm@1.2nm
Range: 7e-1
11.44
117271
Time Height Area Area%
4.66 17209 2691.76 2.21
6.28 11498 1584.01 1.30
11.44 659549 117270.50 96.48
AU
4.66
2692
6.28
1584
MIE5C
(1) PDA Ch1 254nm@1.2nm
Range: 7e-1
11.44
AU
4.66

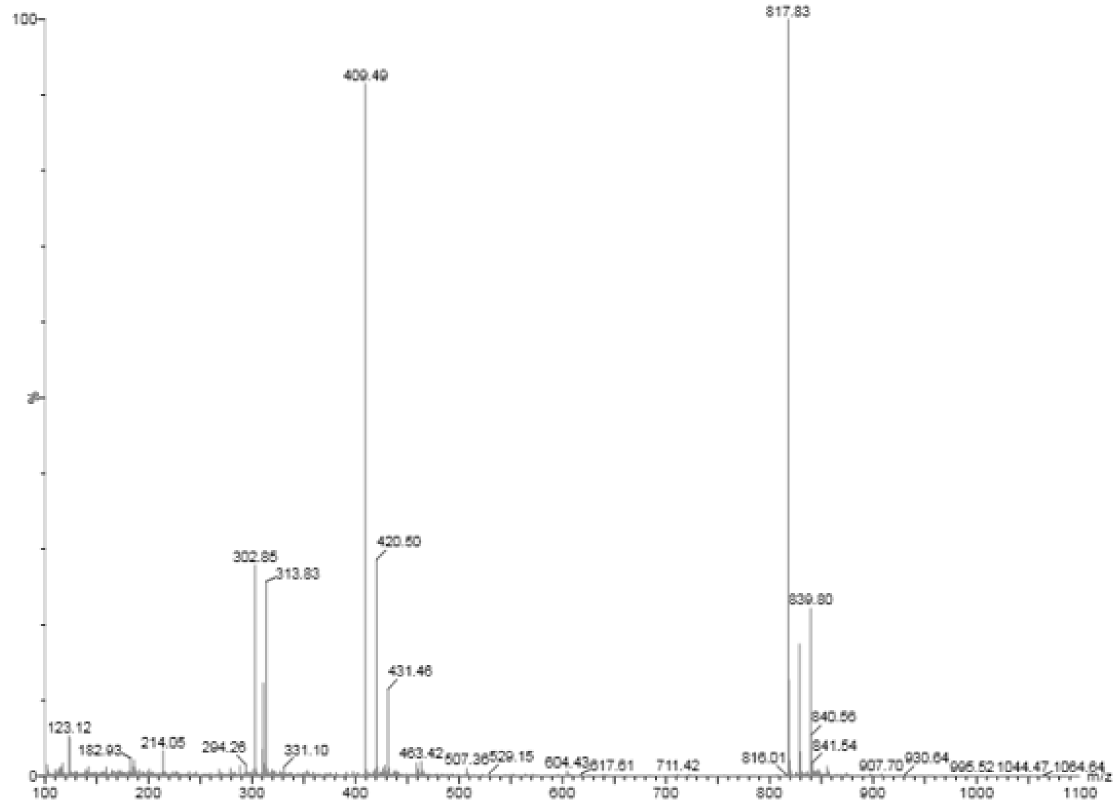

100
%
0
817.83
409.49
420.50
302.85
313.83
839.80
431.46
840.56
123.12
841.54
182.93
214.05
294.26
331.10
463.42
507.36
529.15
604.43
617.61
711.42
816.01
907.70
930.64
995.52
1044.47
1064.64
m/z
100
200
300
400
500
600
700
800
900
1000
1100

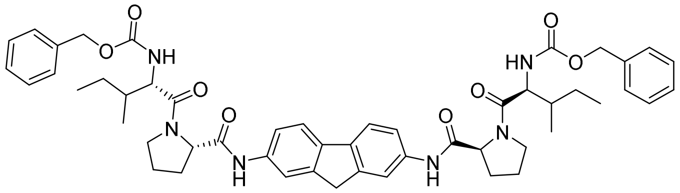

O
NH
O
O
N
N
H
HN
O

20

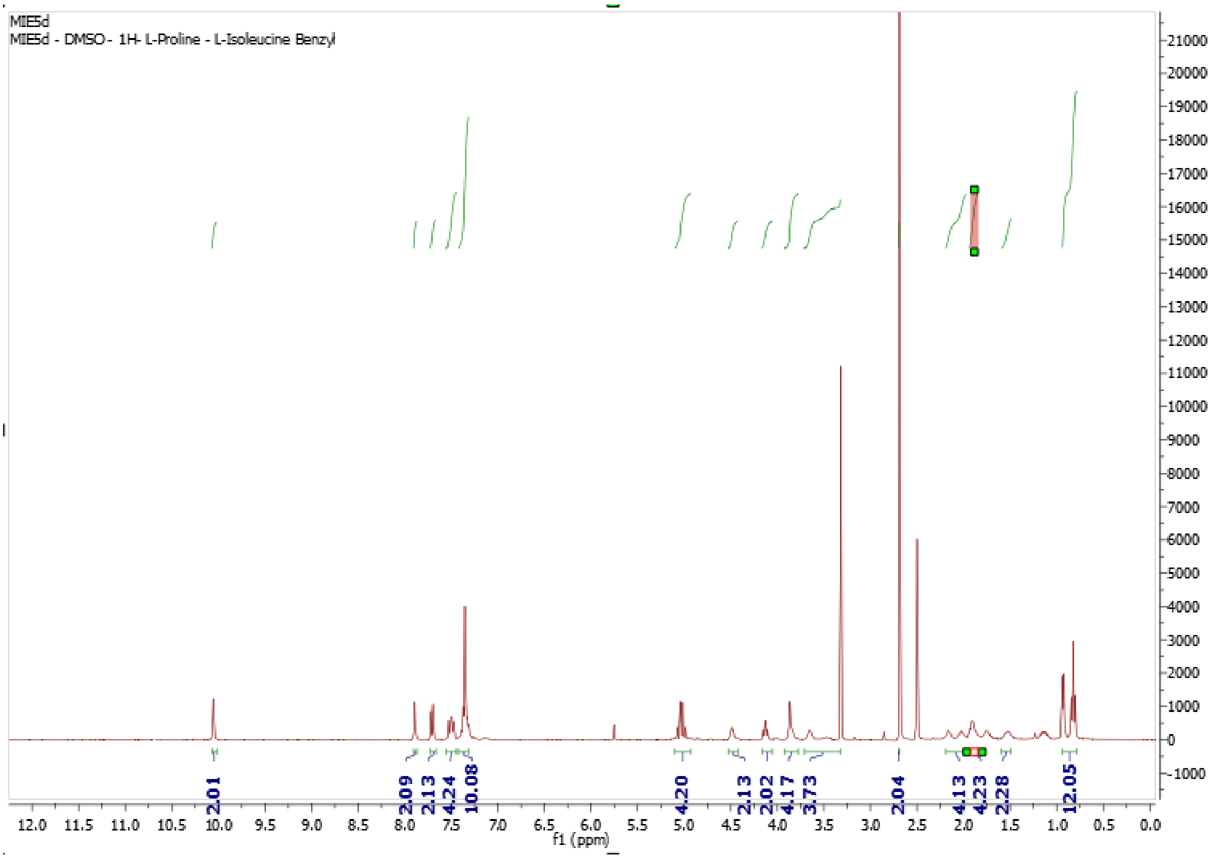

MIE5d
MIE5d - DMSO - 1H- L-Proline - L-Isoleucine Benzyl
2.01
2.09
2.13
4.24
10.08
4.20
2.13
2.02
4.17
3.73
2.04
4.13
4.23
2.28
12.05
f1 (ppm)

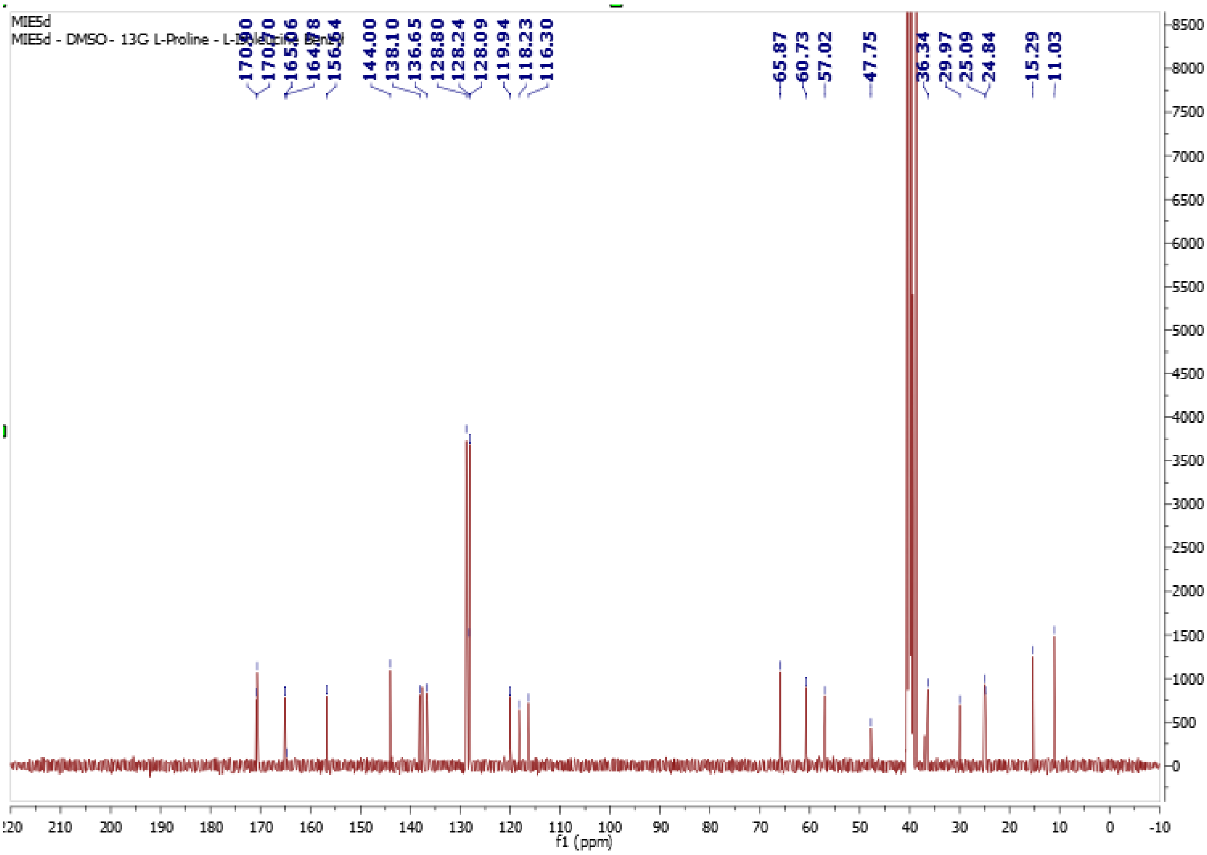
MIE5d
MIE5d - DMSO - 13C L-Proline - L-Isoleucine Benzyl
170.90
170.70
165.06
164.78
156.54
144.00
138.10
136.65
128.80
128.24
128.09
119.94
118.23
116.30
65.87
60.73
57.02
47.75
36.34
29.97
25.09
24.84
15.29
11.03
f1 (ppm)

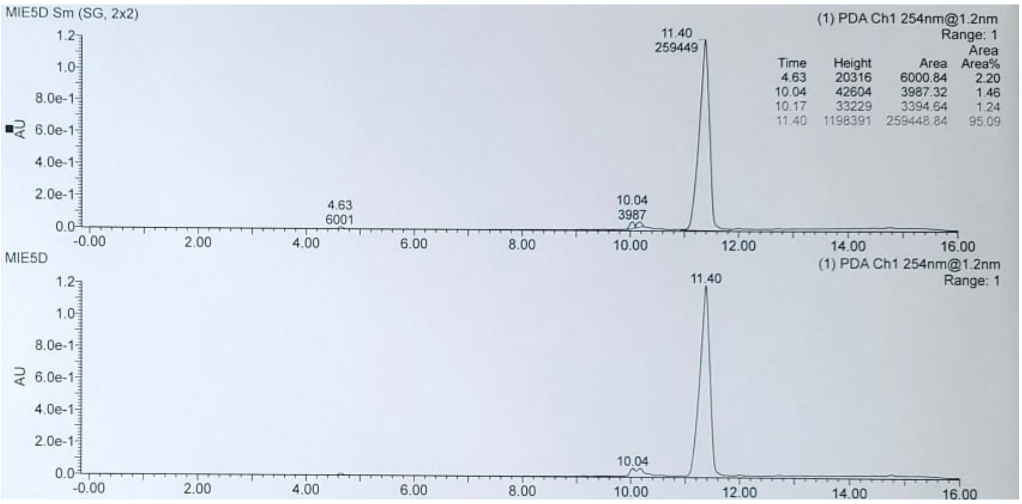
MIE5D Sm (SG, 2x2)
(1) PDA Ch1 254nm@1.2nm
Range: 1
Time Height Area Area%
4.63 20316 6000.84 2.20
10.04 42604 3987.32 1.46
10.17 33229 3394.64 1.24
11.40 1198391 259448.84 95.09
11.40
259449
4.63
6001
10.04
3987
AU
MIE5D
(1) PDA Ch1 254nm@1.2nm
Range: 1
11.40
10.04

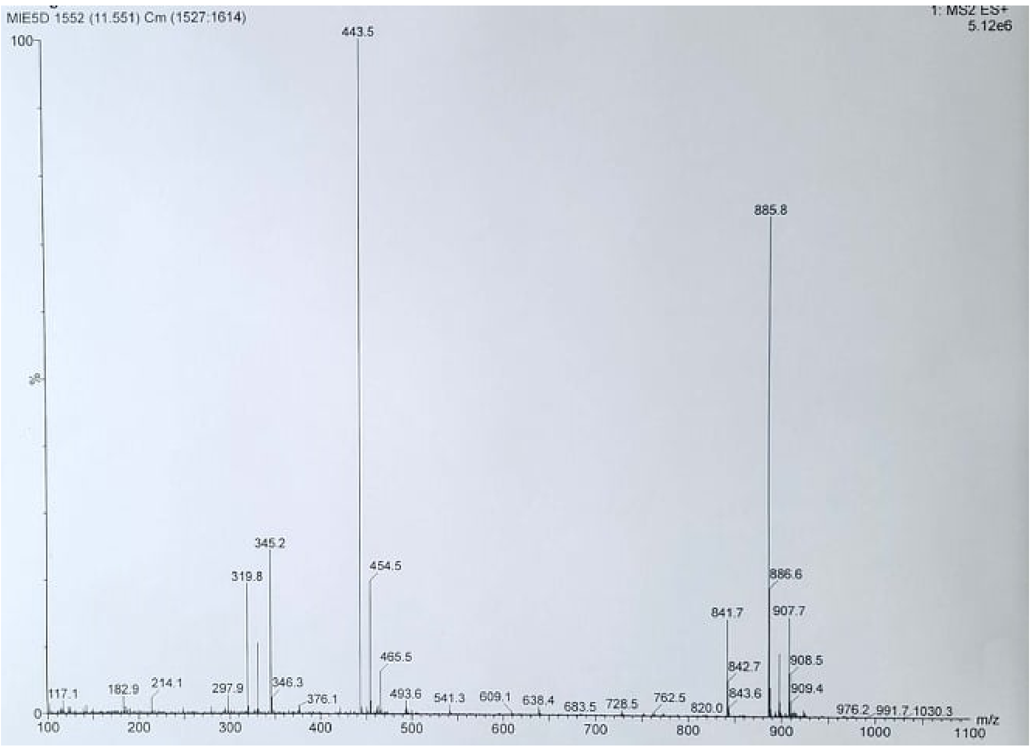
MIE5D 1552 (11.551) Cm (1527:1614)
1: MS2 ES+
5.12e6
100
%
0
443.5
885.8
345.2
454.5
319.8
886.6
841.7
907.7
465.5
908.5
842.7
117.1
182.9
214.1
297.9
346.3
376.1
493.6
541.3
609.1
638.4
683.5
728.5
762.5
820.0
843.6
909.4
976.2
991.7
1030.3
100
200
300
400
500
600
700
800
900
1000
1100
m/z

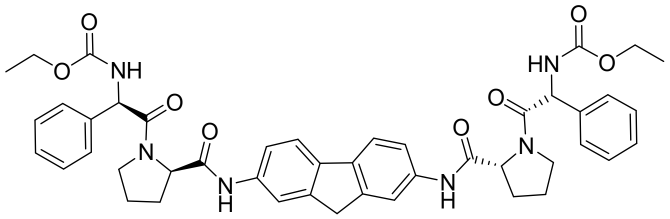

26

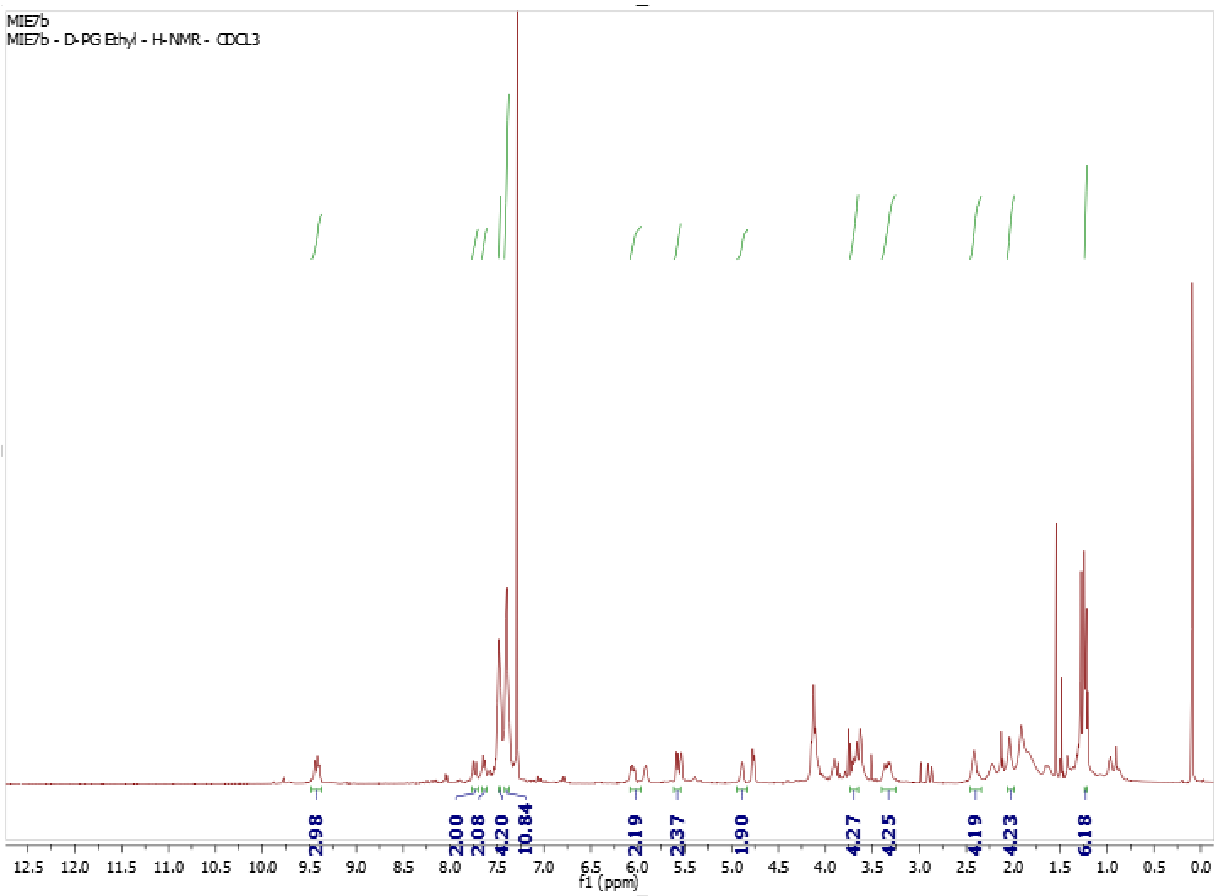
MIE7b
MIE7b - D-PG Ethyl - H-NMR - CDCL3
2.98
2.00
2.08
4.20
10.84
2.19
2.37
1.90
4.27
4.25
4.19
4.23
6.18
12.5
12.0
11.5
11.0
10.5
10.0
9.5
9.0
8.5
8.0
7.5
7.0
6.5
6.0
5.5
5.0
4.5
4.0
3.5
3.0
2.5
2.0
1.5
1.0
0.5
0.0
f1 (ppm)

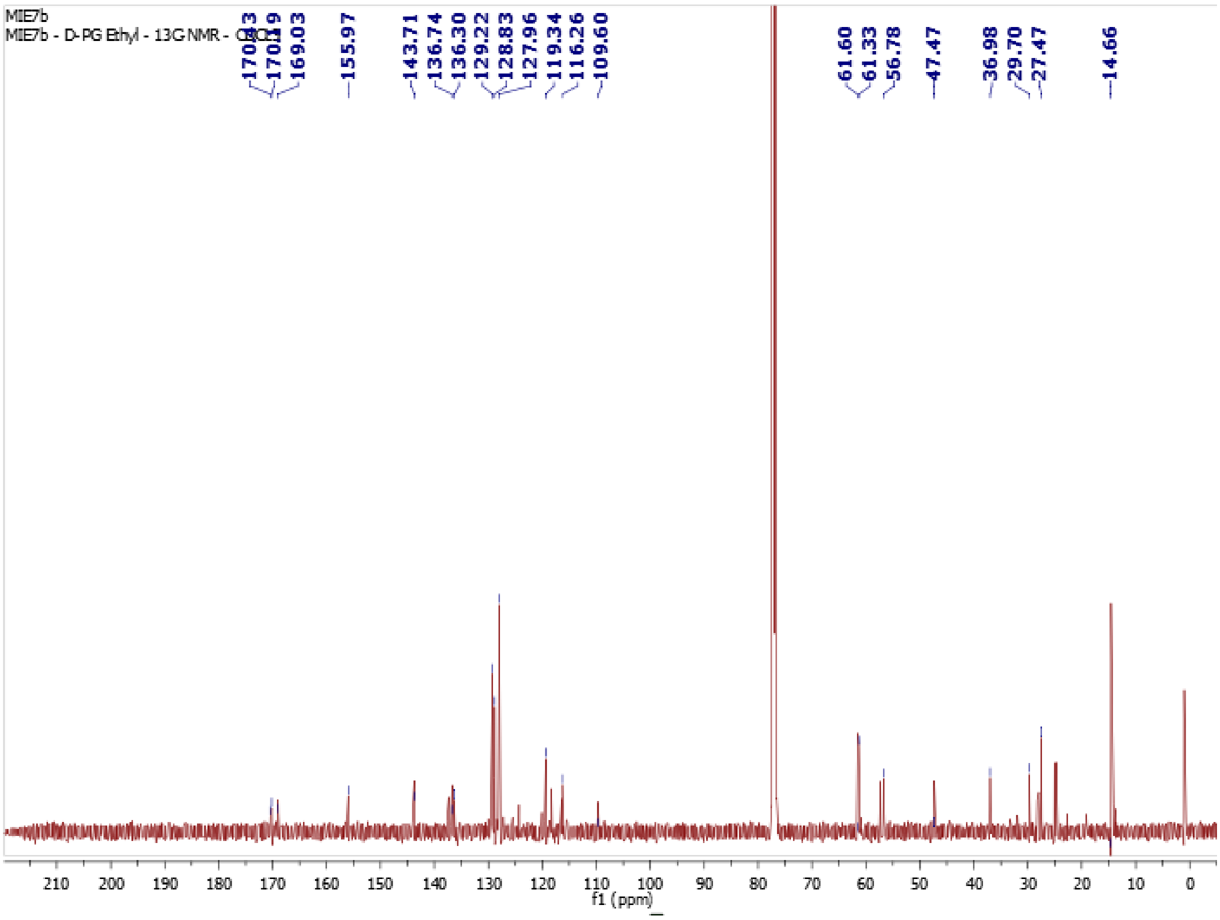

MIE7b
MIE7b - D-PG Ethyl - 13C NMR - CDCl3
170.23
170.19
169.03
155.97
143.71
136.74
136.30
129.22
128.83
127.96
119.34
116.26
109.60
61.60
61.33
56.78
47.47
36.98
29.70
27.47
14.66
210 200 190 180 170 160 150 140 130 120 110 100 90 80 70 60 50 40 30 20 10 0
f1 (ppm)

08-Mar-2019

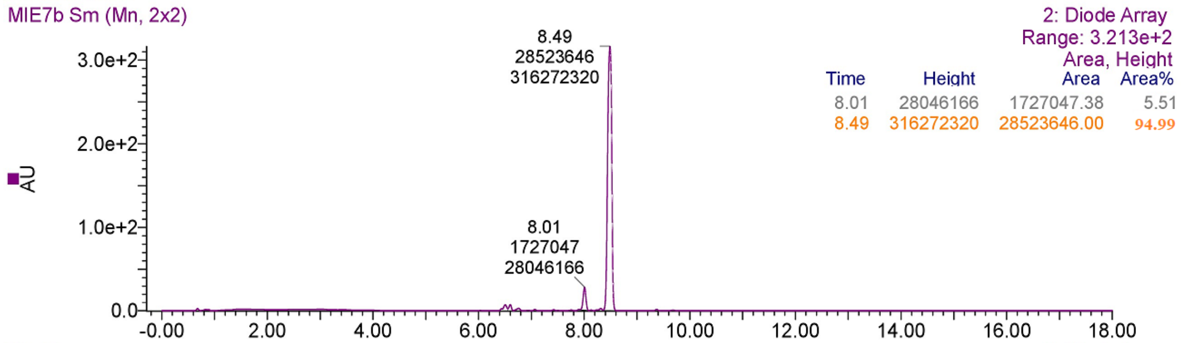
MIE7b Sm (Mn, 2x2)
2: Diode Array
Range: 3.213e+2
Area, Height
Time Height Area Area%
8.01 28046166 1727047.38 5.51
8.49 316272320 28523646.00 94.99
8.49
28523646
316272320
8.01
1727047
28046166
AU
3.0e+2
2.0e+2
1.0e+2
0.0
-0.00 2.00 4.00 6.00 8.00 10.00 12.00 14.00 16.00 18.00

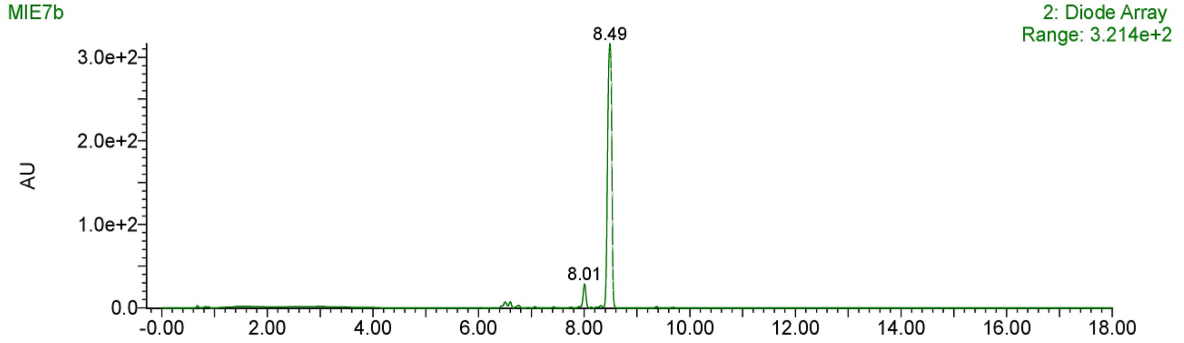
MIE7b
2: Diode Array
Range: 3.214e+2
8.49
8.01
AU
3.0e+2
2.0e+2
1.0e+2
0.0
-0.00 2.00 4.00 6.00 8.00 10.00 12.00 14.00 16.00 18.00

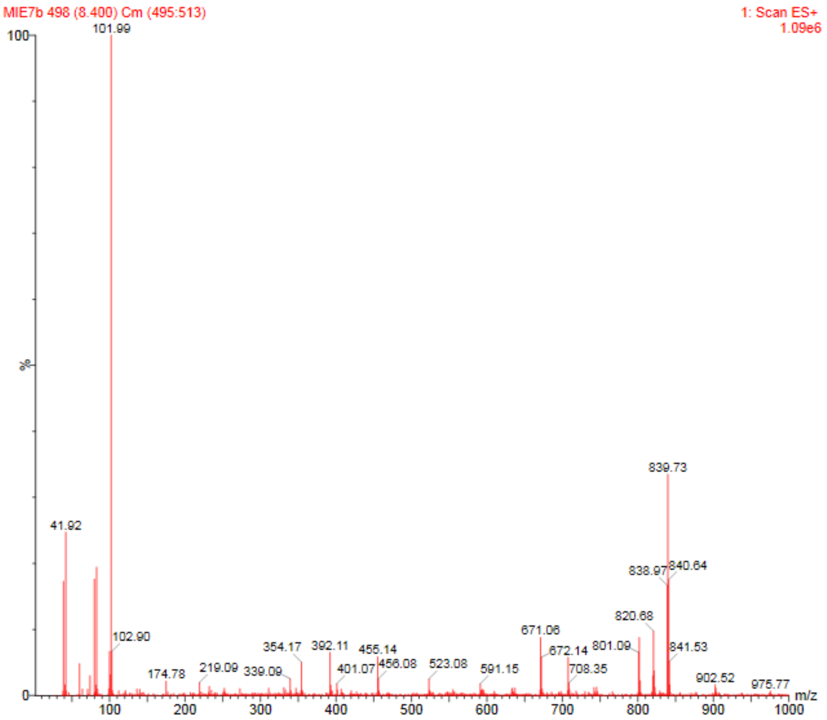
MIE7b 498 (8.400) Cm (495:513)
1: Scan ES+
1.09e6
101.99
839.73
41.92
838.97
840.64
102.90
820.68
671.06
672.14
801.09
841.53
354.17
392.11
455.14
174.78
219.09
339.09
401.07
456.08
523.08
591.15
708.35
902.52
975.77
%
100
0
100 200 300 400 500 600 700 800 900 1000
m/z

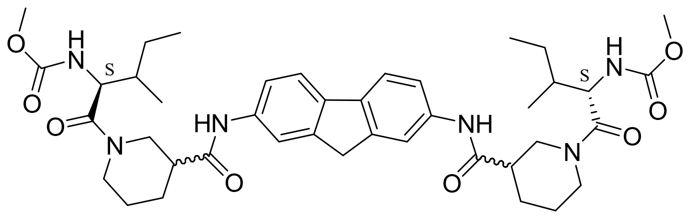

31

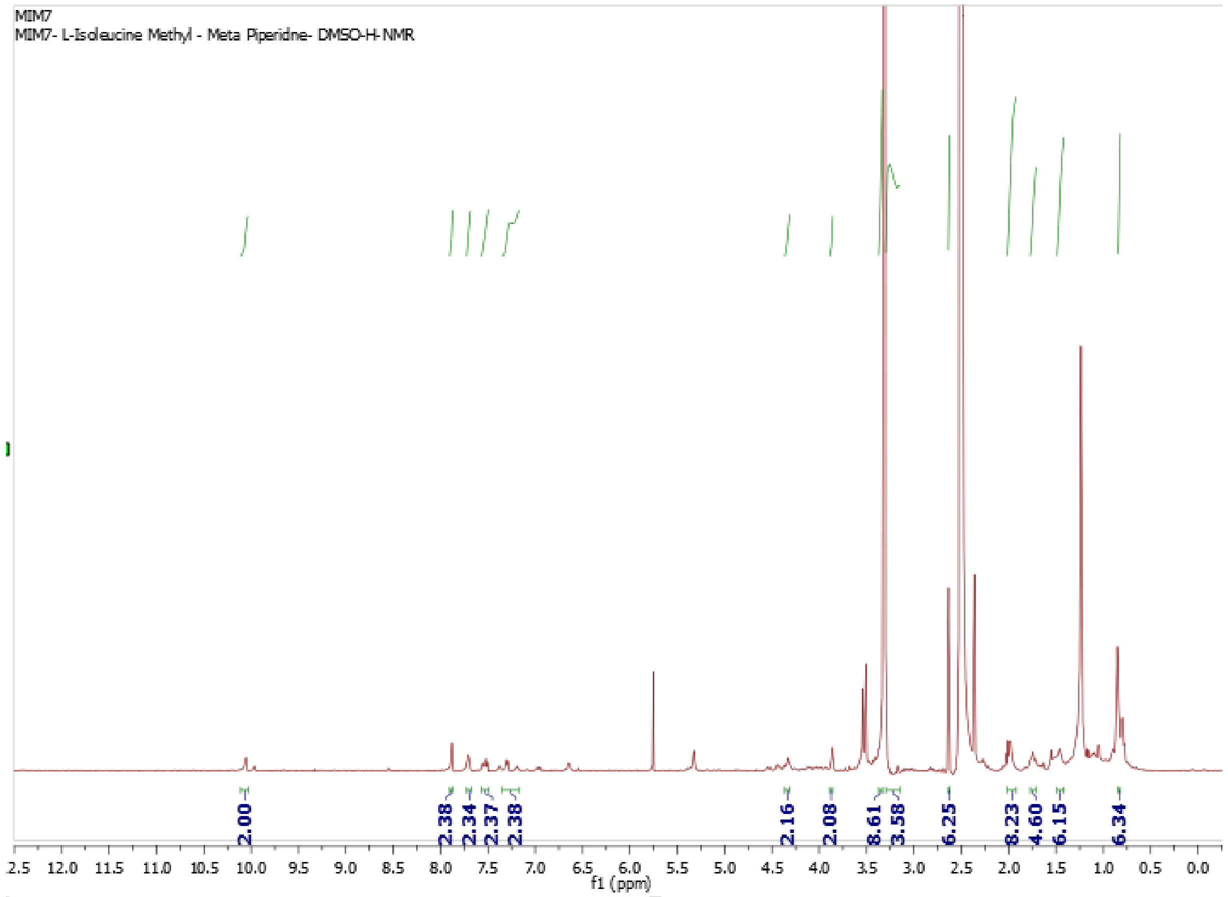

MIM7
MIM7- L-Isoleucine Methyl - Meta Piperidne- DMSO-H-NMR
2.00
2.38
2.34
2.37
2.38
2.16
2.08
8.61
3.58
6.25
8.23
4.60
6.15
6.34
f1 (ppm)

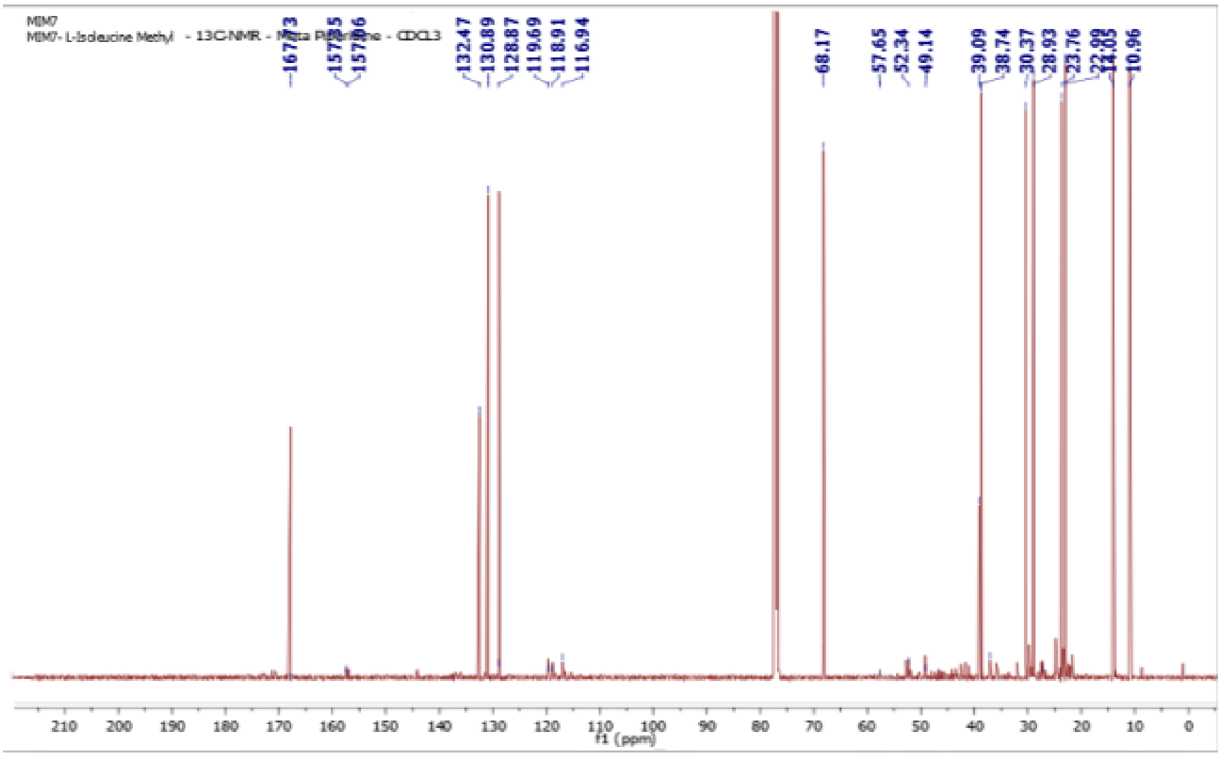
MIM7
MIM7- L-Isoleucine Methyl - 13C-NMR - Meta Piperidine - CDCl3
f1 (ppm)

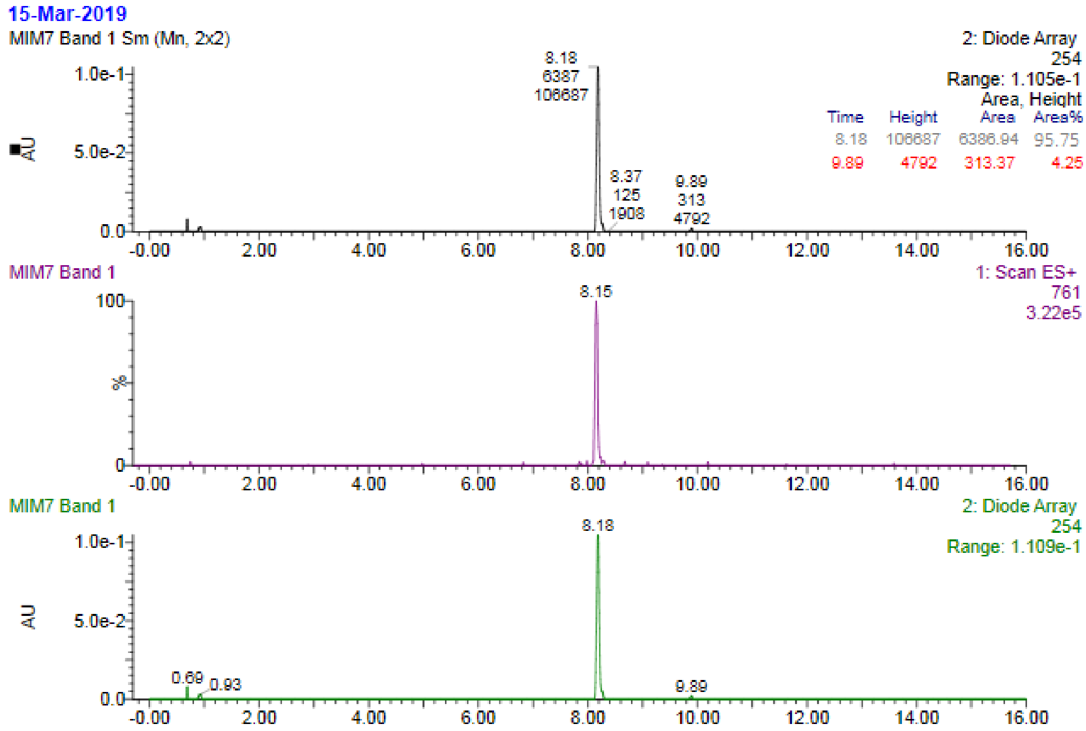
15-Mar-2019
MIM7 Band 1 Sm (Mn, 2x2)
2: Diode Array
254
Range: 1.105e-1
Area, Height
Time Height Area Area%
8.18 106687 6386.94 95.75
9.89 4792 313.37 4.25
MIM7 Band 1
1: Scan ES+
761
3.22e5
MIM7 Band 1
2: Diode Array
254
Range: 1.109e-1

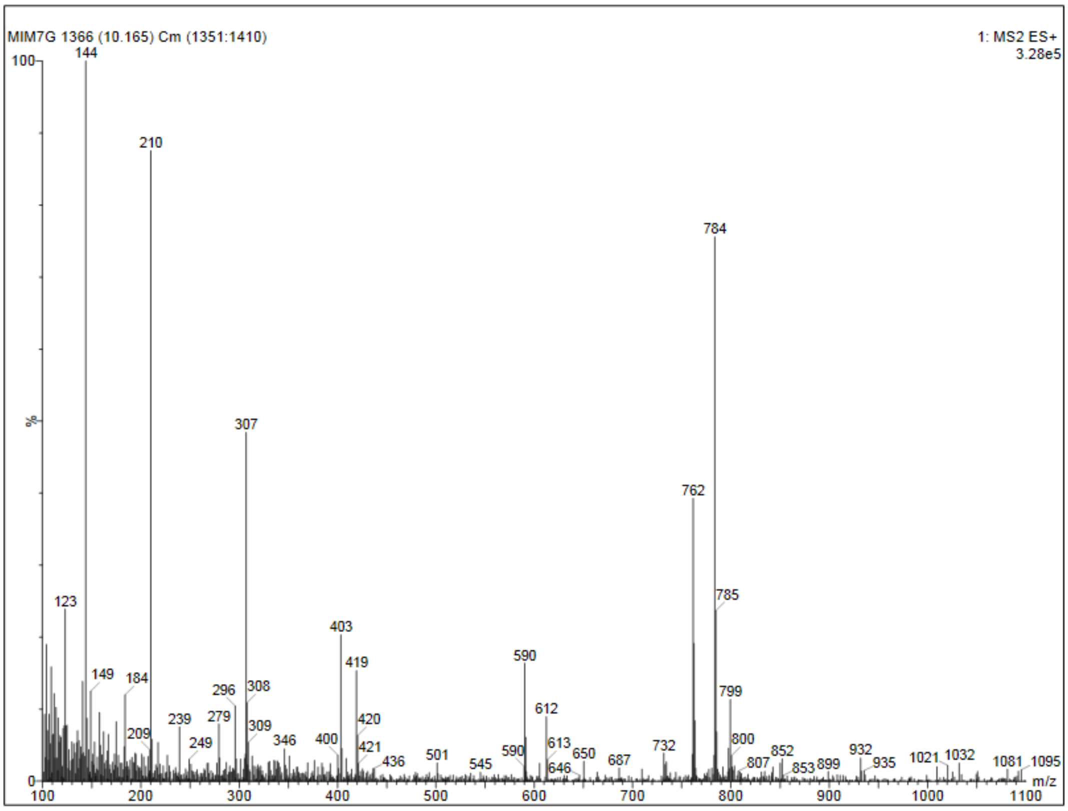
MIM7G 1366 (10.165) Cm (1351:1410)
1: MS2 ES+
3.28e5
100
%
0
144
210
784
307
762
785
123
403
419
590
149
184
296
308
799
612
239
279
420
209
309
346
400
613
800
249
421
590
650
732
852
501
687
932
1021
1032
1081
1095
436
545
646
807
853
899
935
m/z
100
200
300
400
500
600
700
800
900
1000
1100
